# **Biochemical and structural characterization of the human gut microbiome metallopeptidase IgAse provides insight into its unique specificity for the F**_**ab**_ **’ region of IgA1 and IgA2**

**DOI:** 10.1371/journal.ppat.1013292

**Published:** 2025-07-08

**Authors:** Juan Sebastián Ramírez-Larrota, Pauline Juyoux, Pablo Guerra, Ulrich Eckhard, F. Xavier Gomis-Rüth

**Affiliations:** 1 Proteolysis Laboratory, Department of Structural and Molecular Biology, Molecular Biology Institute of Barcelona (CSIC), Barcelona Science Park, Barcelona, Catalonia, Spain; 2 Synthetic Structural Biology Group, Department of Structural and Molecular Biology, Molecular Biology Institute of Barcelona (CSIC), Barcelona Science Park, Barcelona, Catalonia, Spain; 3 Graduate Program in Biotechnology, Faculty of Pharmacy and Food Sciences, University of Barcelona, Barcelona, Catalonia, Spain; 4 Methods and Electron Microscopy Group, University Grenoble-Alpes, CNRS, CEA, EMBL, ISBG, Grenoble, France; 5 Cryo-Electron Microscopy Platform, Molecular Biology Institute of Barcelona (CSIC) and Joint Electron Microscopy Center at ALBA (JEMCA), Cerdanyola del Vallès, Catalonia, Spain; National Jewish Health, UNITED STATES OF AMERICA

## Abstract

Human immunoglobulin A (IgA), comprising the isotypes IgA1 and IgA2, protects ~400 m^2^ of mucosal surfaces against microbial infections but can also lead to aberrant IgA deposits that cause disease. Certain bacteria have evolved peptidases that cleave the hinge between the F_ab_ and F_c_ fragments of IgA, undermining its immune function. These peptidases specifically target IgA1, but not IgA2, which predominates in the gut and possesses a structurally distinct hinge region. The only known IgA2-specific peptidase is IgAse from the gut microbiome member *Thomasclavelia ramosa*, which also targets IgA1 but no other proteins. IgAse is a ~ 140-kDa, seven-domain, membrane-bound metallopeptidase (MP). Differential scanning fluorimetry, small-angle X-ray scattering, AI-based structural predictions, mass spectrometry, and high-resolution crystallography and cryo-electron microscopy of multidomain fragments of IgAse revealed a novel 313-residue catalytic domain (CD) from the igalysin family within the metzincin MP clan. The CD is flanked by an N-terminal globular C-type lectin-like domain and a wrapping domain (WD), followed by four all-β domains. Functional studies involving a comprehensive set of constructs (wild-type and mutant), authentic and recombinant IgA fragments, and inhibitors demonstrated that the minimal functional assembly requires the CD and WD, along with the F_ab_ and hinge region (F_ab_’). Modelling studies suggested that the F_ab_ heavy-chain constant domain interacts with the N-terminal subdomain of the CD, positioning the hinge peptide for cleavage–a mechanism confirmed by mutational analysis. These findings open avenues for therapeutic strategies to inhibit the only known IgA1/IgA2 peptidase and to develop it for dissolving pathologic IgA deposits.

## Author summary

Human immunoglobulin A (IgA) plays a crucial role in protecting our mucous membranes from infections, yet can also contribute to disease through abnormal deposits. Certain bacteria have evolved peptidases that selectively cleave and inactivate IgA, termed IgA-peptidases, but they target only the IgA1 isotype, leaving the gut-dominant IgA2 intact. Here, we characterized IgAse, a metallopeptidase (MP) produced by the gut bacterium *Thomasclavelia ramosa* which uniquely cleaves both IgA1 and IgA2 but no other protein substrates. Employing various molecular, biochemical, biophysical, and structural approaches, including X-ray crystallography and single-particle cryo-electron microscopy, we elucidated its intricate structure and function. The enzyme features a multi-domain architecture, including a distinctive catalytic domain belonging to the metzincin clan of MPs. We found that interaction between the IgA F_ab_ fragment and IgAse is crucial for the enzyme’s ability to cleave IgA. These insights into the mechanism of IgAse open multiple therapeutic avenues, including inhibitor development for anti-pathogen strategies and novel approaches for treating diseases driven by pathological IgA depositions, such as IgA nephropathy, through the design of improved IgAse variants with enhanced functionalities.

## 1. Introduction

Immunoglobulin A (IgA) is the daily most abundantly produced human immunoglobulin (Ig) [1,2]. It is found in fluids and the external secretions that bathe mucosal surfaces [2,3], which are particularly susceptible to pathogen invasion. In the gastrointestinal mucosa, IgA both facilitates a homeostatic response to commensal bacteria and triggers an immune cell-driven antibody reaction against mucosal pathogens or vaccines [4]. Beyond the gut, however, IgA is also implicated in pathological conditions including IgA nephropathy, characterized by aberrant IgA deposits that lead to kidney dysfunction and failure [5,6]. Other conditions include IgA vasculitis, which involves the accumulation of IgA in blood-vessel walls [7], as well as IgA myeloma [8] and linear IgA/IgG bullous dermatosis [9].

**Fig 1:**
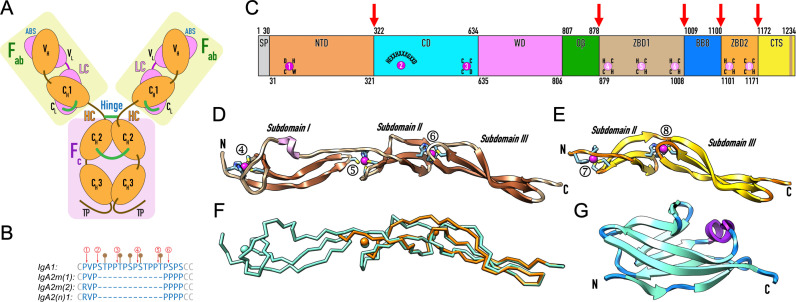
Architecture of IgA and domain structure of IgAse. (A) Monomeric IgA is a disulfide-linked heterotetramer composed of two light chains (LC, pink) and two heavy chains (HC, orange), which consist of two and four Ig-like β-sandwich domains, respectively [89]. Both LC and HC contain an N-terminal variable domain (V_L_ and V_H_) and a downstream constant domain (C_L_ and C_H_1). Together, these four domains form the F_ab_ fragment, which includes the antigen-binding site (ABS) in its variable region. The HC features an additional hinge region, flanked by cysteines engaged in disulfide bonds [11], as well as two constant domains (C_H_2 and C_H_3) and a C-terminal tailpiece (TP) that constitute the F_c_ fragment. There are three HC–LC interchain disulfides (in green) except for IgA2m(1), which lacks the C_L_–C_H_1 disulfide [12]. (B) The hinge sequences of the four IgA variants (C^101^–C^123^ for IgA1 and C^101^–C^110^ for IgA2 allotypes per UniProt P01876, P01877, and A0A0G2JMB2) reveals that only IgA1 contains five *O*-glycosylation sites (represented by brown lollipops) [5]. The cleavage sites of specific IgA peptidases are indicated with red arrows: ① *Thomasclavelia ramosa*; ② *Prevotella melaninogenica* and *Capnocytophaga sputigena*; ③ *Streptococcus* species *mitis*, *oralis, sanguinis* and *pneumoniae* and *Gemella haemolysans*; ④ *Haemophilus* species *influenzae* (encoded by the i*gaA1* gene) and *aegyptius*; ⑤ *Neisseria* species *gonorrhoeae* and *meningitidis* (i*gaA1* gene), *Ureaplasma urealyticum*, and *H. influenzae* (*igaA2* and *igaB1* genes); and ⑥ *N. meningitidis* (*igaA1* gene) and *H. influenzae* (*igaB2* gene) [5,11,14,90]. (C) *T. ramosa* IgAse contains a signal peptide (SP; M^1^–A^30^) and an N-terminal domain (NTD; A^31^–A^321^), with structural zinc ① (magenta sphere) coordinated by a cysteine, a histidine, and an aspartate. This is followed by a catalytic domain (CD; N^322^–V^634^) with catalytic zinc ② bound by the histidines and aspartate from a zinc-binding motif (HExxHxxxGxxD), and structural zinc ③ coordinated by three cysteines and an aspartate. Next in sequence are a wrapping domain (WD; D^635^–T^806^); an open β-sandwich domain (Oβ; Q^807^–N^878^); a zinc-binding domain (ZBD1; E^879^–T^1008^) encompassing putative zinc sites ④–⑥ (pale magenta spheres), each coordinated by two histidines and two cysteines; a seven-stranded β-barrel domain (BB8; A^1009^–S^1100^); a second zinc-binding domain (ZBD2; G^1101^–T^1171^) containing putative zinc sites ⑦ and ⑧, each bound by two histidines plus two cysteines and three cysteines plus a histidine, respectively; and a C-terminal segment (CTS; S^1172^–E^1234^). The latter encompasses a first linker (S^1172^–K^1195^), a sortase recognition motif (S^1196^PQT↓G^1200^), a second linker (G^1200^–N^1204^), a transmembrane helix (brown band; I^1205^–Y^1225^) and a charged C-terminal tail (N^1226^–E^1234^). The flanking residue numbers of each domain/segment are indicated above or below the bar, and red arrows pinpoint flexible hinges between domains according to SEC–SAXS. (D) Predicted model of ZBD1 with coils in tan, β-strands in sienna and a short helix in plum. The three potential zinc sites (④–⑥) are shown together with their protein ligands. (E) Same as (F) but for ZBD2, with coils in orange and β-strands in gold. The two putative zinc sites (⑦–⑧) are further displayed. (F) Superposition of the predicted Cα-traces of ZBD1 (cyan) and ZBD2 (orange) together with their putative metals as spheres). (G) Predicted model of domain BB8, with coils in dodger blue, β-strands in cyan, and the only helix in purple.

Chemically, IgA resembles other human Igs in that it consists of a heavy chain (HC) and a light chain (LC), each with a variable region (V_H_ and V_L_) and a constant region (C_H_1 and C_L_), which together form the F_ab_ fragment [2] (Fig 1A). The C_H_1 extends at the C-terminus with a flexible “hinge region,” which together with the F_ab_ forms fragment F_ab_’ and is followed by two constant domains and a tailpiece (Fig 1A). In its “monomeric” form, IgA consists of two HC–LC units forming a Y-shaped structure (Fig 1A), where the two respective C_H_2 and C_H_3 moieties make up the F_c_ fragment. In dimeric and polymeric forms, IgA monomers associate through the C_H_3 domains mediated by a joining J-chain. Such dimeric forms can migrate to the mucosal tissue upon binding to the “secretory component,” forming the secretory IgA [2]. IgA splits into two subclasses or isotypes, IgA1 and IgA2, which differ in tissue and body-fluid distribution, glycosylation profiles, and immune functions [10]. IgA2 is predominant in secretory tissues and occurs in similar quantities to IgA1 in external secretions [1]. Additionally, IgA2 is subdivided into three allotypes: IgA2m(1), IgA2m(2), and IgA2(n) [11,12]. These allotypes are significant, as the four IgA variants differ in the length and sequence of their hinge regions, which in IgA1, but not in IgA2, include five *O*-linked glycan attachment sites [5] (Fig 1B).

The antimicrobial effectiveness of IgA has driven selective pressure on bacterial commensals and pathogens, leading to the emergence of sabotaging IgA-peptidases [5,11]. The first such peptidase was identified in *Streptoccus sanguinis* (formerly *S. sanguis*) [13], with subsequent examples found in pathogens like *Neisseria*, *Pasteurella*, *Pseudomonas*, *Haemophilus*, *Prevotella* (formerly *Bacteroides*), *Capnocytophaga*, *Ureaplasma*, *Proteus*, *Mannheimia*, *Gemella*, and other *Streptococcus* species. They are serine, cysteine, or metallopeptidases (MPs) [14,15] that either generally degrade IgA or selectively cleave the hinge region between P and S/T (amino-acid one-letter code) bonds to separate the intact F_c_ and F_ab_ fragments. This cleavage prevents the F_c_-mediated signalling that triggers phagocytic killing and further results in non-functional F_ab_ fragments coating the bacterial surface, effectively shielding the bacteria from host immune surveillance [5,16]. However, these enzymes only target IgA1 because the hinge of IgA2 is much shorter, proline-rich, and lacks P–S/T bonds (Fig 1B), which makes it recalcitrant to cleavage. Consequently, they are called “IgA1 peptidases” [5,11,15]. The sole known peptidase capable of cleaving both IgA1 and IgA2 is IgAse, a unique enzyme encoded by *Thomasclavelia ramosa* (formerly *Clostridium ramosum*) [15]. This anaerobic, Gram-positive, spore-forming commensal bacterium resides in the human intestine, which produces ~5 g of IgA daily, predominantly IgA2 [1]. Thus, cleaving both IgA isotypes provides *T. ramosa* a competitive advantage in its natural habitat [15].

Classified as an MP within the M64 family according to the *Merops* peptidase database (www.ebi.ac.uk/merops; [17]), IgAse comprises 1234 residues and shows no significant sequence similarity to other known proteins. Moreover, the GC content of its coding gene (*iga*) suggests that it may have resulted from horizontal gene transfer [15]. Alongside IgA1 peptidases from *Streptococcus* spp. and *Gemella haemolysans* [16,18], which are unrelated M26-family MPs from the gluzincin clan [19,20], IgAse is the only other known MP capable of selective IgA cleavage [15]. It hydrolyses the shared P–V bond of the hinge, which explains why it targets both IgA1 and IgA2 [11,12] (Fig 1B). The proline at the **P**_**1**_ position (for nomenclature of substrate and active-site-cleft sub-sites, see [21,22]) is essential for cleavage [15,23]. Consequently, while IgAm(1) is processed, IgA2m(2) and IgA2(n), which contain an arginine at this position, are not (Fig 1B).

In addition to virulence factors that disrupt the humoral immune response and thus require counteraction, bacterial IgA peptidases also hold therapeutic potential by degrading pathogenic IgA excess or deposits associated with conditions such as IgA nephropathy, vasculitis, myeloma, and linear dermatosis. A thorough understanding of these dual roles through molecular studies is essential for developing effective therapeutics. To date, the only structurally characterized IgA-cleaving MPs are the aforementioned orthologues from *Streptococcus pneumoniae* [16] and *Gemella haemolysans* [18]. Unrelated to them, we present here the first comprehensive molecular and structural studies of *T. ramosa* IgAse, which include molecular, biochemical, and biophysical studies, as well as high-resolution X-ray crystallography (MX) and cryo-electron microscopy (cryo-EM) single particle analysis (SPA). Notably, its ability to cleave both IgA1 and IgA2 highlights its promise for targeted therapeutic applications.

## 2. Results and discussion

**2.1. Modular structure of IgAse —** To understand the function of IgAse, we investigated its domain composition using both prior studies [15,23] and AI-based predictions using *AlphaFold3* [24]. Several regions exhibited limited prediction reliability, confirming that while such predictions are valuable for hypothesis generation, they cannot replace experimental structure determination [25]. Nevertheless, we identified nine distinct domains and segments (Fig 1C). These include a 30-residue signal peptide (SP), an N-terminal domain (NTD), a catalytic domain (CD), a wrapping domain (WD), an open-face β-sandwich domain (Oβ), two putative zinc-binding domains (ZBD1 and ZBD2), an eight-stranded β-barrel domain (BB8), and a C-terminal segment (CTS). The CTS (S^1172^–E^1234^; per UniProt accession code [UP] Q9AES2) is disordered/unstructured and includes a predicted sortase recognition motif (S^1196^PQT↓G^1200^), a transmembrane helix, and a charged C-terminal tail. These features suggest that IgAse is initially anchored to the bacterial cell wall [15]. However, since IgAse activity is predominantly detected in the culture supernatant of *T. ramosa,* it is likely released from the membrane through limited proteolysis. Lastly, despite the presence of 24 cysteines, no disulfide bonds were observed in either experimental structures (*vide infra*) or the predicted models, while up to eight zinc-binding sites have been identified or predicted (Fig 1C), with 15 cysteines involved in their structural coordination.

**2.2. Analysis of the ZBD1, ZBD2 and BB8 models —** Structural AI-based predictions for the isolated ZBD1, BB8, and ZBD2 modules were classified as “confident” (see Section 3.1) based on sequence coverage and predicted local-distance difference test (pLDDT) scores of 87, 88, and 85, respectively. The ZBD1 model spans 129 residues (E^879^–T^1008^) and adopts an elongated shape of ~110 Å in length (Fig 1D). It consists of three subdomains, I (E^879^–G^934^), II (H^935^–H^968^), and III (T^969^–T^1008^), each featuring a three-stranded antiparallel β-sheet with simple up-and-down connectivity, characterized by varying lengths, twists, and curvatures. Subdomain I includes a crossover loop and a short helix following the third strand, while subdomain II features a bulged third strand. Three potential metal-binding sites (sites ④–⑥; Fig 1C and 1D) were identified at the interfaces of the subdomains, tentatively assigned as structural zinc sites based on tetrahedral ligand geometry and coordination by two histidine and two cysteine residues. Despite being surrounded by different protein scaffolds, these sites resemble those found in zinc fingers, hooks, and clasps [26]. They contribute to domain stabilization in the absence of disulfide bonds, which are typically present in calcium-binding EGF-like modules [27], and reduce inter-domain flexibility. The predicted 70-residue ZBD2 model (G^1101^–T^1171^; Fig 1E) closely resembles ZBD1, sharing a root-mean-square deviation (*rmsd*) of 1.1 Å and 45% sequence identity (Fig 1F), but it just contains subdomains II (G^1101^–H^1132^) and III (S^1133^–T^1171^). It includes the predicted zinc-binding sites ⑦ and ⑧, with the latter uniquely coordinated by three cysteines and one histidine. Structure similarity searches with *Dali* [28] revealed no close homologs of ZBD1 or ZBD2, although subdomain I bears a distant resemblance to the C-terminal zinc-binding domain of human transcription elongation factor A [29]. Overall, ZBD1 and ZBD2 may represent a novel metal-binding fold, pending experimental validation.

Located between the ZBDs, the predicted model of the 91-residue BB8 (E^1009^–S^1100^) is characterized by a predominantly antiparallel eight-stranded Greek-key β-barrel with an open lateral face (top in Fig 1G). Its core adopts a seven-stranded c-type Ig-like fold [30], reminiscent of Ig constant domains such as those found in IgA (Fig 1A), augmented by a small extra β-strand following the third strand. A short helix in the crossover loop connects strands five and six, while the first strand, interrupted by a bulge, runs parallel to the C-terminal strand. Structural similarity searches revealed homology to various Ig-like domains engaged in domain spacing, ligand binding, and elasticity. In summary, BB8, along with the flanking ZBDs, may serve as a spacer or hinge, positioning the MP domain away from the cell wall (see Section 2.8). Alternatively, they may be involved in protein binding in soluble IgAse, but not in substrate binding or catalysis (see Section 2.3).

**Fig 2:**
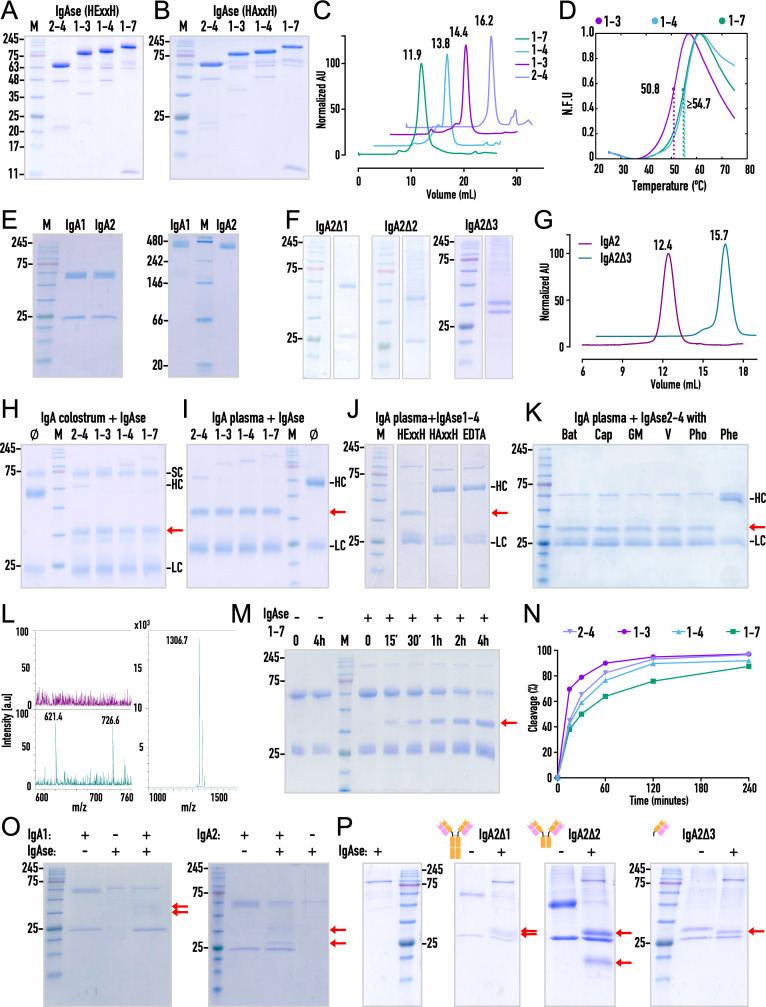
Protein production, purification, and activity characterization. Reducing SDS-PAGE analysis of (A) active (HExxH) and (B) inactive E^540^A-mutant (HAxxH) IgAse**1–3**, IgAse**2–4**, IgAse**1–4**, and IgAse**1–7** following IMAC and SEC purification. (C) SEC profiles of E^540^A-mutant IgAse**1–7** (green), IgAse**1–4** (blue), IgAse**1–3** (purple), and IgAse**2–4** (slate blue) using a Superdex 200 10/300 GL column. All protein preparations exhibited monodisperse profiles with retention volumes corresponding to their expected monomeric states in calibrated SEC. (D) Differential scanning fluorimetry denaturing curves of IgAse**1–7** (green), IgAse**1–4** (blue), and IgAse**1–3** (purple). IgAse**1-3** has lower thermal stability due to the absence of the Oβ domain. (E) Reducing SDS-PAGE (*left*) and native PAGE (*right*) analysis of in-house produced anti-HER2/neu IgA1/κ and IgA2/κ after Jacalin- and Peptide M-affinity chromatography, respectively. (F) Reducing SDS-PAGE analysis of anti-Phl p 7 IgA2/λ variants Δ1, purified by Peptide M-affinity chromatography, and Δ2 and Δ3, purified via their C-terminal His_6_-tags. (G) Analytical SEC using a Superdex 200 5/150 GL column of anti-HER2/neu IgA2 and anti-Phl p 7 IgA2Δ3 confirms proper folding and monodispersity of the samples. (H) Reducing SDS-PAGE analysis of human colostrum IgA (3 μg) incubated with IgAse variants (0.2 μg) overnight results in a specific single cleavage within the IgA HC (red arrow), while the LC and the secretory component (SC) remain intact. (I) Same as (H) for human plasma IgA (2 μg). (J) Reducing SDS-PAGE analysis of IgAse**1–4** (0.2 µg) in front of human plasma IgA (2 μg) for the wild type, the E^540^A-mutant, and the wild type plus the metal chelator EDTA (10 mM). (K) The effect of small-molecule MP inhibitors batimastat, captopril, GM1489, MMP Inhibitor 5, phosphoramidon, and the general zinc-chelator *o*-phenanthroline on IgAse**2–4** (0.2 μg) activity against plasma IgA (2 μg). Only the latter inhibits and prevents cleavage of the HC (red arrow). (L) MALDI-TOF spectra of a peptide mimicking the IgA1 hinge region (see Section 3.4) after overnight incubation with (aquamarine) or without (purple) IgAse**1–4**. The right panel (m/z range 900–1600) shows the intact, non-cleaved peptide at 1306.7 Da (monoisotopic [M + H]+). The left panel (m/z range 580–760) displays only minimal cleavage (bottom spectrum), with peaks at 621.4 and 726.6 corresponding to the putative cleavage products VTVPSP ([M + H + Na]+) and VPSTPAR ([M + H]+), respectively. (M) Reducing SDS-PAGE analysis of the time-dependent cleavage of human plasma IgA (2 µM) incubated with (+) IgAse**1–7** (0.1 µM). (N) Graph depicting time-resolved IgA cleavage by IgAse variants, quantified as cleavage percentage per time point, which was derived from reducing SDS-PAGE gels, as shown in (M) for IgAse**1–7**. Gels for other tested IgAse variants are presented in S6 Fig. (O) Single cleavage of recombinant IgA1 and IgA2 (anti-HER2/neu; 0.2–0.8 μg) by IgAse**2–4** (0.1 μg) occurs in the HC, similar to purified IgAs, confirming their structural integrity. (P) Reducing SDS-PAGE analysis showing quantitative cleavage in the hinge of C-terminally truncated IgA variants IgA2Δ1, IgA2Δ2, and IgA2Δ3 (0.2–2 μg) after incubation with IgAse**1–4** (0.2 μg; *left* panel).

**2.3. Recombinant production and biophysical assessment of IgA1, IgA2 and IgAse —** To investigate the interplay between IgAse and IgA, we cloned a panel of seven peptidase constructs (S1 Table), which included the wild-type CD and the inactive E^540^A-variant, in which the glutamate acting as general base/acid for catalysis in most MPs [20] (hereafter “catalytic glutamate”) was replaced with alanine. We also produced five additional constructs comprising only ancillary domains. We established a high-yield expression platform for recombinant overexpression in *Escherichia coli* cells, coupled with a purification protocol based on immobilized metal-chelate affinity chromatography (IMAC) and size-exclusion chromatography (SEC). This pipeline yielded between 5 and 7 mg of pure protein per litre of culture (Section 3.2) for variants that included the NTD (IgAse**1**), NTD + CD + WD (IgAse**1–3**), NTD + CD + WD + Oβ (IgAse**1–4**), CD + WD + Oβ (IgAse**2–4**), CD + WD + Oβ + ZBD1 (IgAse**2–5**), and the entire ectofragment preceding the CTS (IgAse**1–7**) (Figs 2A–C, S1–S3, and S1 Table). Notably, constructs lacking the third domain, such as IgAse**1–2**, exhibited abnormal elution profiles in SEC, underscoring the importance of this domain for structural integrity and IgAse folding. Thus, we designated it as the “wrapping domain” (see Section 2.1). Furthermore, the inclusion of reducing agents during SEC was crucial for maintaining or restoring the monodispersity of IgAse (S4 Fig). Alternatively, the addition of β-mercaptoethanol (β-ME) during lysis and IMAC purification also effectively ensured monodisperse behaviour during SEC. Under these conditions, all proteins eluted at volumes consistent with their monomeric forms (Figs 2C and S1–S3) and remained monodisperse after being frozen, stored at -80 °C, and thawed for further studies. We hypothesize that this behaviour may result from non-specific disulfide bond formation involving C^251^ in the NTD, which was modified by β-ME in our cryo-EM structure (see Section 3.9). Next, unexpected cleavage was observed in both active and inactive IgAse**1–7** (Fig 2A and 2B), suggesting the involvement of an exogenous peptidase. This cleavage produced a band of ~11 kDa, and Edman sequencing identified the cleavage site as G^1049^–S^1050^ within the BB8 domain. Given that the two fragments co-purified throughout IMAC and SEC (S2C Fig), we concluded that the cleavage did not compromise the structural integrity of the affected domain and was unlikely related to the release mechanism of IgAse from the bacterial surface. Finally, differential scanning fluorimetry, also known to as thermofluor method (TFM [31]), of the distinct protein variants revealed high stability over a pH range of 5.5–7.5, with mid-transition temperatures (*T*_m_) of ~48–55 ºC (Figs 2D and S5). At pH 4.5, a decrease of ~10 ºC with respect to these values was recorded. Notably, a relative difference of 3.9 °C was measured between IgAse**1–3** and IgAse**1–4**, indicating a stabilizing effect of the Oβ on the CD, in addition to the WD.

To provide substrates for IgAse, we purchased IgA purified from plasma, which is mainly monomeric and contains predominantly IgA1, and colostrum, which is chiefly dimeric and contains IgA1 and IgA2 in addition to the secretory component and the J-chain. Additionally, we cloned and established robust expression systems using human Expi293F cells, followed by specific affinity protocols for each protein (see Section 3.3 and Fig 2E and 2F), for a series of IgA1 and IgA2 variants. These included full-length IgA1 and IgA2m(1) (hereafter referred to as IgA2), as well as C-terminally deletion variants of IgA2, i.e., IgA2Δ1 (without the tailpiece), IgA2Δ2 (without the tailpiece and C_H_3), and IgA2Δ3 (lacking the tailpiece, C_H_3, and C_H_2), which thus encompasses the F_ab_’ fragment. Consistent with previous observations for IgG [32], the expression and purification of IgA2 constructs lacking the V_H_ and LC were unsuccessful. Finally, SEC confirmed that both full-length IgA2 and the wild-type IgA2Δ3 variant were monodisperse and monomeric (Fig 2G), with retention volumes for full-length IgA2 comparable to those reported for IgG and IgE on the same type of gel filtration column [33]. Thus, we concluded that the recombinant IgA preparations were properly folded and thus viable candidates for IgAse activity testing.

**2.4. Cleavage activity and inhibition of IgAse —**Consistent with previous reports indicating that IgAse does not cleave other Igs or common protein substrates, such as unstructured polypeptides, serpins, albumin, fibrinogen, and various collagens [15,23], we did not observe activity against casein or collagen. Consequently, we focused on authentic human colostrum and plasma IgA, which were incubated with IgAse**1–7**, IgAse**1–4**, IgAse**1–3**, and IgAse**2–4**. Selective cleavage of the HC yielded the F_c_ plus the upstream hinge for both substrates using wild-type variants (Fig 2A and 2B), but not with inactive E^540^A variants (Fig 2J), consistent with IgAse being an MP [20]. Moreover, we observed no significant difference in the overall cleavage of plasma and colostrum IgA, indicating that neither the secretory component nor the J-chain of secretory IgA affects substrate recognition. The glycosylation differences between IgA1 and IgA2, primarily found in the hinge region, also did not impact cleavage. Finally, SEC analysis of the pre-incubated mixture of plasma IgA and inactive IgAse**1–7** + E^540^A revealed no stable complex (S7A Fig), suggesting transient complex formation and/or weak substrate affinity (>10 µM) [34].

To investigate the inhibition of IgAse, we tested a cohort of small-molecule inhibitors targeting disparate MP families with IgAse**2–4**, including batimastat, captopril, GM1489, MMP inhibitor V, phosphoramidon, and *o*-phenanthroline (at 1mM). Additionally, EDTA (at 10 mM) was tested with IgAse**1–4**. Only EDTA (Fig 2J) and *o*-phenathroline (Fig 2K) efficiently prevented IgA cleavage, as expected for an MP. Thus, we found no specific inhibitors beyond non-specific metal chelators, which complements previous studies showing that IgAse is resistant to a wide range of serine- and cysteine-peptidase inhibitors [15,23].

To explore the peptidolytic capacity of IgAse, we designed a peptide mimicking the IgA1 hinge region and incubated it with IgAse**1–4** (Section 3.4), monitoring the reaction by MALDI-TOF mass spectrometry. Only minimal cleavage of the substrate (<1%) was observed after overnight incubation at 37 °C. Two low-intensity peaks (ion intensity of ~80 units) corresponding to the putative cleavage products were detected, which were absent in controls with the peptide (ion intensity of ~20,000 units) and peptidase alone (Figs 2L and S7B). This indicates that IgAse does not really recognize peptides with the target sequence but requires a folded protein substrate.

Next, we conducted time-course experiments to determine the impact of the non-catalytic domains on the cleavage of human plasma IgA (Figs 2M and S6). Strikingly, IgAse**1–7** exhibited the lowest activity, while IgAse**1–3** showed the highest, with IgAse**1–4** and IgAse**2–4** performing at intermediate levels (Fig 2N). This suggests that IgAse**2–3** (CD + WD) represents the minimal catalytically functional unit, and that flanking domains NTD and Oβ are dispensable. The accessory domains downstream of the Oβ appear to hinder turnover *in vitro*, potentially obstructing substrate binding or access to the active site. However, since the Oβ domain conferred higher thermal stability in TFM (Section 2.3), we chose IgAse**1–4** and IgAse**2–4** for subsequent studies.

Finally, to determine the minimal substrate requirements for IgA recognition and cleavage by IgAse, we incubated monoclonal IgA1 and IgA2 (Section 2.3) with IgAse**2–4**, which resulted in cleavage in both cases (Fig 2O). This supports that our recombinant preparations in human cells had yielded well-folded functional IgAs, consistent with SEC analysis (Fig 2G). We also analysed the wild-type C-terminal deletion variants IgA2Δ1, IgA2Δ2, and IgA2Δ3, and found that all three were likewise efficiently cleaved by IgAse**1–4** (Figs 2P and S10). This accounts for IgA2Δ3, which encompasses F_ab_’, meeting the minimal structural requirements for IgAse recognition and cleavage. Since the V_H_ domain differs in polyclonal IgA1/IgA2 preparations from native sources, which are both efficiently processed (*vide supra*), the primary substrate recognition interface must reside within the C_H_1 domain, apart from its interaction interface with C_L_.

**Fig 3:**
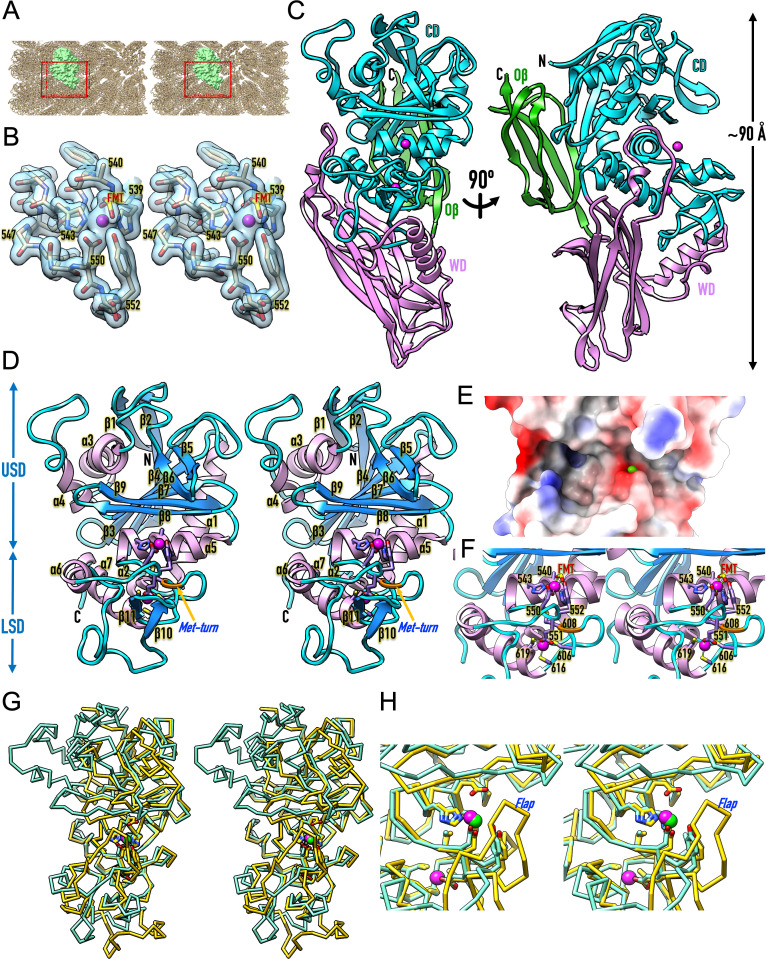
Structure of the IgAse catalytic core. (A) Crystal packing of IgAse**2–4** monoclinic crystals viewed down the crystallographic cell axis a in cross-eye stereo. The protomer in the asymmetric unit is represented as a pale green surface model, while symmetry mates are shown as Cα models in tan. The unit cell is outlined in red. (B) A representative section of the final 1.75-Å (2*mF*_obs_–*DF*_calc_)-type Fourier map contoured at 1 σ, displaying segment H^539^–Y^552^ in stick representation with carbons in tan and the catalytic zinc as a magenta sphere. The formate molecule (FMT) attached to the zinc is depicted with the carbon in gold. (C) Ribbon plot of IgAse**2–4** from MX, illustrating the CD (cyan; zinc cations as magenta spheres), the WD (purple), and the Oβ domain (green) in standard orientation for MPs [22] (*left*) and an orthogonal view (*right*). (D) Stereo ribbon plot of the CD, with coils in cyan, β-strands in dodger blue (labelled β1–β11), helices in plum (labelled α1–α7), and the Met-turn in orange. The CD is divided into an upper USD subdomain and a lower LSD subdomain, which contain a catalytic zinc site (top magenta sphere) and a structural zinc site (bottom magenta sphere). The respective metal-binding side chains and the Met-turn methionine are displayed. (E) Surface model of the CD centred on the active-site cleft, coloured according to electrostatic potential computed with *ChimeraX*. The catalytic zinc is represented as a green sphere. (F) Close-up of (D) in stereo, highlighting the two zinc sites; the metal-binding residues and the Met-turn methionine are labelled. (G) Stereo superposition of the Cα traces of the IgAse CD (aquamarine; zinc ions in magenta) in the orientation of (D) and the putative MP from *Bacillus ovatus* (PDB 3P1V) (gold/green). (H) Close-up of (G) after a vertical rotation of 45º, showing the large flap occluding the active-site cleft in the *Bacillus* protein.

**2.5. Crystal structure of the IgAse catalytic domain —** We conducted thousands of crystallization assays for IgAse**1**, IgAse**1–3**, IgAse**1–4**, IgAse**1–7**, IgAse**2–4**, and IgAse**2–5**, including transient complexes with various IgA1 and IgA2 constructs. However, we only achieved well-diffracting crystals for unbound IgAse**2–4**. The most successful crystals were those of IgAse**2–4** + E^540^A, consistent with previous findings that replacing the catalytic glutamate with alanine or glutamine produced more homogeneous inactive variants while preserving the active-site environment for structural studies [20]. The crystals were densely packed, containing 42% solvent compared to the average 51% for protein crystals (Fig 3A), which accounts for the high resolution of the diffraction data and the resulting Fourier maps (1.75 Å; see Fig 3B and S2 Table) [35,36]. The structure has an elongated shape with maximal dimensions of 90 × 65 × 50 Å (Fig 3C) and comprises the 313-residue CD (defined for E^330^–V^634^), the 172-residue WD (D^635^–T^806^), and the 70-residue Oβ (Q^807^–N^876^), which is consistent with the aforementioned results from SEC and TFM showing that the two flanking domains significantly contribute to CD stability.

The CD is asymmetrically divided into a larger upper subdomain (USD; E^330^–L^546^) and a smaller lower subdomain (LSD; G^547^–V^634^) (Fig 3D) by a horizontal, deep active-site cleft that traverses the entire domain and extends from the non-primed to the primed side in the standard orientation of MPs [22] (Fig 3D and 3E). The USD features a central, strongly twisted seven-stranded mixed parallel/antiparallel β-sheet (β1 ↑ –β2 ↓ –β4 ↑ –β3 ↑ –β7 ↑ –β9 ↑ –β8 ↓ ; top-to-bottom in Fig 3D; independent β-strand and α-helix numbers for each IgAse domain), with the lowest strand (β8) forming the “upper-rim strand” that frames the active-site cleft ceiling. Strands β4 and β7 are linked by an 85-residue segment above the sheet, which incorporates helices α3 and α4, as well as the β-ribbon β5β6. Two additional helices (α1 and α2) connect β3 and β4 beneath the sheet. Following the last sheet strand (β9), the chain leads to helix α5, which frames the back of the active-site cleft.

This USD architecture ascribes IgAse to the metzincin clan of MPs [19,20,37–39], whose CDs range from 132 to 347 residues [20,40] and share a core architecture featuring a consensus five-stranded β-sheet, superposable onto and with connectivity equivalent to β4–β3–β7–β9–β8 in IgAse; a “backing helix” analogous to α1; and an “active-site helix” corresponding to α5. Metzincins are categorised into at least 17 families [19,38], including pappalysins and adamalysins, which exhibit an “adamalysin helix” equivalent to α3. Additionally, metzincins possess a consensus zinc-binding motif, HExxHxxG/NxxH/D, with the first seven residues located within the active-site helix. The two first histidine residues bind the catalytic zinc, while the second residue serves as the catalytic glutamate (*vide supra*) [20]. The glycine—an asparagine is found in lower pappalysins [19,41]—is the first residue of the LSD and leads to the third zinc-binding residue, typically a histidine or, rarely, an aspartate, as seen in snapalysins and thuringilysins [19]. In IgAse, which includes these features–including a catalytic zinc site (site ② in Figs 1C and 3F)–, an additional residue (L^546^) precedes the glycine, which is compensated by an extension of the helix by half a turn, aligning the chain trace with other metzincins. Moreover, the third ligand in IgAse is also an aspartate (D^550^), resulting in a unique zinc motif (H^539^E^540^xxH^543^xxxG^547^xxD^550^).

In metzincins, the LSD is generally smaller and contains fewer regular secondary structure elements than the USD, a pattern also observed in IgAse (Fig 3D). The only common elements are a methionine-containing 1,4-tight turn (“Met-turn”), which provides a hydrophobic base and stability to the catalytic zinc site [42], and a “C-terminal helix” that concludes the CD. These elements are present in IgAse, with the former spanning C^606^IM^608^R and the latter represented by helix α7 (Fig 3D and 3F). Notably, IgAse features a unique “connecting segment” (S^553^–E^605^) between D^550^ and the Met-turn, which includes a tyrosine (Y^552^) that is close to, but does not bind, the zinc (Fig 3F). Instead, it may function as a “tyrosine switch” for stabilizing reaction intermediates during catalysis [43,44]. Such a residue has also been observed in other metzincins, including astacins, pappalysins, snapalysins, serralysins, cholerilysins, and helicolysins [19,38]. The connecting segment also contains the short helix α6, the β-ribbon β10β11, and a second zinc-binding site (site ③ in Fig 1C; see also Fig 3F and 3H), which is located below the Met-turn and contributes to the overall structural integrity of the CD. The metal is coordinated by E^551^, which occupies the position of the “family-specific” residue in metzincins immediately downstream of the third catalytic-zinc ligand [19,38,43]. The other three ligands, which contribute to a tetrahedral metal-binding site characteristic of structural zinc ions [26], include the first residue of the Met-turn (C^606^) and two additional cysteines from the C-terminal helix (C^616^ and C^619^). This site is expected to stabilize the catalytic site and is also found in archaemetzincins, where the family-specific residue and zinc ligand is a cysteine, which contributes to a “Cys_4_ zinc finger” [45]. Finally, the LSD of IgAse contains a short “wall-forming segment” (D^610^–C^616^), which shapes the **S**_**1**_**’** specificity pocket [22] and links the Met-turn with the C-terminal helix [19].

The IgAse CD shows structural similarity to two putative peptidases from *Bacillus ovatus* (Protein data Bank access code [PDB] 3P1V) and *Bacillus fragilis* (PDB 4DF9). Both structures feature an N-terminal seven-stranded β-sandwich followed by a CD-like moiety of 269 residues. Superposition reveals close similarity (PDB 3P1V; Z-score = 24.5; *rmsd* = 2.4 Å; 22% identity; and PDB 4DF9; Z-score = 24.8; *rmsd* = 2.3 Å; 21% identity) (Fig 3 G and 3H). These structures were deposited in 2010 and 2012, respectively, by the late Joint Center for Structural Genomics [46]. Unfortunately, they have not been functionally analysed or published, and they were only mentioned in a review of our work in 2014 [19], where they were classified within the igalysin family (M64-family according to *Merops*) as part of the metzincin clan of MPs. This classification was based on their specific variation of the zinc-binding motif, which includes a third aspartate as a zinc ligand and an additional residue preceding the glycine. These characteristics are also present in IgAse (*vide supra*), further categorising it as an igalysin. However, a detailed inspection of the structure superposition reveals significant differences, in particular a flap blocking the active-site cleft (Fig 3G and 3H), which should preclude these enzymes from functioning as IgA peptidases.

**Fig 4:**
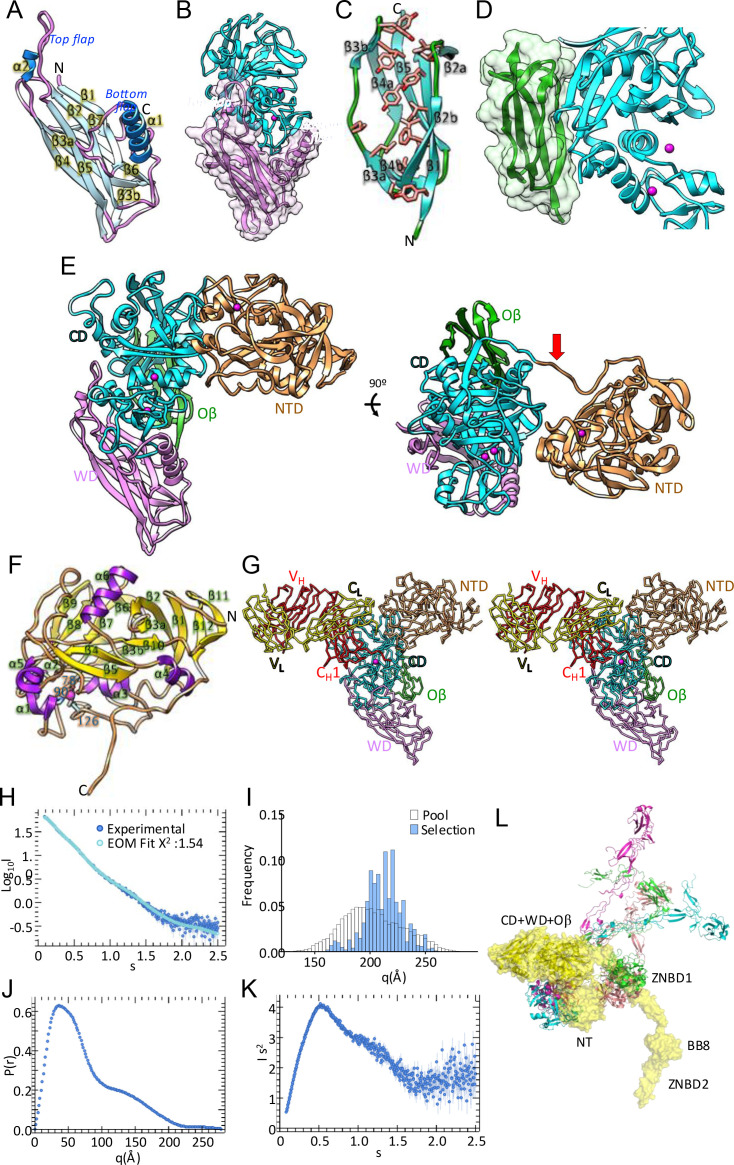
Structure of IgAse ancillary domains and small-angle X-ray scattering analysis. (A) Ribbon plot of the WD, with coils in purple, β-strands in pale blue (labelled β1–β7), and helices in dodger blue (labelled α1–α2). The two flaps protruding from the β-sandwich scaffold are labelled. (B) Cartoon of the interface between the CD (cyan) and the WD (purple), superposed with its semi-transparent Connolly surface in stereo, vertically oriented halfway between Fig 3C *left* and *right*. (C) Ribbon plot of the Oβ, with coils in green and β-strands in aquamarine (labelled β1–β5). The side chains of residues contributing to the hydrophobic core are shown with carbons in salmon. (D) Cartoon of the interface between the CD (cyan) and the Oβ (green), superposed with its semi-transparent Connolly surface, oriented similarly to Fig 3C (*right*). (E) Ribbon plot of IgAse**1–4** from cryo-EM SPA, depicting the CD, WD, and Oβ as in Fig 3C (*left*), plus the NTD in sandy brown. The first three domains are arranged as in the MX structure. In addition, an orthogonal view (*right*) depicts the NTD–CD connecting linker (red arrow) and the small NTD–CD interaction surface. (F) Ribbon plot of the NTD with coils in sandy brown, β-strands in gold (labelled β1–β12), and helices in purple (labelled α1–α6). The structural zinc (magenta sphere) is shown, together with its protein ligands D^78^, H^90^ and C^126^. (G) Cα-plot of a predicted AI-based model of IgAse**1–4** (coloured as in (E)) in a near-Michaelis complex with IgA2Δ3, with the LC in yellow and the HC in red. (H) Experimental SEC–SAXS profile of IgAse**17** (blue) and the fit obtained using *Eom* (cyan), with χ^2^ = 1.54. (I) Size distributions for the initial pool (white) and the sub-ensemble selected conformations using *Eom* (blue). (J) Graph of the P(r) distribution, which is a pair distribution function describing the probability of finding atom pairs at a given distance, for the profile shown in (H), which unveils a maximum linear dimension (d_max_) value of 278 Å. (K) Normalized Kratky plot of the SAXS profile shown in (H). (L) Ensemble of the *Eom*-selected conformations that collectively describe the experimental data, superposed for their central CD + WD + Oβ domains. The yellow surface model highlights the most abundant conformer (40%). See also S9 Fig. A summary of the SEC–SAXS data collection and the derived parameters is provided in S4 Table.

**2.6. Crystal structure of the IgAse wrapping and open **β**-sandwich domains —** The 172-residue WD, a fibronectin type-III domain [30], is C-terminally attached to the CD. It features a seven-stranded antiparallel jellyroll β-sandwich, comprising a three-stranded front sheet (β5 ↓ –β2 ↑ –β1 ↓ ; *left* to *right* in Fig 4A) and a four-stranded back sheet (β4 ↑ –β3a+β3b ↓ –β6 ↑ –β7↓). The third strand bifurcates at the bottom into β3a and β3b due to a bulge on the back surface. Notably, the WD shares structural similarity with the D3 domain of the gamete fusogen HAP2 (PDB 7LR3; Z-score = 6.6; *rmsd* = 2.4; 13% identity), which is involved in trimerization during *Plasmodium* fertilization [47]. However, D3 is substantially shorter, as the WD possesses two long protrusions on the front sheet that fold back towards the β-sandwich surface, referred to as the “bottom flap” (Y^646^–N^689^) and the “top flap” (W^743^–S^764^), each containing a helix (α1 and α2). Consequently, the WD sandwich functions as a scaffold, positioning the two flaps as clamps that reach around and thus stabilize the CD.

The WD closely wraps around the CD, primarily constraining the bottom and left faces of the LSD Figs 3C and 4B). The bottom flap interacts extensively with the external surface of the connecting segment, while the top flap approaches the left surface of the connecting segment, the C-terminal helix, and elements Lβ8β9 and α4 from the USD. This configuration shapes the primed side of the active-site cleft, influencing substrate binding. In total, 51 and 53 residues from the WD and CD form 28 hydrogen bonds and five salt bridges. The interface covers a substantial area of 2283 Å², which significantly exceeds typical peptidase–protein inhibitor interfaces (1250–1750 Å²; [48]). The calculated solvation free energy gain upon interface formation (Δ^i^G) is -21.8 kcal (*p*-value = 0.037). This close interaction between the LSD and WD, also observed in thuringilysins [49], structurally documents the necessity of the latter domain for CD folding, as constructs containing only the NTD and CD or the CD alone were insoluble (Section 2.3 and S1 Table).

Downstream of the WD, the Oβ domain is a twisted, arched five-stranded open-face β-sandwich, where strands β2, β3, and β4 are interrupted by bulges (topology β3a+β3b ↑ –β4a+β4b ↓ –β5 ↑ –β1 ↑ –β2a+β2b ↓ ; *left* to *right* in Fig 4C). The concave face of the sheet contains a hydrophobic core, open on the front face, creating a hydrophobic surface while maintaining the arch. This surface, along with flanking strands β2 and β3, interacts with the CD but not the preceding WD (Figs 3C and 4D). The interaction involves the USD, particularly the N-terminus and CD elements β2, Lβ2β3, α1, α2, and Lα2β4, with 27 and 29 residues from Oβ and CD, respectively, establishing 21 hydrogen bonds. The interface spans an area of 1033 Å, with a Δ^i^G of -5.7 kcal (*p*-value = 0.407). Consistently, CD constructs lacking Oβ were soluble, well-folded, and functional, but had lower thermal stability (Section 2.3, Fig 2D and S1 Table).

**Fig 5:**
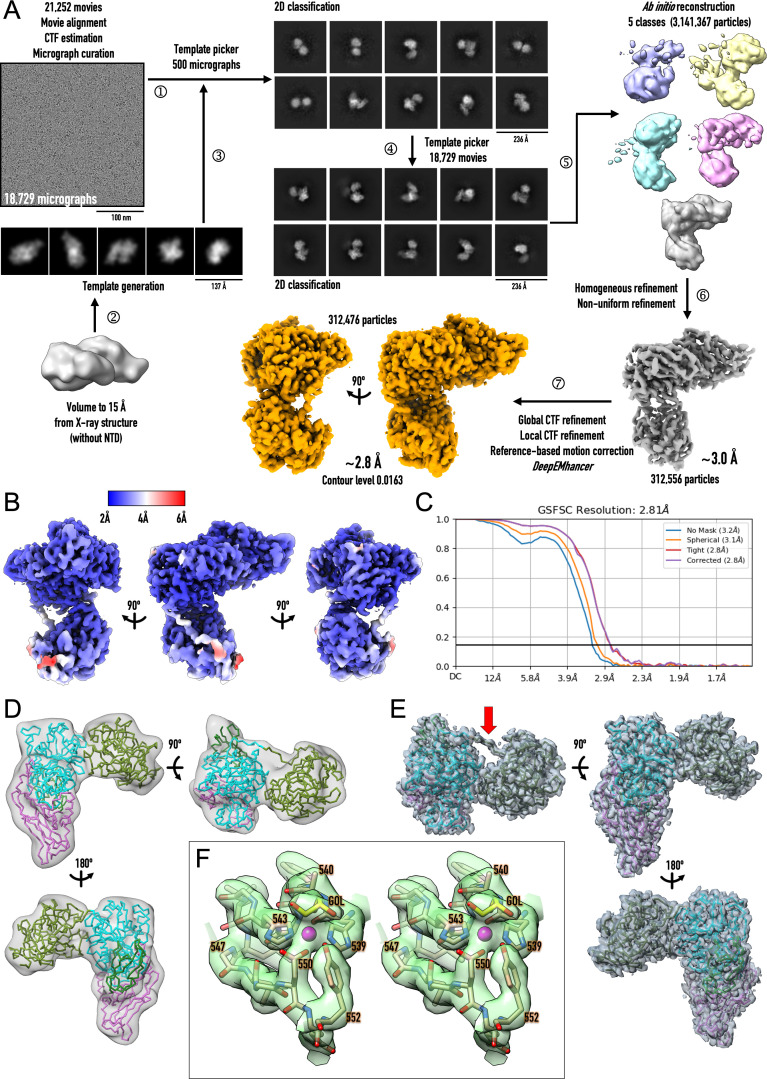
Single-particle analysis of IgAse. (A) Flowchart depicting cryo-electron microscopy single-particle analysis and image processing of the high-resolution movies from the 300-kV microscope. (B) Local refinement map (contour level 0.054), coloured by local resolution, in three orientations. (C) Gold-standard (0.143) Fourier Shell Correlation (GSFSC) curves. (D) Final low-resolution map (≥7.1 Å) from the 200-kV microscope (see S8 Fig and S3 Table) contoured at 5.7 σ in three orientations, superposed with the final structure refined against the high-resolution map shown in (E) as Cα trace. Each domain is coloured as in Fig 1C. (E) Final high-resolution *DeepEMhancer*-map (2.8 Å) from the 300-kV microscope contoured at 0.7 σ in three orientations, superposed with the final refined model as Cα trace. The domains are coloured as in (D), and the red arrow pinpoints the NTD–CD connecting segment. (F) Close-up in stereo of the catalytic zinc-binding site, showing the map of (E) contoured at 1 σ and displaying segment H^539^–Y^552^ as sticks with carbons in tan and the catalytic zinc as a magenta sphere. The glycerol molecule (GOL) attached to the zinc is depicted with carbons in gold.

**2.7. Cryo-electron microscopy structure of IgAse —** We subjected IgAse**1–7** to cryo-EM SPA, initially obtaining a middle/low-resolution Coulomb-potential map at ≥7.1 Å using a 200-kV microscope (S3 Table and Figs S8 and 5D). Subsequently, we obtained a high-resolution map at 2.8 Å with a 300-kV microscope, which enabled us to build a near-atomic structure of the NTD, CD, WD, and Oβ domains (S3 Table, Fig 5A–C, 5E and 5F). No map density was observed beyond Oβ due to the intrinsic inter-domain flexibility of the sample (see Section 2.8). At 96 kDa, this structure represents the only SPA structure of a peptidase—either alone or in a complex—under 100 kDa in the Electron Microscopy Data Bank (EMDB; www.ebi.ac.uk/emdb) at a resolution of ≤2.8 Å, out of >57,000 entries (as of 22.2.2025). Structural analysis of samples below ~100 kDa remains highly challenging due to low contrast, poor signal-to-noise ratios, and a lack of distinct features [50,51], and resolutions below 3 Å, which enable accurate atomic model building, constitute only ~11% of EMDB entries [52].

The entire polypeptide chain (S^32^–N^876^) is continuously and unambiguously resolved in the map (Fig 5E and 5F). A comparison of the CD + WD + Oβ moieties common to the MX and SPA structures reveals that they are essentially identical (core *rmsd* = 0.61 Å), except for the surface loop T^480^–S^485^, which we attribute to crystal packing. Moreover, we identified a glycerol molecule attached to the catalytic zinc (Fig 5F) instead of the formate of the crystal structure (Fig 3B). The main difference in the structures lies in the presence of the NTD (Figs 4E, 4F and 5E), which was defined at a slightly lower resolution than the adjacent CD + WD + Oβ core (Fig 5B). This indicates some flexibility and may help explain why IgAse**1–4** did not crystallize.

NTD adopts an irregular α/β fold encompassing twelve short β-strands (β1–β12) and six short helices (α1–α6) (Fig 4F), forming a central C-type lectin-like domain (CTLD) core [53], similar to the C-terminal domain of the *Legionella* collagen-like protein (PDB 8Q4E [54]; Z-score = 7.4, *rmsd* = 4.0 Å, 13% identity with the IgAse NTD). CTLDs share a characteristic asymmetric core containing two helices and six β-strands, which are cross-linked by two or three disulfides, along with a specific long loop grafted into this core for calcium and/or carbohydrate binding [53]. The NTD contains the six strands (β2, β3, β5, and β6–β9) and the first helix (α3) but lacks the second helix, the long loop, and the tethering disulfides. Instead, it is decorated with large additional irregular segments, resulting in a unique surface and a size that is double that of a typical CTLD. A structural zinc-binding site (site ① in Fig 1C), with D^78^ (bidentately), H^90^ and C^126^ as ligands (Fig 4F), is located outside the CTLD core. Overall, these differences from canonical CTLDs point to different functions for the NTD.

The NTD is connected to the CD through an extended linker (S^315^–D^324^) (Fig 4E, *right*), which is well-defined in the map (Fig 5E). It contacts the CD on the right surface of its USD (Fig 4E) through a small surface spanning 424 Å^2^ (linker excluded), but not the WD and Oβ. Contacts are primarily made between Lβ3α1 of the NTD and Lβ4β5 of the USD, as well as through α5 and Lα5β5 of the NTD with Lβ5β6, Lβ7β8, and Lβ9α5 of the USD, involving a total of 12 NTD residues and 15 USD residues. Overall, this minimal interaction may explain why the NTD is not required for CD stability, and IgAse constructs lacking the NTD are comparably active to those that include it (S1 Table, Figs S6, 2A and 2B).

**2.8. Small-angle X-ray scattering of IgAse —** Small-angle X-ray scattering (SAXS) in solution after SEC (SEC–SAXS) of IgAse**1–7** (see Figs 4H–L and S9, and S4 Table for details on data collection and derived parameters) revealed normalized Kratky-plot characteristics of a folded protein with partial flexibility (Fig 4K). The analysis yielded a pair distribution function P(r) with a maximal diameter (d_max_) of 278 Å (Fig 4J), a radius of gyration (*Rg*) of 6.43 ± 0.06 nm, and an estimated molecular weight of 118.8 kDa, which compares well with the calculated molecular weight of 127.9 kDa. To further assess molecular flexibility, we performed ensemble modelling with inter-domain linker regions identified based on low pLDDT scores (<70) in the *AlphaFold3* prediction (Section 3.1). This approach resulted in a good fit to the experimental data, with a χ^2^ value of 1.54 (Fig 4H). As highlighted in Fig 4I, the selected conformation ensemble exhibited a narrower distribution than the diverse pool used for initial sampling, indicating reduced macromolecular flexibility compared to a random pool. Thus, IgAse, while not completely rigid, adopts a limited range of conformations in solution, which would be consistent with the predicted zinc-binding sites at inter-domain interfaces rigidifying the assembly. The ensemble also showed a tendency towards semi-extended conformations, with increased conformation frequencies in the 200–230 Å range. This suggests that ZNB1, BB8, and ZNB2 may function as spacer domains to extend the CD from the bacterial cell surface, similar to the surface-bound lectin-like protein SraP from *Staphylococcus aureus* [55]. The final selected conformations (Fig 4L) provide a good fit with the experimental data and display an average *Rg* and d_max_ of 5.69 nm and 205 Å, respectively, which are consistent with the expected overall size and shape of IgAse in solution. The observed flexibility, particularly between the NTD and CD, and beyond the Oβ domain, likely accounts for constructs encompassing domains downstream of Oβ being refractory to crystallization and SPA (see Sections 2.5 and 2.7). It also explains why a construct comprising NTD–CD + WD + Oβ did not crystallize but could be solved by cryo-EM SPA, which is less sensitive to limited flexibility than crystallography [56].

**2.9. Conclusions —** IgA is the most abundant Ig produced in humans. It plays a central role in protecting mucosal tissues against microbial infection; however, its aberrant accumulation can lead to various pathologies, including IgA nephropathy, vasculitis, myeloma, and dermatosis. Certain commensal and pathogenic bacteria have evolved peptidases that specifically inactivate IgA at the hinge, thereby circumventing the immunogenic response and facilitating their survival and adaptation within the human mucosa.

*T. ramosa* IgAse belongs to an MP family distinct from the only other well-studied IgA-cleaving MPs around *Streptococcus* IgA1peptidase, which, like peptidases from other catalytic classes, specifically target P–S/T bonds. Uniquely, IgAse targets P–V bonds, enabling it to cleave both IgA1 and IgA2m(1), which provides a significant competitive advantage in the gut microbiome.

IgAse comprises seven domains centred around a novel CD ascribed to the igalysin family within the metzincin clan of MPs. High-resolution MX and SPA structures revealed that the downstream WD closely envelops the CD and is essential for its proper folding and function. The upstream NTD is a novel CLTD protein and downstream Oβ domain is an open-face β-sandwich. They both likewise contact the CD but less intimately, so they are not required for substrate cleavage, although Oβ accounts for higher thermal stability. The additional C-terminal domains ZBD1, BB8 and ZBD2, connected by flexible linkers as found by SEC–SAXS in solution, are also dispensable for catalysis and even hamper IgA cleavage *in vitro*.

The only targets of IgAse are IgA1 and IgA2, and no specific inhibitors were found beyond general zinc chelators. This aligns with the absence of potent inhibitors specific to any IgA-cleaving MP to date [5]. The minimal substrate can be narrowed down to the F_ab_’ fragment (construct IgA2Δ3), which still allows for cleavage. This is remarkable given that the *Steptococcus* and *Gemella* M26-family IgA1-MPs require interaction with the complete F_c_ fragment for cleavage [16,18]. An AI-based prediction model of IgA2Δ3 in complex with a construct spanning the experimentally determined NTD, CD, WD and Oβ domains of IgAse suggests that the primary substrate–enzyme interaction likely occurs between the C_H_1 domain of the HC and the USD of the CD, along with the top flap of the WD (Fig 4G). This positioning would place the scissile segment of the F_ab_’ downstream of C_H_1 close to the active-site cleft. To validate this hypothesis, we searched for residues on the surface of C_H_1 that are not involved in interactions with other IgA-domains but should disturb the predicted interface with IgAse, and designed a double and a triple point mutant (T^84^R/T^98^W and S^94^Y/T^98^W/D^96^R). We cloned, expressed, and purified these mutants similarly to wild-type IgA2Δ3, and found that processing by IgAse was indeed ablated in both cases (S10 Fig).

Overall, the present results pave the way for therapeutic strategies aimed at inhibiting the only known IgA1/IgA2 peptidase for antimicrobial therapy, as well as for developing methods to dissolve aberrant IgA deposits associated with IgA-related pathologies. Of note, recent studies have revealed reduction of IgA deposits upon treatment with IgAse in mice, highlighting its potential therapeutic application in IgA nephropathy [57].

## 3. Material and methods

***3.1. Biocomputational methods —*** The three-dimensional structure of the complete IgAse protein (M^1^–E^1234^) was predicted using *AlphaFold3* [24]. This approach employed paired multiple sequence alignments to extract coevolutionary data, enhancing the accuracy of the prediction [58]. The confidence levels of such AI-derived structure predictions are evaluated using the pLDDT [59], a robust method for assessing the Cα local-distance difference test accuracy of deep learning-based models [60]. In this context, average pLDDT values >90 indicate highly accurate predictions, values between 90 and 70 account for generally correct backbone predictions, and values below 70 reflect low-confidence predictions or disordered regions [61]. The resulting models were visually inspected and punctually corrected for chemical and geometrical validity and logical consistency using the *Coot* program [62]. Interfaces between domains, calculated as half the sum of the buried surface areas of each domain, were evaluated with the *Pisa* software [63]. Structural superpositions were performed using the *Ssm* routine [64] in *Coot*. Related structures available in the PDB were identified using *Dali* [28], and structural visualizations were generated with *Chimera* [65] and *ChimeraX* [66], as well as commercial office productivity software.

***3.2. Expression and purification of recombinant IgAse*** — The full-length *T. ramosa* IgAse coding gene, inserted into the pGEX-6p1 vector, was generously provided by Jan Potempa from Kraków, Poland, and Louisville, USA. Constructs were designed for domains NTD (IgAse**1**; residues A^31^–E^319^), NTD + CD (IgAse**1–2**; A^31^–V^631^), NTD + CD + WD (IgAse**1–3**; A^31^–Q^807^), NTD + CD + WD + Oβ (IgAse**1–4**; A^31^–N^876^), NTD + CD + WD + Oβ + ZBD1 + BB8 + ZBD2 (IgAse**1–7**; A^31^–I^1166^), CD (IgAse**2**; S^328^–V^631^), CD + WD + Oβ (IgAse**2–4**; S^328^–N^876^), CD + WD + Oβ + ZBD1 (IgAse**2–5**; S^328^–T^1006^), ZBD1 (IgAse**5**; K^877^–T^1006^), ZBD1 + BB8 + ZBD2 (IgAse**5–7**; K^877^–T^1171^), BB8 (IgAse**6**; G^1007^–T^1097^), and ZBD2 (IgAse**7**; T^1100^–T^1171^) (see Fig 1C). These constructs were amplified using primers incorporating *NdeI* (forward) and *XhoI* (reverse) restriction sites (see S5 Table). The amplified products were inserted into the pCri7b vector [67], which added an N-terminal methionine and a C-terminal His₆-tag, via these restriction sites. Inactive variants of CD-containing constructs, in which the catalytic glutamate was replaced with alanine (E^540^A), were generated via PCR using the forward primer 5’-GCCTTTGGTCACGGCCTGCTGGG-3’ and reverse primer 5’-GTGCGCGAAGGTTTTGCTCGC-3’. The resulting plasmids were introduced into *E. coli* BL21 (DE3) cells (Novagen) and cultivated at 37 °C in Luria–Bertani medium supplemented with 30 μg/mL kanamycin. Expression was induced at an *OD*₆₀₀ of 0.8–1.2 with 0.5 mM isopropyl-β-*D*-thiogalactopyranoside, following a cooling period of 15–30 minutes in a cold room. Induced cultures were incubated overnight at 20 °C with shaking.

Cells were harvested by centrifugation at 5,000 × *g* for 20 min at 4 °C, and the pellet was resuspended in 50 mM Tris–HCl (pH 8.0), 300 mM sodium chloride, 10 mM imidazole, 10 mM calcium chloride, and 5 mM β-ME, supplemented with tablets of cOmplete EDTA-free inhibitor cocktail and DNase I (both from Roche Diagnostics). Lysis was carried out using a Constant Systems cell disruptor at 1.35 kbar, and the lysate was clarified by two rounds of centrifugation at 20,000 × *g* for 30 min at 4 °C. The supernatant was applied to a 1-mL Ni–NTA HisPur Resin column (Thermo Fisher Scientific) for IMAC. The bound protein was washed twice with 20 mL of 50 mM Tris–HCl (pH 8.0), 300 mM sodium chloride, 10 mM imidazole, 10 mM calcium chloride, and 5 mM β-ME, followed by a two-step elution using 5 mL of 50 mM Tris–HCl (pH 8.0), 150 mM sodium chloride, 250 mM imidazole, 10 mM calcium chloride, and 5 mM β-ME. To achieve further purification, the protein was subjected to SEC using a Superdex 200 10/300 GL column (Cytiva) equilibrated with 50 mM Tris–HCl (pH 8.0) and 150 mM sodium chloride. Fractions containing the purified protein were collected, pooled, and concentrated using Vivaspin ultrafiltration devices with a molecular weight cut-off of 10–30 kDa (Sartorius). The membranes were maintained in a storage buffer containing glycerol and sodium azide. Protein samples were aliquoted and flash-frozen in liquid nitrogen for subsequent assays. A summary of the viability of the distinct constructs is provided in S1 Table.

***3.3. Expression and purification of recombinant IgAs*** — Plasmids from Addgene (www.addgene.org) encoding full-length IgA1 and IgA2m(1), with the variable regions recognising either HER2/neu receptor (pVITRO1-Trastuzumab-IgA1/κ and pVITRO1-Trastuzumab-IgA2/κ [33]) or the grass pollen allergen Phl p 7 (pVITRO1-102.1F10-IgA1/λ and pVITRO1-102.1F10-IgA2/λ [33]), were used directly or to amplify specific LC and HC regions by PCR. For this purpose, forward primers containing recognition sequences for *Sma*I (for HC) or *Eco91*I (for LC) and reverse primers with an *Sfa*AI site (see S5 Table) were employed to introduce the constructs into a modified pCMV-SPORT6 vector (Thermo Fisher Scientific) for expression in human cells. Full-length HC constructs were obtained for both isotypes, and for IgA2m(1), further variants lacking the tailpiece (IgA2Δ1), the tailpiece and C_H_3 domain (IgA2Δ2), and the latter two plus C_H_2 (IgA2Δ3) were prepared. For the latter construct, also variants T^84^R/T^98^W and S^94^Y/T^98^W/D^96^R were obtained. In all cases, the original signal peptides were retained (via *Sma*I), and the stop codons were removed to enable translation of the C-terminal His_6_-tag from the expression vector for IMAC. For the LCs, the original signal peptides were replaced with the Igκ leader signal peptide from the pCMV-SPORT6 vector (via *Eco91*I), while the stop codons were preserved.

Human Expi293-F cells (Thermo Fisher Scientific), adapted to FreeStyle F17 expression medium (Gibco) [68], were cultured at 37 °C in a Multitron incubator shaker (Infors HT) at 150 rpm in a humidified atmosphere with 8% CO₂. The cells were maintained at a density below 3–5 × 10⁶ cells/mL, and sub-cultured every 3–4 days by dilution to 0.3–0.5 × 10⁶ cells/mL. For transfection, 1 mg of the original pVITRO plasmids (full-length constructs) was prepared in 20 mL of Opti-MEM medium (Gibco) with 3 mg of linear 25-kDa polyethyleneimine (Polysciences Europe) per liter of expression medium and incubated at room temperature for 15–20 minutes. Alternatively, 1.5 mg and 1.0 mg of LC- and HC-coding pCMV-SPORT6 vectors were combined. The cells, at a density of 1 × 10⁶ cells/mL, were then transfected with these mixtures and incubated for 3 days. The cell-culture supernatant was harvested, cleared by centrifugation at 3,800 × *g* for 20 min at 4 ºC, and filtered through a 0.45-μm cellulose acetate filter (Millipore). Standard expression volumes were 30 mL (in 125-mL flasks) or 250 mL (in 1-L flasks) for small- and large-scale expressions, respectively.

For affinity purification, full-length IgA1 samples were incubated overnight with Jacalin-resin (G Biosciences), which features the tetrameric two-chain plant-based lectin jacalin that binds to the *O*-glycans of the hinge region (Fig 1B). In contrast, IgA2m(2) and IgA2Δ1 were incubated with Peptide-M agarose (InvivoGen). The later displays a 50-residue synthetic peptide derived from streptococcal protein M, which specifically recognizes the linker region between C_H_2 and C_H_3 of the F_c_ antibody fragment [69] that requires the F_c_ region to be properly folded. In either case, resins were washed with 10 column volumes (CV) of 50 mM Hepes (pH 7.5) and 300 mM sodium chloride. Proteins were then eluted in five steps of 2 CV of either 100 mM *D*-glucose in 50 mM Hepes (pH 7.5) and 10 mM sodium chloride or 100 mM glycine (pH 3.0) and 150 mM sodium chloride, respectively. For IgA2Δ2 and the IgA2Δ3 variants, the supernatants where supplemented with 50 mM Tris–HCl (pH 8.0) and incubated overnight with cOmplete His-tag purification resin (Roche). The bound samples were washed with two steps of 10 CV of 50 mM Tris–HCl (pH 8.0), 300 mM sodium chloride and 5 mM imidazole. Constructs were eluted with five steps of 2 CV of 50 mM Tris–HCl (pH 8.0), 150 mM sodium chloride and 50 mM imidazole. Of note, 100 µL or 500 µL resin was used in gravity columns for small- and large-scale expressions, respectively. Finally, purified IgA from human colostrum and plasma, containing a mixture of IgA1 and IgA2, were purchased from Sigma-Aldrich.

***3.4. Proteolytic activity and inhibition assays —*** The proteolytic activity of IgAse**1–3**, IgAse**1–4**, IgAse**2–4**, and IgAse**1–7** was assessed against IgA from human plasma and colostrum by incubating 4 µg and 6 µg of substrate, respectively, overnight at 37 ºC with 0.4 µg of each IgAse variant in a reaction volume of 40 µL containing 50 mM Tris–HCl (pH 8.0) and 150 mM sodium chloride. The reaction was stopped by adding 10 µL of 5 × Laemmli sample buffer (Sigma). Samples were heated at 95 ºC for 5 minutes, and 25 µL of each sample, containing 0.2 µg of IgAse and 2 µg of plasma IgA or 3 µg of colostrum IgA, was subjected to SDS-PAGE. Inactive E^540^A-mutants and wild-type peptidase constructs pre-incubated with 10 mM EDTA were analysed in the same manner. For time-resolved assays, plasma IgA (2 µM) was incubated at 37 ºC with the above active IgAse constructs at 0.1 µM (20:1 molar ratio) in a final volume of 150 µL of 50 mM Tris–HCl (pH 8.0), 150 mM sodium chloride, and 50 µM zinc chloride. Aliquots of 20 µL were withdrawn at 15, 30, 60, 120, and 240 min, stopped with 5 µL of 5 × Laemmli sample buffer as described above, and analysed by SDS-PAGE. Percentage of cleavage was determined by quantifying the band intensities of the C_H_3–C_H_2 cleavage fragment compared with the intact HC using the *ImageJ* program [70] and deriving the corresponding intensity of the V_H_–C_H_1 fragment from the C_H_3–C_H_2 measurement. To normalize for sample loading, the cleavage percentage was then determined by dividing the combined intensity of the cleavage products by the total intensity of the three bands. Subsequently, 38 µL of recombinant IgA1, IgA2, IgA2Δ1, IgA2Δ3, and the IgA2Δ3 variants (all at 10–100 ng/µL) were incubated overnight at 37 ºC in 50 mM Tris–HCl (pH 8.0), 150 mM sodium chloride, and 50 mM imidazole, with 2 µL of IgAse**1–4** and IgAse**1–4** + E^540^A at concentrations of 0.2 mg/mL (full-length IgAs) or 0.4 mg/mL (truncated IgA2 variants). Reactions were stopped and analysed by SDS-PAGE as for purified IgA.

To assess the peptide cleavage capacity of IgAse, we utilized the C-terminally amidated synthetic peptide VTVPSPVPSTPAR-NH_2_ (monoisotopic mass: 1306.7 [M + H]+). This peptide was derived from the V^97^–P^107^ fragment of the IgA1 hinge region (UP P01876), which was modified by introducing the C^101^S mutation, along with the addition of an alanine and an arginine at the C-terminus to enhance peptide solubility and detection by mass spectrometry [71]. The peptide was dissolved in HPLC-grade water to a final concentration of 10 mg/mL. A 1-μL aliquot of this peptide solution (10 μg) was incubated overnight at 37 ºC with 9 μl IgAse**1–4** (at 1 mg/mL; molar ratio ~80:1) in a buffer containing 50 mM Tris–HCl (pH 8.0) and 150 mM sodium chloride. Following incubation, the samples were desalted using C18 ZipTips (Merck) and eluted with 10 μL. A 0.5-μL aliquot was then spotted onto a MALDI target plate (Bruker). After mixing the sample with an equal volume of 2,4-dihydroxybenzoic acid matrix, ionization products were analysed using an AutoFLEX III MALDI-TOF mass spectrometer (Bruker) in linear-mode geometry. Control spectra were also recorded for the peptide and peptidase alone.

Finally, for inhibition assays, specific and non-specific MP inhibitors—batimastat, captopril, GM1489, MMPI 5, phosphoramidon, EDTA, and *o*-phenanthroline—were pre-incubated at a concentration of 1 mM (10 mM for EDTA) with 0.4 µg of IgAse**2–4** in 40 µL of 50 mM Tris–HCl (pH 8.0) and 150 mM sodium chloride for 15 minutes. Thereafter, 4 µg of plasma IgA was added to the reaction mixtures, which was then incubated at 37 ºC for 2 hours, stopped as previously described, and analysed by SDS-PAGE.

***3.5. Thermal-shift assays —*** The thermal stability of IgAse**1–3**, IgAse**1–4**, and IgAse**1–7** was assessed using differential scanning fluorimetry following the thermofluor method [31] with an iCycler iQ5 multicolor real-time PCR detection system (Bio-Rad) and high-profile, semi-skirted black/white Hard-Shell 96-well PCR plates (Bio-Rad), sealed with optical-quality Microseal ‘B’ adhesive film (Bio-Rad). Samples were prepared by combining 2 µL of 62 × SYPRO Orange protein stain (Invitrogen) and 2 µL IgAse (at 1 mg/mL in 50 mM Tris–HCl (pH 8.0) and 150 mM sodium chloride) with 21 µL of SPG buffer (12.5 mM succinic acid, 43.8 mM sodium dihydrogen phosphate monohydrate, and 43.7 mM glycine). The SPG buffer also contained 150 mM sodium chloride and had been adjusted to pH values of 4.5, 5.5, 6.5 and 7.5. Melting curves were recorded with an excitation wavelength (λ_ex_) of 450–490 nm and an emission wavelength filter (λ_em_) of 515–530 nm, and analysed with the *Tsa-Craft* program [72] to determine the *T*_m_, which represents the midpoint of the thermal denaturation curve.

***3.6. Small-angle X-ray scattering analysis*** — SAXS data of IgAse**1–7** directly after SEC was recorded at the BioSAXS beamline BM29 of the ESRF Synchrotron. A 40-µL protein sample (at 4 mg/mL) in 50 mM Tris–HCl (pH 8.0) and 150 mM sodium chloride was injected into a thoroughly pre-equilibrated Superose 6 Increase 50/150 GL column, coupled to an X-ray source and a Pilatus3 2M pixel detector (Dectris), for data collection at 293 K. Data reduction and processing was performed using the *FreeSAS* program by J. Kieffer (https://www.esrf.fr/files/live/sites/www/files/UsersAndScience/Experiments/MX/BAG%20Meeting/BAG%20meeting%202021/PP_UM2021.pdf), the *Chromixs* program [73] and the *BioXTAS Raw* suite (https://bioxtas-raw.readthedocs.io/en/latest). The latter incorporated the *Gnom* program [74] to calculate scattering profiles, perform averaging and buffer subtraction, analyse *R*_g_ via the Guinier approximation, and determine the molecular mass. Ensemble generation and fitting were conducted using the *Eom* procedure [75] from the *Atsas* suite of programs [76]. Hinge regions were identified based on low pLDDT scores (<70) of the AI-based predicted model (see Section 3.1) and visual inspection. S4 Table provides a summary of SEC–SAXS data collection and processing statistics.

***3.7. Crystallization and X-ray diffraction data collection*** — Constructs IgAse**1**, IgAse**1–3**, IgAse**1–4**, IgAse**1–7**, IgAse**2–4**, IgAse**2–5**, and IgAse**7**, both as wild-type forms and E^540^A-mutants for constructs encompassing the CD, were subjected to crystallization assays using the sitting-drop vapour diffusion method. Reservoir solutions were prepared in 96-deep-well blocks of 2-mL capacity with a Tecan robot (Tecan Life Sciences). A Phoenix robot (Art Robbins) dispensed 100 nL of protein solution at 5–10 mg/mL in 50 mM Tris–HCl (pH 8.0) and 150 mM sodium chloride, along with 100 nL of reservoir solution, into 96-well, two-drop Swissci PS MRC plates (Molecular Dimensions). Over 1,500 conditions from various crystallization screens from Hampton Research and Molecular Dimensions were tested for each protein construct at the in-house Automated Crystallography Platform (https://www.ibmb.csic.es/en/platforms/automated-crystallographic-platform). Plates were incubated at either 4 °C or 20 °C in Bruker steady-temperature crystal farms, with remote access available for monitoring crystal growth. Only IgAse**2–4** and IgAse**2–4** + E^540^A crystallized, and the highest-quality diffraction was obtained from crystals of the latter variant grown in drops containing 20% polyethylene glycol (PEG) 3350 and 0.2 M sodium fluoride as the reservoir solution. Additional diffracting crystals were observed using 20% PEG 8000, 0.2 M sodium chloride and 0.1 M phosphate–citrate buffer (pH 4.2); 17% PEG 10,000, 0.1 M Bis-Tris and 0.1 M ammonium acetate (pH 5.5); and 20% PEG 8000 and 0.1 M Ches (pH 9.5) as reservoir solutions.

IgAse**2–4** + E^540^A crystals were cryoprotected by adding reservoir solution supplemented with ethylene glycol to a final concentration of 15% (v/v) before being flash-vitrified in liquid nitrogen. Samples were then transported in a cryogenic dewar to the ALBA Synchrotron. Diffraction data were collected to 1.75 Å resolution from a crystal cryocooled at -173 °C, using a Pilatus3 X 6M detector at beamline BL13-XALOC [77] on 19 April 2023. The energy used for data collection was 12.662 keV, with a flux of 1.63 × 10¹² photons/s. Diffraction data were indexed, integrated, and merged using *Xds* [78] and *Xscale* [79]. The dataset was subsequently converted to MTZ format using *Xdsconv* for the selection of test-set reflections to monitor the free R_factor_, and subsequent structure determination and refinement. A summary of the data processing statistics is provided in S2 Table.

***3.8. Crystal structure solution and refinement*** — The crystal structure of IgAse**2–4** + E^540^A was solved by molecular replacement using the *Phaser* program [80]. Two search models derived from the prediction in Section 3.1 were used, corresponding to domains CD + WD (N^331^–N^807^) and Oβ (R^808^–N^876^), respectively. These calculations, which identified P2_1_ as the correct space group, yielded a unique solution for either moiety, corresponding to a single IgAse protomer in the asymmetric unit (a.u.). The solutions were determined at Eulerian angles 34.562, 22.123, 323.573 (rotation-function Z-score [RFZ]=7.7) and 30.205, 22.839, 327.939 (RFZ = 4.8), respectively. The corresponding translation values in fractional cell coordinates were 0.4777, -0.0011, 0.1669 (translation-function Z-score after final rotation-function refinement [TFZ]=8.6) and 0.4747, -0.0017, 0.1633 (TFZ = 27.0), respectively. This two-body search accounted for the difference in the relative orientations of CD + WD and Oβ observed between the predicted and actual structures. The final log-likelihood gain was 1506, and there was only one packing clash. The rotated and translated search models were used to compute phases for automatic density interpretation with *Arp*/*wArp* [81], resulting in an improved σ_A_-weighted (2m*F*_obs_–D*F*_calc_)-type Fourier map and an initial set of coordinates. This structure was manually rebuilt using *Coot* and subjected to crystallographic refinement with *Buster/TNT* [82] and the *Refine* protocol of the *Phenix* suite [83], iteratively until completion. Both refinement strategies included TLS refinement and occupancy refinement of residues with alternative conformations. The final structure encompasses IgAse residues E^330^–N^876^, plus a leucine from the C-terminal purification tag, two zinc cations, a tetraethylene glycol molecule, eight ethylene glycol molecules, and 430 solvent molecules. In addition, a formate anion, which is a metabolite of the *E. coli* expression host [84] potentially taken up by IgAse during heterologous overexpression, was tentatively assigned attached to the catalytic zinc (Fig 3B). S2 Table summarizes the structure refinement and validation statistics.

***3.9. Single particle cryo-electron microscopy structure solution and refinement*** — A frozen aliquot of IgAse**1–7** + E^540^A was thawed and injected onto a Superdex 200 5/150 column (Cytiva), pre-equilibrated with 50 mM Tris–HCl (pH 8.0) and 150 mM sodium chloride. A single peak fraction, with a protein concentration of 3.7 μM (0.5 mg/mL), was collected, and a 3-μL aliquot was applied to CF-1.2/1.3 grids (C-flat Holey Carbon Films) that had been glow-discharged for 60 seconds at 15 mA. The grids were plunge-frozen in liquid ethane using a Vitrobot Mark IV (Thermo Fisher Scientific) under 95% humidity at 4 °C, with a blotting force of −6 and a blot time of 4–6 seconds. Movies were acquired using the semi-automated *Epu 3* software (Thermo Fisher Scientific) on a 200-kV Glacios extreme field-emission gun microscope, equipped with a Falcon 4 direct electron detector (Thermo Fisher Scientific). This data collection was conducted at the IBMB-CSIC Cryo-Electron Microscopy Platform, which is part of the Joint Electron Microscopy Centre (JEMCA) at beamline EM01-CRYO-TEM of the ALBA Synchrotron (https://ibmb.csic.es/en/platforms/cryo-electron-microscope). Additional high-resolution data were obtained using a 300-kV Krios G4 microscope (Thermo Fisher Scientific), equipped with a cold field emission gun, a Selectris X energy filter, and a Falcon 4i direct electron detector, at the French CRG beamline CM02 located at the ESRF Synchrotron. For the Glacios dataset, images were recorded in electron-counting mode at 150,000 ×  magnification, with a calibrated pixel size of 0.93 Å, and a defocus range of -1.5 to -2.5 μm in 0.2-μm increments. For the Krios dataset, images were acquired in electron-counting mode at 165,000 ×  magnification, with a calibrated pixel size of 0.73 Å, and a defocus range of −0.8 to −2.0 μm in 0.2 μm increments. A summary of the data collection parameters is provided in S3 Table.

The Krios dataset was processed using *CryoSPARC* [85] with patch motion correction to estimate and correct for both full-frame motion and local sample deformation. The patch contrast transfer function (CTF) estimation was applied to fit local CTF parameters to the micrographs. Low-quality micrographs and outliers were manually removed during curation, which involved excluding those with relative ice thickness values greater than 1.2 or CTF fits worse than 10 Å. Initial particle picking was performed on a subset of 500 micrographs using template-based picking, with a 15-Å resolution volume derived from the IgAse**2–4** X-ray structure as the template. Particles were extracted with a box size of 320 pixels, followed by a first round of 2D classification to select the best classes for a second round of template picking on the curated micrographs. Subsequent processing included a further 2D classification, followed by *ab initio* reconstruction into five classes. The best *ab initio* volume was subjected to homogeneous refinement, followed by non-uniform refinement. CTF refinement and local refinement were then applied to this refined reconstruction. A final round of reference-based motion correction was performed on the particles used to build the volume, followed by non-uniform refinement, with CTF refinement and local refinement enabled, resulting in the final raw map. Thereafter, the model-independent sharpened *DeepEMhancer* map [86] was computed by *CryoSPARC*. A summary of this data processing is provided in Fig 5A–C and S3 Table.

The Glacios dataset was processed similarly with *CryoSPARC*, except that templates obtained from the initial 2D classification were used to train *Topaz* [87] for particle picking on 500 micrographs. These micrographs underwent a further round of 2D classification, and the best classes were used to refine the *Topaz* model across the full dataset. Downstream processing proceeded as described above. A summary of this data processing is provided in S8 Fig and S3 Table. Of note, we initially obtained the 200-kV Glacios data, which yielded a medium/low-resolution volume, as a requisite for access to a 300-kV Krios machine.

A starting model, comprising the refined crystallographic structure of CD + WD + Oβ along with the NTD from the *AlphaFold3* prediction (Section 3.1), was positioned into the final raw map generated by *CryoSPARC* using rigid-body fitting in *Chimera*. As in our MX analysis, this two-body positioning accounted for the difference in relative orientations between the two components. The raw map and the *DeepEMhancer*-map were used for manual model rebuilding in *Coot*. The latter map showed substantially more contrast and structural detail, consistent with a nominal resolution of 2.8 Å, compared with the raw map, which, as is usual, lacked contrast at high resolution [86]. Subsequently, atomic positions—including solvent molecules—along with atomic displacement parameters and occupancies of residues modelled in alternate conformations were refined in an initial step using the *Real-Space-Refinement* protocol [88] in *Phenix* against the *DeepEMhancer* map. In a second step, atomic displacement parameters and alternate conformations were refined against the raw map to obtain unbiased statistics. This process was repeated iteratively until the final structure was obtained, which encompassed residues S^32^–N^876^, three zinc ions, as well as one glycerol and seven azide molecules from the concentrator buffer, and 151 solvent molecules. Residue C^251^ was covalently modified with β-ΜΕ. S3 Table summarizes the final model refinement and validation statistics against the raw map.

## Supporting information

S1 Fig Recombinant protein expression, purification, and crystallization of IgAse2–4. (A) Representative SDS-PAGE analysis illustrating protein expression, cell lysis, and IMAC purification. *Lanes*: 0h, pre-induction; ON, post-induction after overnight incubation at 20 °C; TL, total lysate; P, pellet; SN, soluble supernatant fraction; M, molecular mass marker; FT, flow-through; E1, elution 1 (250 mM imidazole); E2, elution 2 (250 mM imidazole). Of note, IgAse**2–4** exhibits an apparent molecular mass of ~63 kDa. (B) SEC profile of purified IgAse**2–4** + E^540^A analysed using a Superdex 200 10/300 GL column, showing a monodisperse peak at a retention volume of ~16.2 mL, which corresponds to a monomer. Fractions selected for crystallization are indicated by a slate blue bar at the peak base. (C) Reducing SDS-PAGE analysis of the SEC run from (B), with fractions selected for crystallization indicated by a slate blue bar. (D) Reducing SDS-PAGE analysis of both active IgAse**2–4** (HExxH) and inactive IgAse**2–4** + E^540^A (HAxxH) used for crystallization after protein concentration. On the *right*, representative images of diffracted crystals in their cryo-loops.
(DOCX)

S2 Fig Recombinant production and purification of IgAse1–7 + E540A for cryo-EM SPA. (A) Representative SDS-PAGE analysis illustrating protein expression, cell lysis, and IMAC purification. *Lanes*: 0h, pre-induction; ON, post-induction after overnight incubation at 20 °C; M, molecular mass marker; TL, total lysate; P, pellet; SN, soluble supernatant fraction; FT, flow-through; W1, wash (10 mM imidazole); W2, wash (10 mM imidazole); E1, elution 1 (250 mM imidazole); E2, elution 2 (250 mM imidazole). (B) SEC profile of IgAse**1–7** + E^540^A analysed using a Superdex 200 10/300 GL column, exhibiting a monodisperse peak at a retention volume of ~11.9 mL, which corresponds to a monomer of ~130 kDa. Fractions selected for concentration and freezing are indicated by a green bar at the peak base. (C) Reducing SDS-page gel analysis of the SEC run from (B), revealing a co-migrating band (~11 kDa) resulting from cleavage. (D) SEC profile of IgAse**1–7** in an analytical S200 5/150 GL column, showing a retention volume of 1.47 mL. The fraction selected for cryo-EM grid preparation is indicated by a green bar at the peak base. (E) Reducing SDS-PAGE analysis of (D), with fraction 13 (50 μL) used for cryo-EM grid preparation highlighted by a green bar. (F) Representative micrograph from the dataset collected using a 300-kV Krios cryo-TEM equipped with a Falcon 4i camera.
(DOCX)

S3 Fig Production and purification of ancillary IgAse domains. (A) SDS-PAGE analysis of constructs IgAse**1** (NTD alone), IgAse**2–5** (CD + WD + Oβ + ZBD1) and IgAse**7** (ZBD2 alone) produced for protein crystallography (see also S1 Table). (B) SEC analysis using a Superdex 200 10/300 GL column of the protein samples shown in (A).
(DOCX)

S4 Fig Effect of dithiothreitol (DTT) on IgAse1–7 polydispersity. Results of SEC using a S200 10/300 GL column in the presence or absence of the reducing agent. In the absence of DTT (grey), IgAse**1–7** elutes heterogeneously, with a broad peak at 10.8 mL. The addition of DTT to the running buffer (green) promotes a predominant monodisperse peak at 11.6 mL, indicative of a stable monomeric state under reducing conditions. Of note, including β-ME during cell lysis and IMAC eliminated the need for DTT in SEC to achieve a monodisperse peak.
(DOCX)

S5 Fig Differential scanning fluorimetry analysis. (A) Thermal stability comparison at pH 6.5 for IgAse**1–7** (green), IgAse**1–4** (blue), and IgAse**1–3** (purple), as shown in Fig 2D. Additional thermal denaturation curves at pH 4.5 (purple), 5.5 (green), 6.5 (blue), and 7.5 (yellow) are shown for IgAse**1–3 (B)**, IgAse**1–4** (C), and IgAse**1–7** (D), revealing a clear pH dependency.
(DOCX)

S6 Fig Time-dependent IgA cleavage by IgAse. Reducing SDS-PAGE analysis of human plasma IgA (2 µM) incubated with (+) IgAse**1–3** (A), IgAse**1–4** (B), IgAse**2–4** (C), and IgAse**1–7** (D) (all at 0.1 µM) at multiple time points. Cleavage at the HC hinge region results in a fragment indicated by a red arrow. Note that the right lane in (C) depicts IgAse**2–4** at 0.2 µM to demonstrate comigration with the intact IgA heavy chain. This suggests that the remaining band at 4 hours most likely represents IgAse**2–4** and not non-cleaved IgA. Finally, non-treated IgA incubated without IgAse (-) at 0 and 4 hours is shown in *lanes* 1 and 2 of (D).
(DOCX)

S7 Fig Transient enzyme–substrate complex and MALDI-TOF mass spectrometry. (A) Independent SEC runs in a Superdex 200 10/300 GL column of plasma IgA (blue curve), inactive IgAse**1–7** + E^540^A (green curve), and a preincubated mixture of both (purple curve). No stable complex was observed, indicating that the enzyme–substrate interaction is transient. (B) MALDI-TOF mass-spectrometry analysis demonstrating that IgAse**1–4** (alone; top panel in blue) cleaves a synthetic peptide mimicking the IgA1 hinge region (see Section 3.4) (alone; middle panel in purple; peak at m/z 1306.7 [M + H]+) after overnight incubation, but only very poorly (bottom panel in green). The reaction, which results in less than 1% substrate turnover, gives rise to peaks at 621.4 and 726.6 m/z, as evident from the m/z 580–760 inset in green. These correspond to the VTVPSP ([M + H + Na]+) and VPSTPAR ([M + H]+) cleavage products. All three insets are scaled to the same y-axis values for comparison.
(DOCX)

S8 Fig Cryo-EM data processing. (A) Flowchart depicting cryo-electron microscopy single-particle analysis and image processing of the medium/low-resolution movies from a 200-kV Glacios microscope. (B) Gold-standard (0.143) Fourier Shell Correlation (GSFSC) curves. Model fitting was performed using our X-ray structure (CD + WD + Oβ) and the NTD plus connecting linker as modelled by *AlphaFold3*.
(DOCX)

S9 Fig SEC-SAXS analysis of IgAse1–7 using the full data range. (A) Experimental SEC-SAXS profile of IgAse**1–7** (blue). (B) Normalized Kratky plot derived from the SAXS profile shown in (A). (C) P(r) distribution fit (red curve) to the experimental SAXS data (blue curve) (χ^2^ = 1.05). (D) P(r) distribution showing a maximum linear dimension (d_max_) of 278 Å.
(DOCX)

S10 Fig IgAse activity analysis against wild-type and mutant IgA2Δ3. Reducing SDS-PAGE analysis showing quantitative cleavage in the hinge region (*lane* 4, red asterisk) of C-terminally truncated wild-type (WT) IgA2Δ3 (*lane* 3) after overnight incubation with IgAse**1–4** (*lane* 1). In contrast, mutants T^84^R/S^94^Y (*lanes* 5 and 6) and D^96^R/S^94^Y/T^98^W (*lanes* 7 and 8) are not cleaved. *Lane* 2 depicts the BlueStar Plus Molecular Weight ladder.
(DOCX)

S1 Table IgAse constructs assayed. (PPTX)

S2 Table Crystallographic data. (PPTX)

S3 Table Cryo-electron microscopy single-particle analysis. (PPTX)

S4 Table SEC-SAXS data collection and derived parameters for IgAse1–7. (PPTX)

S5 Table Primers employed for cloning. (PPTX)

S1 File Validation Report PDB 9QA6. (PDF)

S2 File Validation Report PDB 9I4Z. (PDF)
