# Correction: Biochemical and structural characterization of the human gut microbiome metallopeptidase IgAse provides insight into its unique specificity for the F_ab_’ region of IgA1 and IgA2

**DOI:** 10.1371/journal.ppat.1013742

**Published:** 2025-12-04

**Authors:** Juan Sebastián Ramírez-Larrota, Pauline Juyoux, Pablo Guerra, Ulrich Eckhard, F. Xavier Gomis-Rüth

The residue number/ID is incorrect in S10 Fig. Please view the correct S10 Fig in the Supporting information.

The X-ray and Cryo-EM structures final validation reports are missing in S1 File and S2 File. Please view the correct S1 File and S2 File in the Supporting information.

S1 Fig to S9 Fig and S1 Table to S5 Table are not in PDF format. Please view the correct S1 Fig to S9 Fig and S1 Table to S5 Table files in the Supporting information.

## Supporting information

S1 File Validation Report PDB 9QA6. (PDF)

S2 File Validation Report PDB 9I4Z. (PDF)

S1 Fig Recombinant protein expression, purification, and crystallization of IgAse2–4. (PDF)

S2 Fig Recombinant production and purification of IgAse1–7 + E540A for cryo-EM SPA. (PDF)

S3 Fig Production and purification of ancillary IgAse domains. (PDF)

S4 Fig Effect of dithiothreitol (DTT) on IgAse1–7 polydispersity. (PDF)

S5 Fig Differential scanning fluorimetry analysis. (PDF)

S6 Fig Time-dependent IgA cleavage by IgAse. (PDF)

S7 Fig Transient enzyme–substrate complex and MALDI-TOF mass spectrometry. (PDF)

S8 Fig Cryo-EM data processing. (PDF)

S9 Fig SEC-SAXS analysis of IgAse1–7 using the full data range. (PDF)

S10 Fig IgAse activity analysis against wild-type and mutant IgA2Δ3. Reducing SDS-PAGE analysis showing quantitative cleavage in the hinge region (lane 4, red asterisk) of C-terminally truncated wild-type (WT) IgA2Δ3 (lane 3) after overnight incubation with IgAse1–4 (lane 1). In contrast, mutants T84R/S94Y (lanes 5 and 6) and D96R/S94Y/T98W (lanes 7 and 8) are not cleaved. Lane 2 depicts the BlueStar Plus Molecular Weight ladder.
(PDF)

S1 Table IgAse constructs assayed. (PDF)

S2 Table Crystallographic data. (PDF)

S3 Table Cryo-electron microscopy single-particle analysis. (PDF)

S4 Table SEC-SAXS data collection and derived parameters for IgAse1–7. (PDF)

S5 Table Primers employed for cloning. (PDF)
