# Supplementary material for: Biochemical and structural characterization of the human gut microbiome metallopeptidase IgAse provides insight into its unique specificity for the F ab ’ region of IgA1 and IgA2
Source: PLoS Pathog. 2025 Jul 8;21(7):e1013292. doi: 10.1371/journal.ppat.1013292 (PMC12237041; doi:10.1371/journal.ppat.1013292)
Supplement: S1 Fig — (A) Representative SDS-PAGE analysis illustrating protein expression, cell lysis, and IMAC purification. Lanes: 0h, pre-induction; ON, post-induction after overnight incubation at 20 °C; TL, total lysate; P, pellet; SN, soluble supernatant fraction; M, molecular mass marker; FT, flow-through; E1, elution 1 (250 mM imidazole); E2, elution 2 (250 mM imidazole). Of note, IgAse2–4 exhibits an apparent molecular mass of ~63 kDa. (B) SEC profile of purified IgAse2–4 + E540A analysed using a Superdex 200 10/300 GL column, showing a monodisperse peak at a retention volume of ~16.2 mL, which corresponds to a monomer. Fractions selected for crystallization are indicated by a slate blue bar at the peak base. (C) Reducing SDS-PAGE analysis of the SEC run from (B), with fractions selected for crystallization indicated by a slate blue bar. (D) Reducing SDS-PAGE analysis of both active IgAse2–4 (HExxH) and inactive IgAse2–4 + E540A (HAxxH) used for crystallization after protein concentration. On the right, representative images of diffracted crystals in their cryo-loops. (DOCX) [file ppat.1013292.s001.docx]

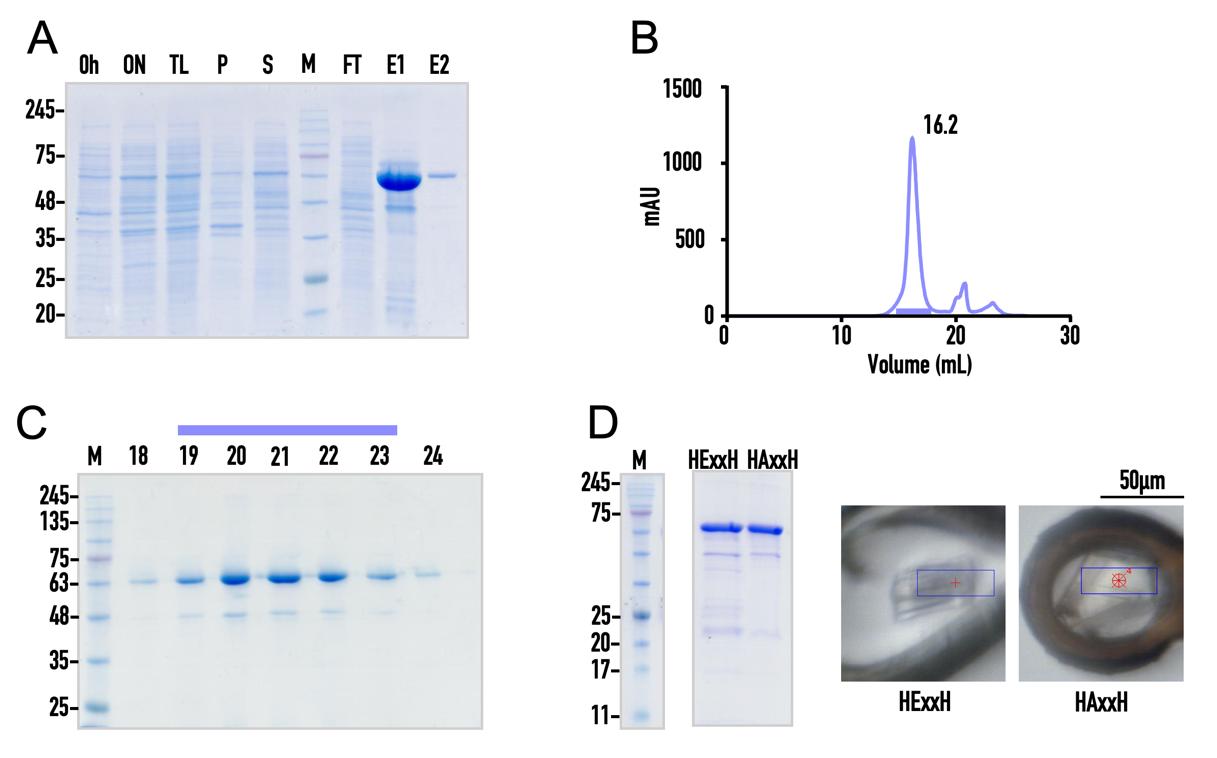


**S1 Fig — Recombinant protein expression, purification, and crystallization of IgAse2–4.** **(A)** Representative SDS-PAGE analysis illustrating protein expression, cell lysis, and IMAC purification. *Lanes*: 0h, pre-induction; ON, post-induction after overnight incubation at 20 °C; TL, total lysate; P, pellet; SN, soluble supernatant fraction; M, molecular mass marker; FT, flow-through; E1, elution 1 (250 mM imidazole); E2, elution 2 (250 mM imidazole). Of note, IgAse**2-4** exhibits an apparent molecular mass of ~63 kDa. **(B)** SEC profile of purified IgAse**2–4**+E^540^A analysed using a Superdex 200 10/300 GL column, showing a monodisperse peak at a retention volume of ~16.2 mL, which corresponds to a monomer. Fractions selected for crystallization are indicated by a slate blue bar at the peak base. **(C)** Reducing SDS-PAGE analysis of the SEC run from (B), with fractions selected for crystallization indicated by a slate blue bar. **(D)** Reducing SDS-PAGE analysis of both active IgAse**2–4** (HExxH) and inactive IgAse**2–4**+E^540^A (HAxxH) used for crystallization after protein concentration. On the *right*, representative images of diffracted crystals in their cryo-loops.
