# Supplementary material for: Biochemical and structural characterization of the human gut microbiome metallopeptidase IgAse provides insight into its unique specificity for the F ab ’ region of IgA1 and IgA2
Source: PLoS Pathog. 2025 Jul 8;21(7):e1013292. doi: 10.1371/journal.ppat.1013292 (PMC12237041; doi:10.1371/journal.ppat.1013292)
Supplement: S2 Fig — (A) Representative SDS-PAGE analysis illustrating protein expression, cell lysis, and IMAC purification. Lanes: 0h, pre-induction; ON, post-induction after overnight incubation at 20 °C; M, molecular mass marker; TL, total lysate; P, pellet; SN, soluble supernatant fraction; FT, flow-through; W1, wash (10 mM imidazole); W2, wash (10 mM imidazole); E1, elution 1 (250 mM imidazole); E2, elution 2 (250 mM imidazole). (B) SEC profile of IgAse1–7 + E540A analysed using a Superdex 200 10/300 GL column, exhibiting a monodisperse peak at a retention volume of ~11.9 mL, which corresponds to a monomer of ~130 kDa. Fractions selected for concentration and freezing are indicated by a green bar at the peak base. (C) Reducing SDS-page gel analysis of the SEC run from (B), revealing a co-migrating band (~11 kDa) resulting from cleavage. (D) SEC profile of IgAse1–7 in an analytical S200 5/150 GL column, showing a retention volume of 1.47 mL. The fraction selected for cryo-EM grid preparation is indicated by a green bar at the peak base. (E) Reducing SDS-PAGE analysis of (D), with fraction 13 (50 μL) used for cryo-EM grid preparation highlighted by a green bar. (F) Representative micrograph from the dataset collected using a 300-kV Krios cryo-TEM equipped with a Falcon 4i camera. (DOCX) [file ppat.1013292.s002.docx]

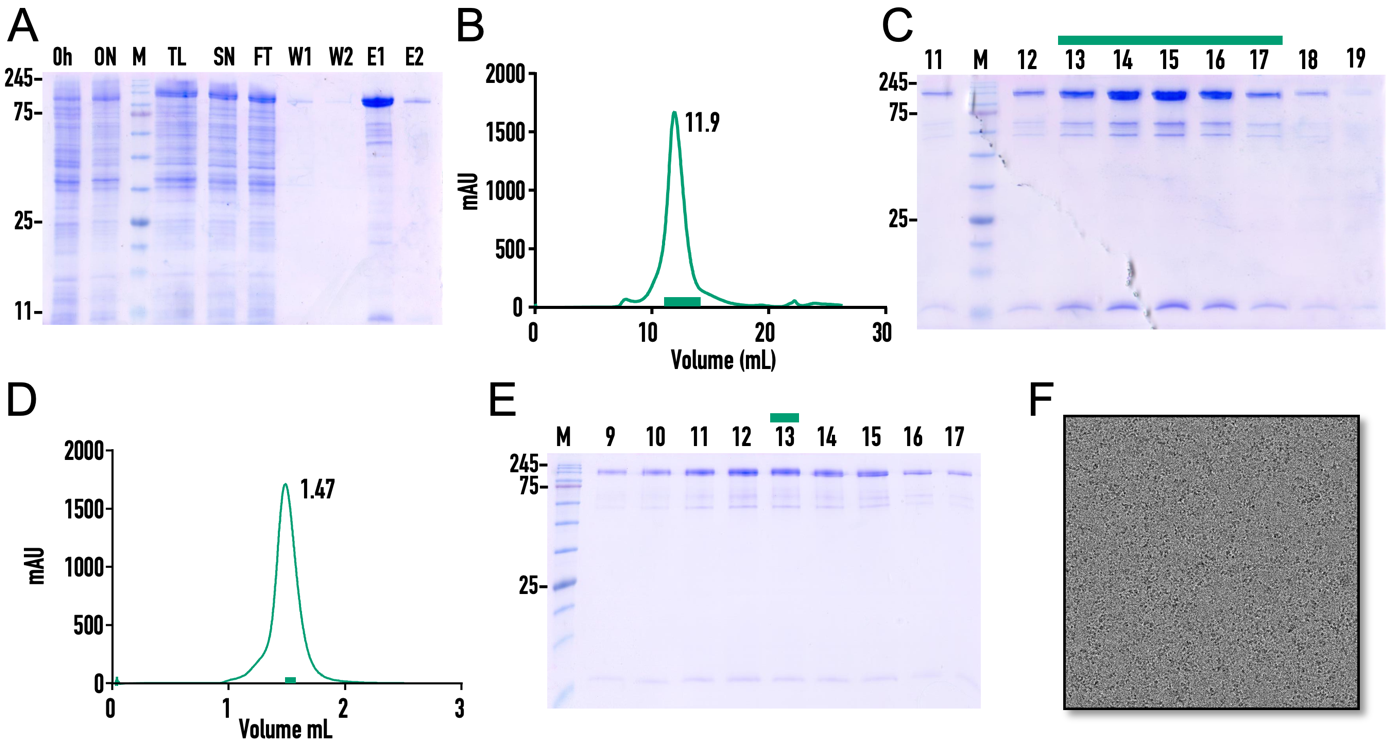


**S2 Fig — Recombinant production and purification of** **IgAse1–7+E^540^A for cryo-EM SPA**. **(A)** Representative SDS-PAGE analysis illustrating protein expression, cell lysis, and IMAC purification. *Lanes*: 0h, pre-induction; ON, post-induction after overnight incubation at 20 °C; M, molecular mass marker; TL, total lysate; P, pellet; SN, soluble supernatant fraction; FT, flow-through; W1, wash (10 mM imidazole); W2, wash (10 mM imidazole); E1, elution 1 (250 mM imidazole); E2, elution 2 (250 mM imidazole). **(B)** SEC profile of IgAse**1–7**+E^540^A analysed using a Superdex 200 10/300 GL column**,** exhibiting a monodisperse peak at a retention volume of ~11.9 mL, which corresponds to a monomer of ~130 kDa. Fractions selected for concentration and freezing are indicated by a green bar at the peak base. **(C)** Reducing SDS-page gel analysis of the SEC run from (B), revealing a co-migrating band (~11 kDa) resulting from cleavage. **(D)** SEC profile of IgAse**1–7** in an analytical S200 5/150 GL column, showing a retention volume of 1.47 mL. The fraction selected for cryo-EM grid preparation is indicated by a green bar at the peak base. **(E)** Reducing SDS-PAGE analysis of (D), with fraction 13 (50 μL) used for cryo-EM grid preparation highlighted by a green bar. **(F)** Representative micrograph from the dataset collected using a 300-kV Krios cryo-TEM equipped with a Falcon 4i camera.
