# Supplementary material for: Biochemical and structural characterization of the human gut microbiome metallopeptidase IgAse provides insight into its unique specificity for the F ab ’ region of IgA1 and IgA2
Source: PLoS Pathog. 2025 Jul 8;21(7):e1013292. doi: 10.1371/journal.ppat.1013292 (PMC12237041; doi:10.1371/journal.ppat.1013292)
Supplement: S3 Fig — (A) SDS-PAGE analysis of constructs IgAse1 (NTD alone), IgAse2–5 (CD + WD + Oβ + ZBD1) and IgAse7 (ZBD2 alone) produced for protein crystallography (see also S1 Table). (B) SEC analysis using a Superdex 200 10/300 GL column of the protein samples shown in (A). (DOCX) [file ppat.1013292.s003.docx]

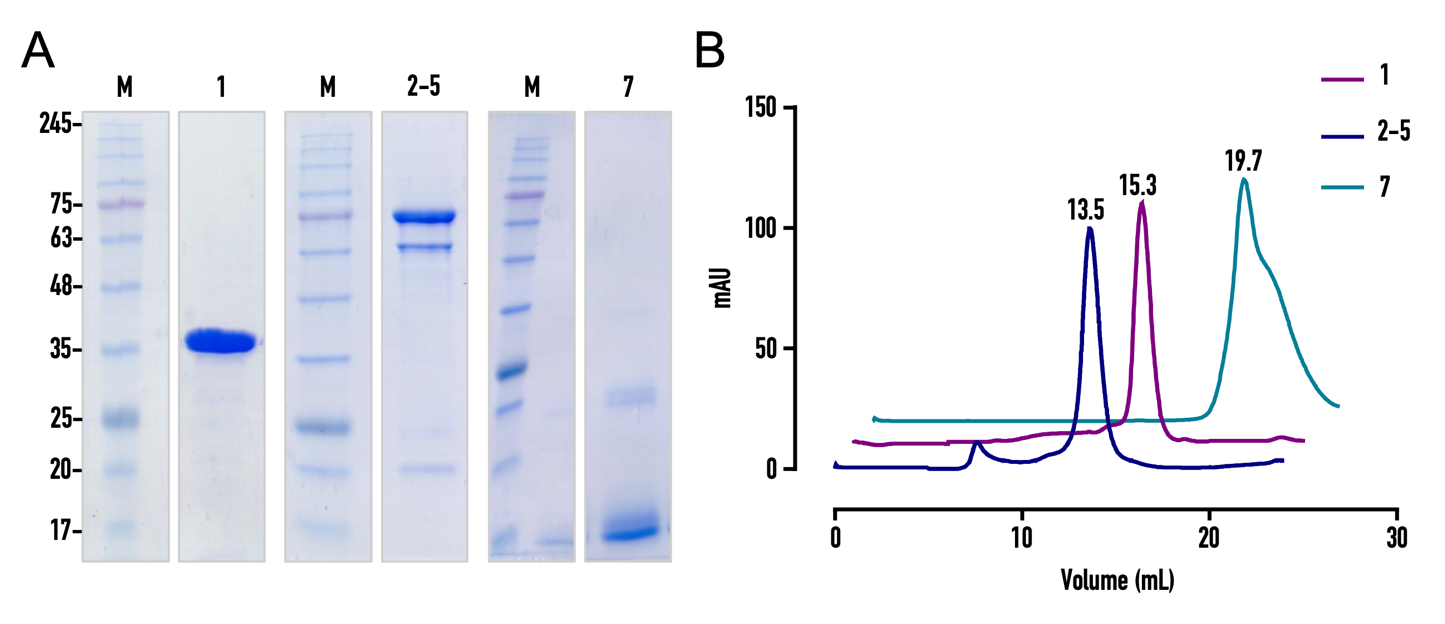


**S3 Fig — Production and purification of ancillary IgAse domains. (A)** SDS-PAGE analysis of constructs IgAse**1** (NTD alone), IgAse**2–5** (CD+WD+Oβ+ZBD1) and IgAse**7** (ZBD2 alone) produced for protein crystallography (see also Suppl. Table S1). **(B)** SEC analysis using a Superdex 200 10/300 GL column of the protein samples shown in (A).
