# Supplementary material for: Biochemical and structural characterization of the human gut microbiome metallopeptidase IgAse provides insight into its unique specificity for the F ab ’ region of IgA1 and IgA2
Source: PLoS Pathog. 2025 Jul 8;21(7):e1013292. doi: 10.1371/journal.ppat.1013292 (PMC12237041; doi:10.1371/journal.ppat.1013292)
Supplement: S4 Fig — Results of SEC using a S200 10/300 GL column in the presence or absence of the reducing agent. In the absence of DTT (grey), IgAse1–7 elutes heterogeneously, with a broad peak at 10.8 mL. The addition of DTT to the running buffer (green) promotes a predominant monodisperse peak at 11.6 mL, indicative of a stable monomeric state under reducing conditions. Of note, including β-ME during cell lysis and IMAC eliminated the need for DTT in SEC to achieve a monodisperse peak. (DOCX) [file ppat.1013292.s004.docx]

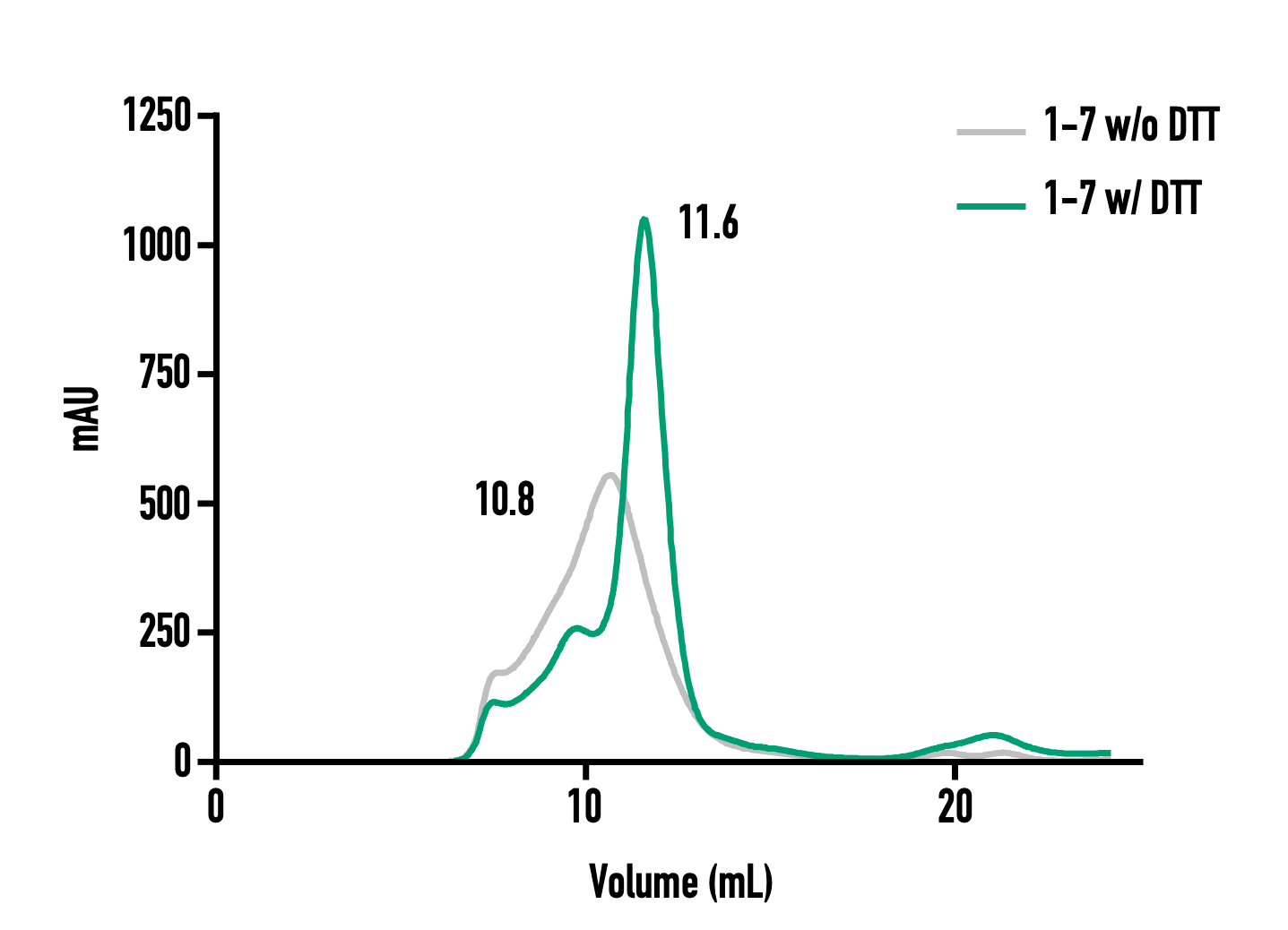


**S4 Fig — Effect of dithiothreitol (DTT) on IgAse1–7 polydispersity**. Results of SEC using a S200 10/300 GL column in the presence or absence of the reducing agent. In the absence of DTT (grey), IgAse**1–7** elutes heterogeneously, with a broad peak at 10.8 mL. The addition of DTT to the running buffer (green) promotes a predominant monodisperse peak at 11.6 mL, indicative of a stable monomeric state under reducing conditions. Of note, including β-ME during cell lysis and IMAC eliminated the need for DTT in SEC to achieve a monodisperse peak.
