# Supplementary material for: Biochemical and structural characterization of the human gut microbiome metallopeptidase IgAse provides insight into its unique specificity for the F ab ’ region of IgA1 and IgA2
Source: PLoS Pathog. 2025 Jul 8;21(7):e1013292. doi: 10.1371/journal.ppat.1013292 (PMC12237041; doi:10.1371/journal.ppat.1013292)
Supplement: S5 Fig — (A) Thermal stability comparison at pH 6.5 for IgAse1–7 (green), IgAse1–4 (blue), and IgAse1–3 (purple), as shown in Fig 2D. Additional thermal denaturation curves at pH 4.5 (purple), 5.5 (green), 6.5 (blue), and 7.5 (yellow) are shown for IgAse1–3 (B), IgAse1–4 (C), and IgAse1–7 (D), revealing a clear pH dependency. (DOCX) [file ppat.1013292.s005.docx]

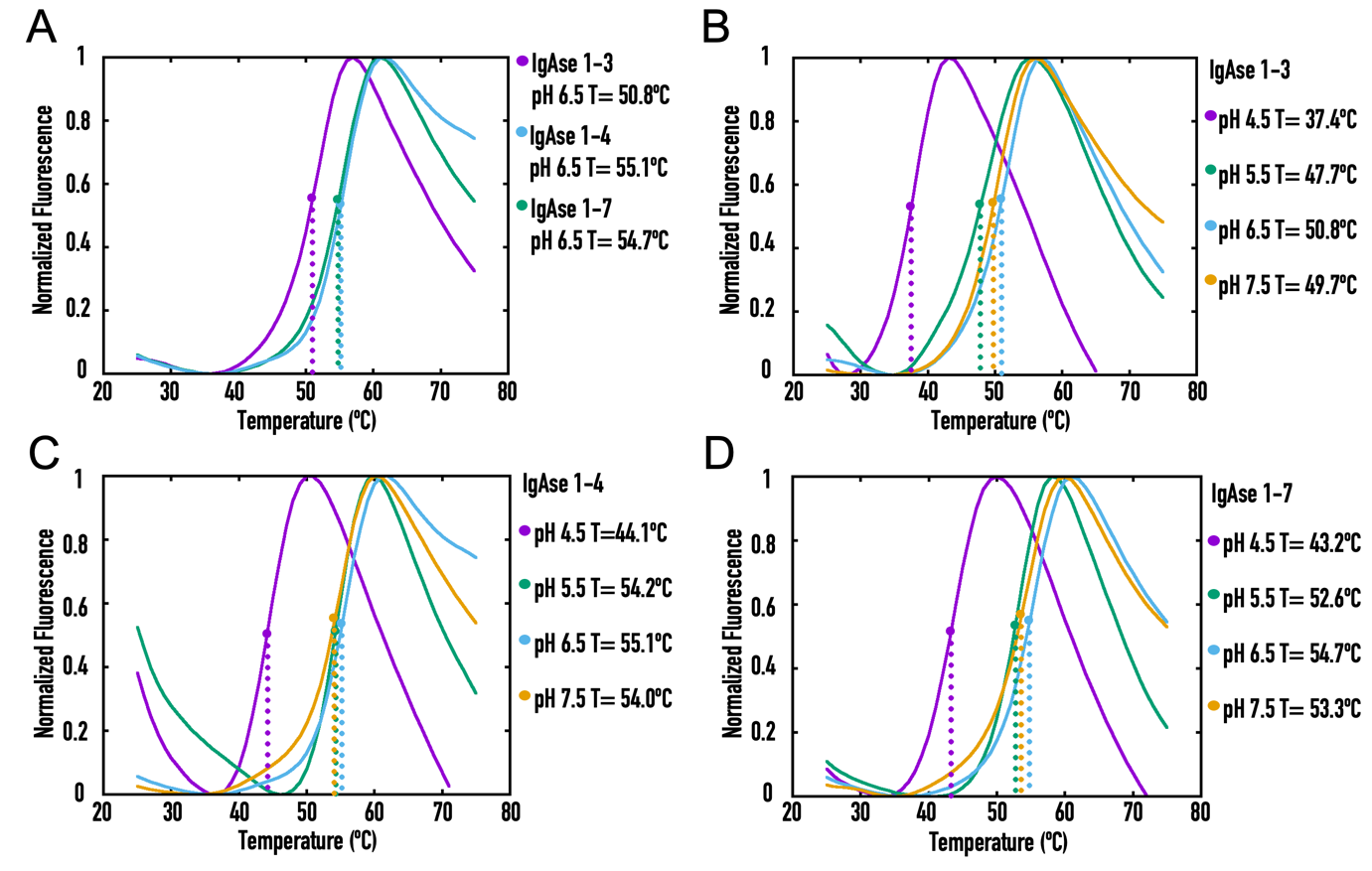


**S5 Fig — Differential scanning fluorimetry analysis**. **(A)** Thermal stability comparison at pH 6.5 for IgAse**1–7** (green), IgAse**1–4** (blue), and IgAse**1–3** (purple), as shown in Fig. 2D. Additional thermal denaturation curves at pH 4.5 (purple), 5.5 (green), 6.5 (blue), and 7.5 (yellow) are shown for IgAse**1–3** **(B)**, IgAse**1–4** **(C)**, and IgAse**1–7** **(D)**, revealing a clear pH dependency.
