# Supplementary material for: Biochemical and structural characterization of the human gut microbiome metallopeptidase IgAse provides insight into its unique specificity for the F ab ’ region of IgA1 and IgA2
Source: PLoS Pathog. 2025 Jul 8;21(7):e1013292. doi: 10.1371/journal.ppat.1013292 (PMC12237041; doi:10.1371/journal.ppat.1013292)
Supplement: S6 Fig — Reducing SDS-PAGE analysis of human plasma IgA (2 µM) incubated with (+) IgAse1–3 (A), IgAse1–4 (B), IgAse2–4 (C), and IgAse1–7 (D) (all at 0.1 µM) at multiple time points. Cleavage at the HC hinge region results in a fragment indicated by a red arrow. Note that the right lane in (C) depicts IgAse2–4 at 0.2 µM to demonstrate comigration with the intact IgA heavy chain. This suggests that the remaining band at 4 hours most likely represents IgAse2–4 and not non-cleaved IgA. Finally, non-treated IgA incubated without IgAse (-) at 0 and 4 hours is shown in lanes 1 and 2 of (D). (DOCX) [file ppat.1013292.s006.docx]

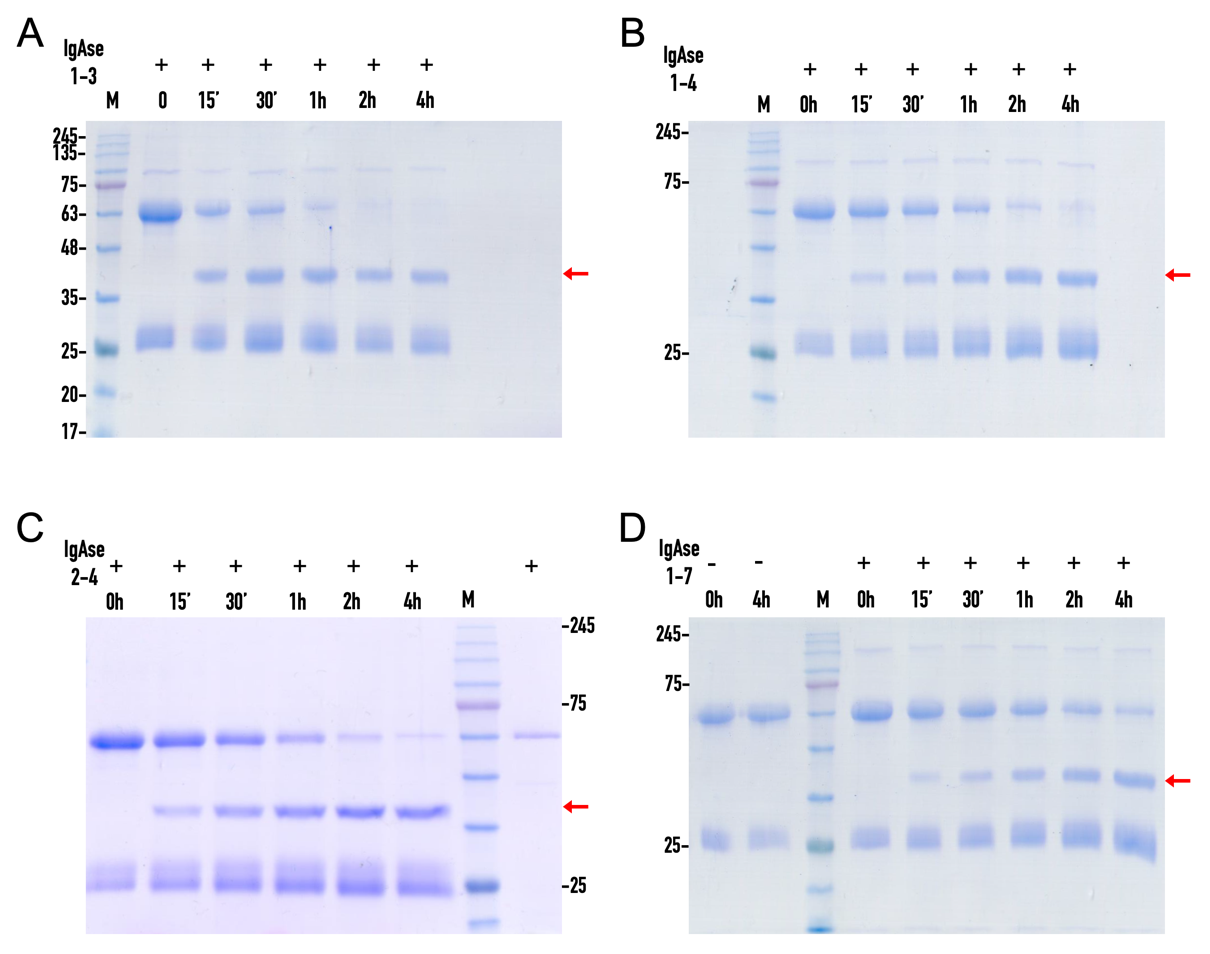


**S6 Fig — Time-dependent IgA cleavage by IgAse**. Reducing SDS-PAGE analysis of human plasma IgA (2 µM) incubated with (+) IgAse**1–3** **(A)**, IgAse**1–4** **(B)**, IgAse**2–4** **(C)**, and IgAse**1–7** **(D)** (all at 0.1 µM) at multiple time points. Cleavage at the HC hinge region results in a fragment indicated by a red arrow. Note that the right lane in **(C)** depicts IgAse**2–4** at 0.2 µM to demonstrate comigration with the intact IgA heavy chain. This suggests that the remaining band at 4 hours most likely represents IgAse**2–4** and not non-cleaved IgA. Finally, non-treated IgA incubated without IgAse (-) at 0 and 4 hours is shown in *lanes* 1 and 2 of **(D)**.
