# Supplementary material for: Biochemical and structural characterization of the human gut microbiome metallopeptidase IgAse provides insight into its unique specificity for the F ab ’ region of IgA1 and IgA2
Source: PLoS Pathog. 2025 Jul 8;21(7):e1013292. doi: 10.1371/journal.ppat.1013292 (PMC12237041; doi:10.1371/journal.ppat.1013292)
Supplement: S7 Fig — (A) Independent SEC runs in a Superdex 200 10/300 GL column of plasma IgA (blue curve), inactive IgAse1–7 + E540A (green curve), and a preincubated mixture of both (purple curve). No stable complex was observed, indicating that the enzyme–substrate interaction is transient. (B) MALDI-TOF mass-spectrometry analysis demonstrating that IgAse1–4 (alone; top panel in blue) cleaves a synthetic peptide mimicking the IgA1 hinge region (see Section 3.4) (alone; middle panel in purple; peak at m/z 1306.7 [M + H]+) after overnight incubation, but only very poorly (bottom panel in green). The reaction, which results in less than 1% substrate turnover, gives rise to peaks at 621.4 and 726.6 m/z, as evident from the m/z 580–760 inset in green. These correspond to the VTVPSP ([M + H + Na]+) and VPSTPAR ([M + H]+) cleavage products. All three insets are scaled to the same y-axis values for comparison. (DOCX) [file ppat.1013292.s007.docx]

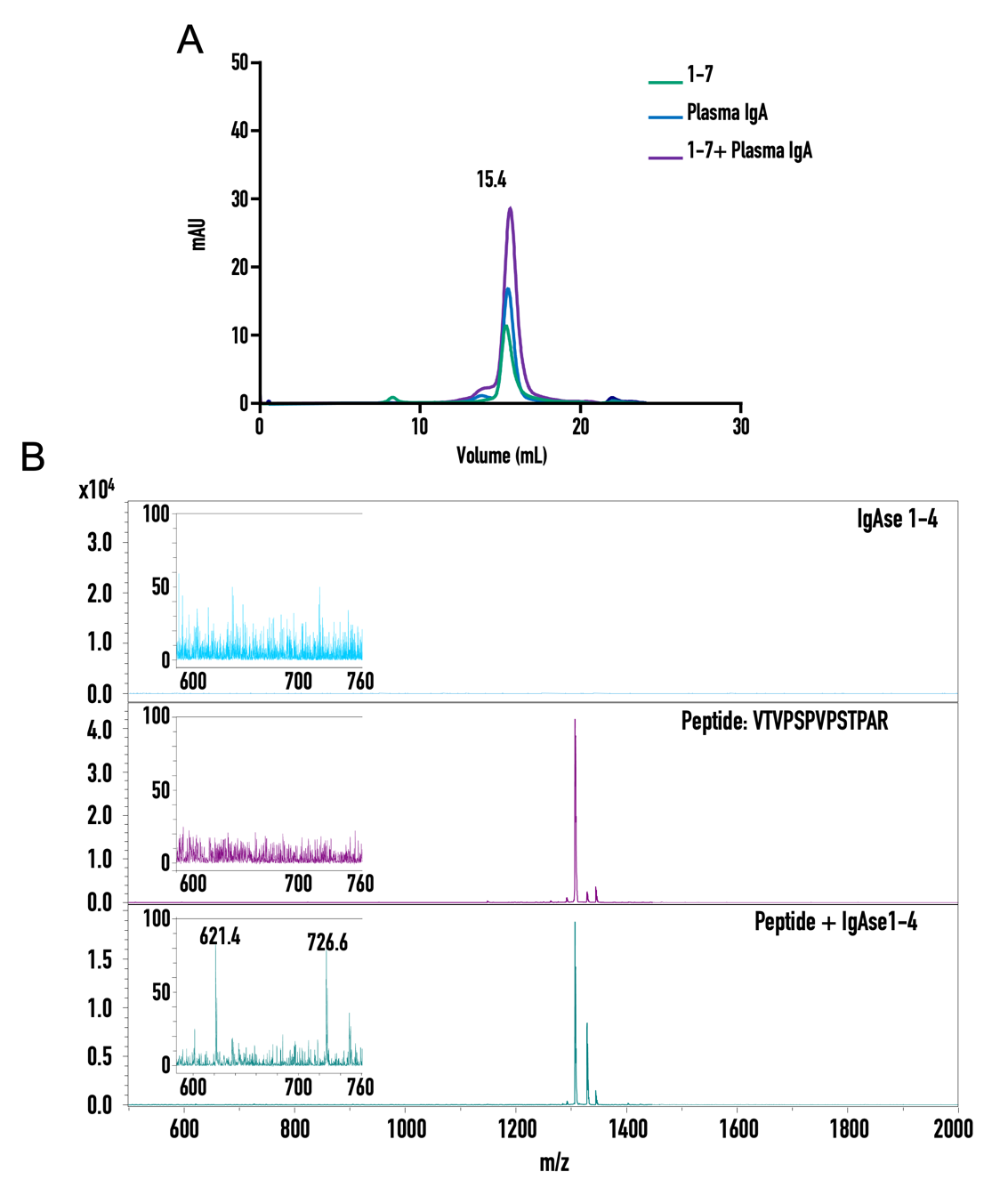


**S7 Fig — Transient enzyme–substrate complex and MALDI-TOF mass spectrometry**. **(A)** Independent SEC runs in a Superdex 200 10/300 GL column of plasma IgA (blue curve), inactive IgAse**1–7**+E^540^A (green curve), and a preincubated mixture of both (purple curve). No stable complex was observed, indicating that the enzyme–substrate interaction is transient. **(B)** MALDI-TOF mass-spectrometry analysis demonstrating that IgAse**1–4** (alone; top panel in blue) cleaves a synthetic peptide mimicking the IgA1 hinge region (see Section 3.4) (alone; middle panel in purple; peak at m/z 1306.7 [M+H]+) after overnight incubation, but only very poorly (bottom panel in green). The reaction, which results in less than 1% substrate turnover, gives rise to peaks at 621.4 and 726.6 m/z, as evident from the m/z 580–760 inset in green. These correspond to the VTVPSP ([M+H+Na]+) and VPSTPAR ([M+H]+) cleavage products. All three insets are scaled to the same y-axis values for comparison.
