# Supplementary material for: Biochemical and structural characterization of the human gut microbiome metallopeptidase IgAse provides insight into its unique specificity for the F ab ’ region of IgA1 and IgA2
Source: PLoS Pathog. 2025 Jul 8;21(7):e1013292. doi: 10.1371/journal.ppat.1013292 (PMC12237041; doi:10.1371/journal.ppat.1013292)
Supplement: S8 Fig — (A) Flowchart depicting cryo-electron microscopy single-particle analysis and image processing of the medium/low-resolution movies from a 200-kV Glacios microscope. (B) Gold-standard (0.143) Fourier Shell Correlation (GSFSC) curves. Model fitting was performed using our X-ray structure (CD + WD + Oβ) and the NTD plus connecting linker as modelled by AlphaFold3. (DOCX) [file ppat.1013292.s008.docx]

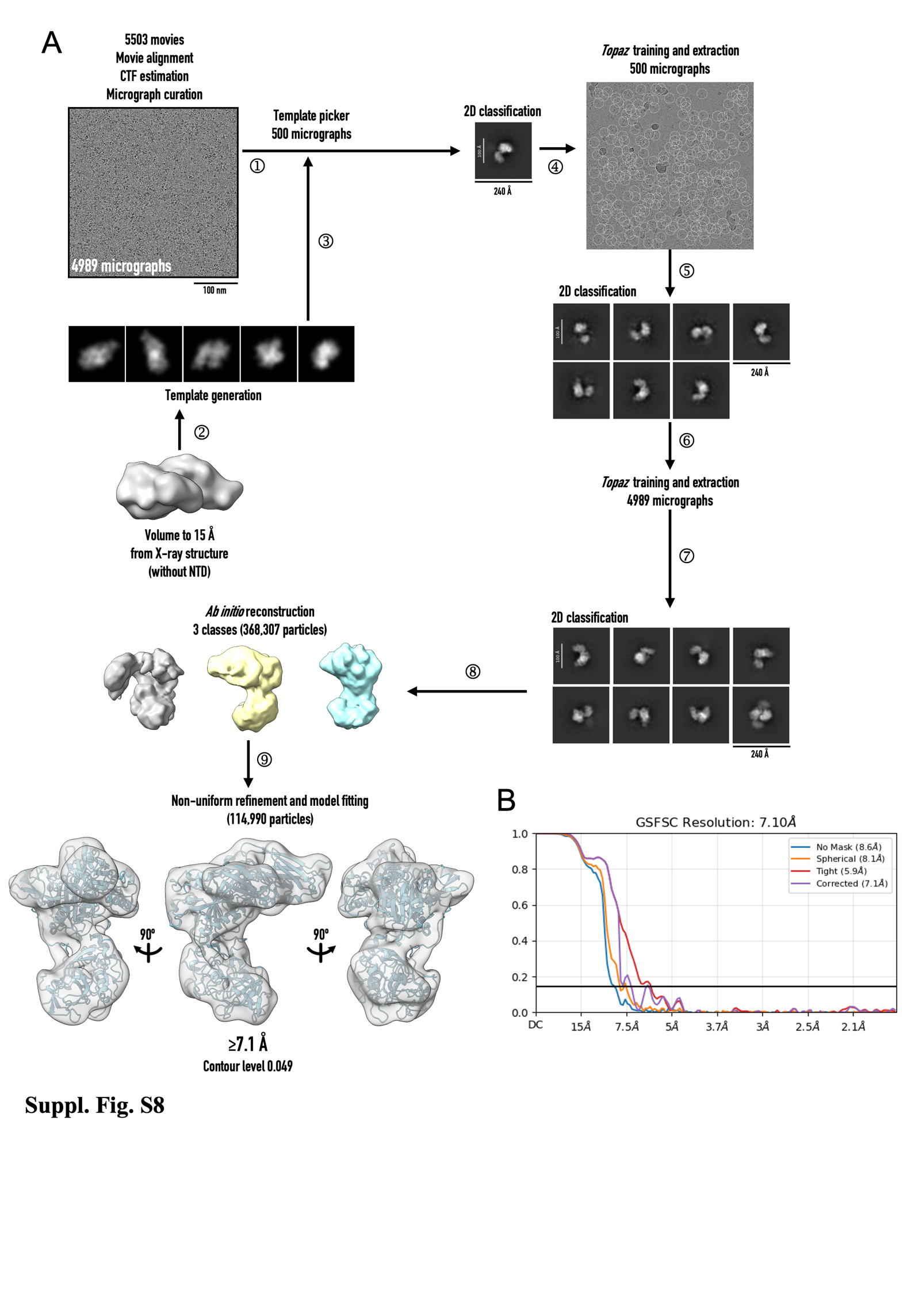


**S8 Fig — Cryo-EM data processing. (A)** Flowchart depicting cryo-electron microscopy single-particle analysis and image processing of the medium/low-resolution movies from a 200-kV Glacios microscope. **(B)** Gold-standard (0.143) Fourier Shell Correlation (GSFSC) curves. Model fitting was performed using our X-ray structure (CD+WD+Oβ) and the NTD plus connecting linker as modelled by *AlphaFold3*.
