# Supplementary material for: Biochemical and structural characterization of the human gut microbiome metallopeptidase IgAse provides insight into its unique specificity for the F ab ’ region of IgA1 and IgA2
Source: PLoS Pathog. 2025 Jul 8;21(7):e1013292. doi: 10.1371/journal.ppat.1013292 (PMC12237041; doi:10.1371/journal.ppat.1013292)
Supplement: S9 Fig — (A) Experimental SEC-SAXS profile of IgAse1–7 (blue). (B) Normalized Kratky plot derived from the SAXS profile shown in (A). (C) P(r) distribution fit (red curve) to the experimental SAXS data (blue curve) (χ2 = 1.05). (D) P(r) distribution showing a maximum linear dimension (dmax) of 278 Å. (DOCX) [file ppat.1013292.s009.docx]

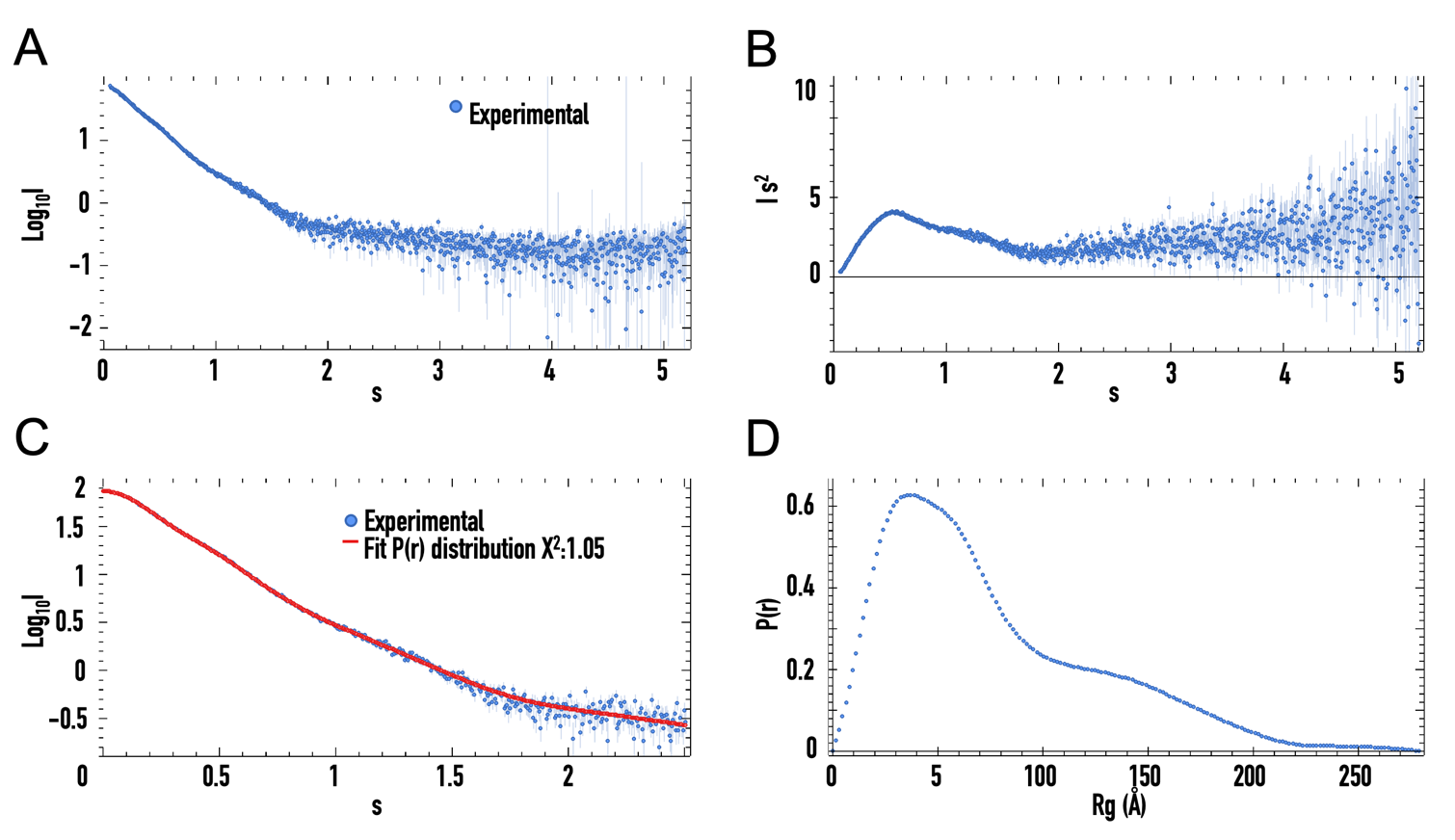


**S9 Fig — SEC-SAXS analysis of IgAse1–7 using the full data range. (A)** Experimental SEC-SAXS profile of IgAse**1–7** (blue). **(B)** Normalized Kratky plot derived from the SAXS profile shown in (A). **(C)** P(r) distribution fit (red curve) to the experimental SAXS data (blue curve) (χ^2^ =1.05). **(D)** P(r) distribution showing a maximum linear dimension (d_max_) of 278 Å.
