# Supplementary material for: Biochemical and structural characterization of the human gut microbiome metallopeptidase IgAse provides insight into its unique specificity for the F ab ’ region of IgA1 and IgA2
Source: PLoS Pathog. 2025 Jul 8;21(7):e1013292. doi: 10.1371/journal.ppat.1013292 (PMC12237041; doi:10.1371/journal.ppat.1013292)
Supplement: S10 Fig — Reducing SDS-PAGE analysis showing quantitative cleavage in the hinge region (lane 4, red asterisk) of C-terminally truncated wild-type (WT) IgA2Δ3 (lane 3) after overnight incubation with IgAse1–4 (lane 1). In contrast, mutants T84R/S94Y (lanes 5 and 6) and D96R/S94Y/T98W (lanes 7 and 8) are not cleaved. Lane 2 depicts the BlueStar Plus Molecular Weight ladder. (DOCX) [file ppat.1013292.s010.docx]

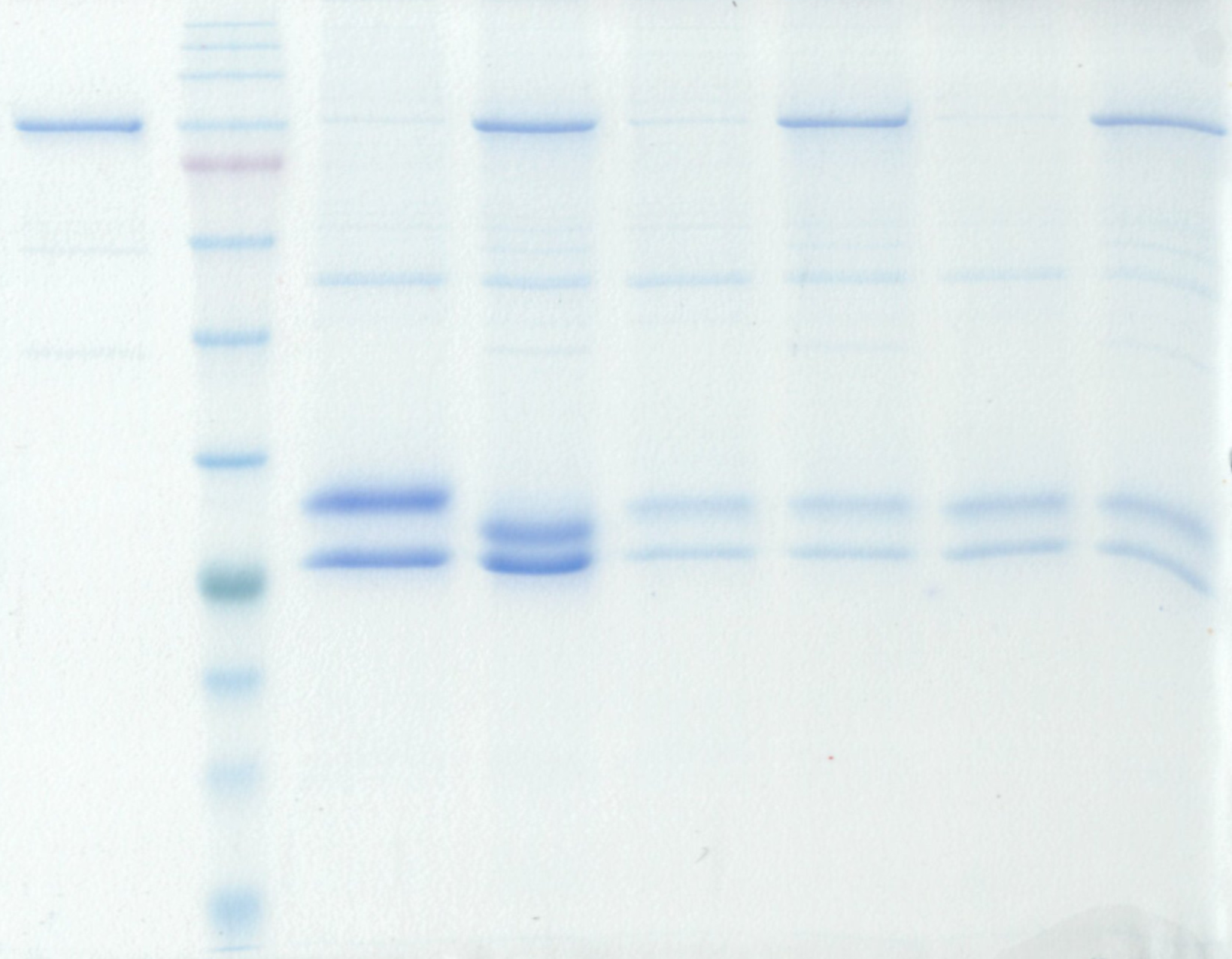


IgAse 1-4

M

WT

+

+

+

+

-

-

-

D^96^R/S^94^Y

T^84^R/S^94^Y/T^98^W

IgA2Δ3

**- HCΔ3**

**- LC**

*

100-

48-

25-

20-

180-

-IgAse

**S10 Fig — IgAse activity analysis against wild-type and mutant IgA2Δ3.** Reducing SDS-PAGE analysis showing quantitative cleavage in the hinge region (*lane* 4, red asterisk) of C-terminally truncated wild-type (WT) IgA2Δ3 (*lane* 3) after overnight incubation with IgAse**1–4** (*lane* 1). In contrast, mutants D^96^R/S^94^Y (*lanes* 5 and 6) and T^84^R/S^94^Y/T^98^W (*lanes* 7 and 8) are not cleaved. *Lane* 2 depicts the BlueStar Plus Molecular Weight ladder.
