# Supplementary material for: Biochemical and structural characterization of the human gut microbiome metallopeptidase IgAse provides insight into its unique specificity for the F ab ’ region of IgA1 and IgA2
Source: PLoS Pathog. 2025 Jul 8;21(7):e1013292. doi: 10.1371/journal.ppat.1013292 (PMC12237041; doi:10.1371/journal.ppat.1013292)
Supplement: S1 Table — (PPTX) [file ppat.1013292.s011.pptx]

## Slide 1
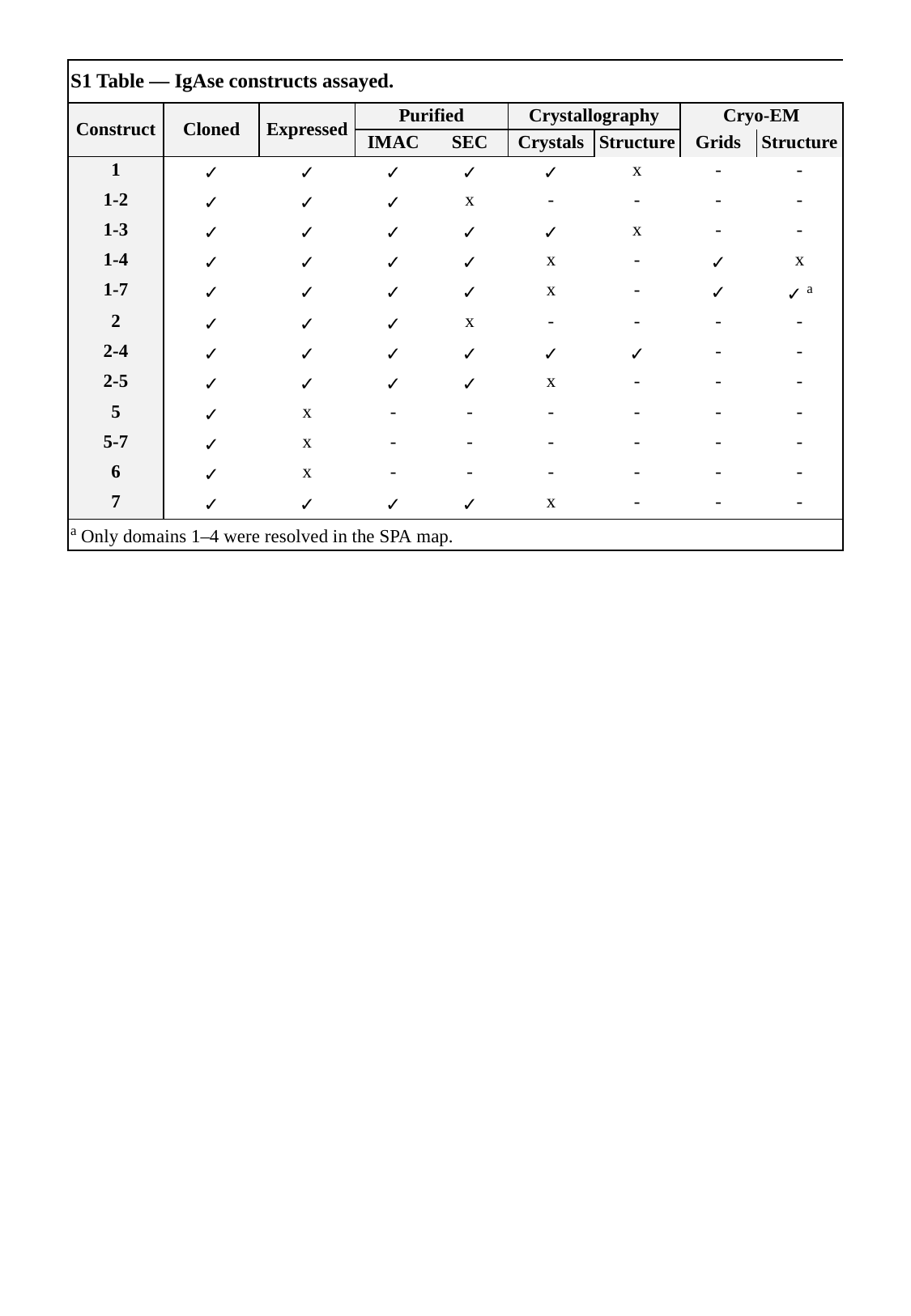

| S1 Table — IgAse constructs assayed. | | | | | | | | |
| --- | --- | --- | --- | --- | --- | --- | --- | --- |
| Construct | Cloned | Expressed | Purified | | Crystallography | | Cryo-EM | |
| | | | IMAC | SEC | Crystals | Structure | Grids | Structure |
| 1 | ✓ | ✓ | ✓ | ✓ | ✓ | x | - | - |
| 1-2 | ✓ | ✓ | ✓ | x | - | - | - | - |
| 1-3 | ✓ | ✓ | ✓ | ✓ | ✓ | x | - | - |
| 1-4 | ✓ | ✓ | ✓ | ✓ | x | - | ✓ | x |
| 1-7 | ✓ | ✓ | ✓ | ✓ | x | - | ✓ | ✓ a |
| 2 | ✓ | ✓ | ✓ | x | - | - | - | - |
| 2-4 | ✓ | ✓ | ✓ | ✓ | ✓ | ✓ | - | - |
| 2-5 | ✓ | ✓ | ✓ | ✓ | x | - | - | - |
| 5 | ✓ | x | - | - | - | - | - | - |
| 5-7 | ✓ | x | - | - | - | - | - | - |
| 6 | ✓ | x | - | - | - | - | - | - |
| 7 | ✓ | ✓ | ✓ | ✓ | x | - | - | - |
| a Only domains 1–4 were resolved in the SPA map. | | | | | | | | |
