# Supplementary material for: Biochemical and structural characterization of the human gut microbiome metallopeptidase IgAse provides insight into its unique specificity for the F ab ’ region of IgA1 and IgA2
Source: PLoS Pathog. 2025 Jul 8;21(7):e1013292. doi: 10.1371/journal.ppat.1013292 (PMC12237041; doi:10.1371/journal.ppat.1013292)
Supplement: S2 Table — (PPTX) [file ppat.1013292.s012.pptx]

## Slide 1
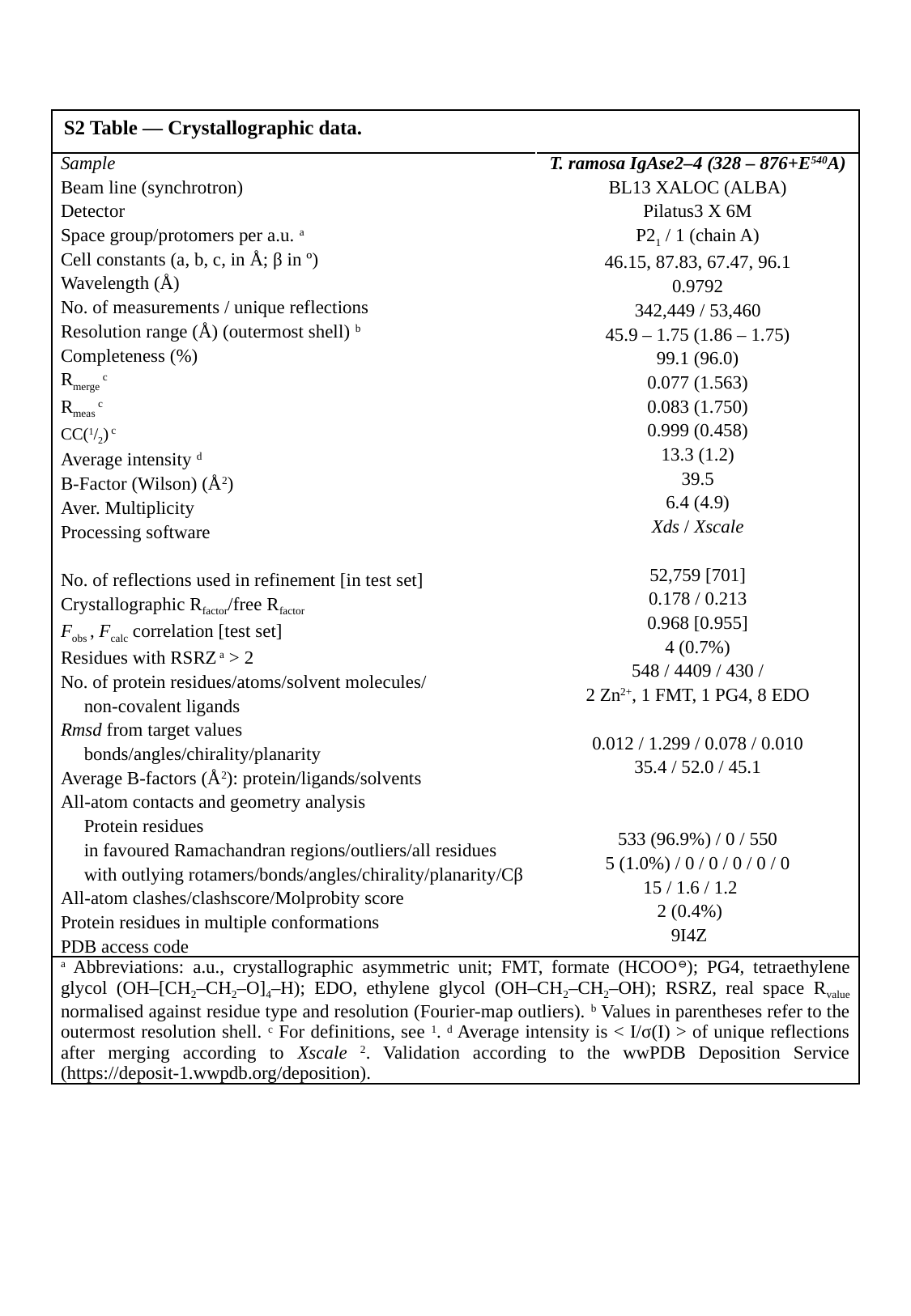

| S2 Table — Crystallographic data. | |
| --- | --- |
| Sample Beam line (synchrotron) Detector Space group/protomers per a.u. a Cell constants (a, b, c, in Å; β in º) Wavelength (Å) No. of measurements / unique reflections Resolution range (Å) (outermost shell) b Completeness (%) Rmerge c Rmeas c CC(1/2) c Average intensity d B-Factor (Wilson) (Å2) Aver. Multiplicity Processing software   No. of reflections used in refinement [in test set] Crystallographic Rfactor/free Rfactor Fobs , Fcalc correlation [test set] Residues with RSRZ a > 2 No. of protein residues/atoms/solvent molecules/ non-covalent ligands Rmsd from target values bonds/angles/chirality/planarity Average B-factors (Å2): protein/ligands/solvents All-atom contacts and geometry analysis Protein residues in favoured Ramachandran regions/outliers/all residues with outlying rotamers/bonds/angles/chirality/planarity/Cβ All-atom clashes/clashscore/Molprobity score Protein residues in multiple conformations PDB access code | T. ramosa IgAse2–4 (328 – 876+E540A) BL13 XALOC (ALBA) Pilatus3 X 6M P21 / 1 (chain A) 46.15, 87.83, 67.47, 96.1 0.9792 342,449 / 53,460 45.9 – 1.75 (1.86 – 1.75) 99.1 (96.0) 0.077 (1.563) 0.083 (1.750) 0.999 (0.458) 13.3 (1.2) 39.5 6.4 (4.9) Xds / Xscale   52,759 [701] 0.178 / 0.213 0.968 [0.955] 4 (0.7%) 548 / 4409 / 430 / 2 Zn2+, 1 FMT, 1 PG4, 8 EDO   0.012 / 1.299 / 0.078 / 0.010 35.4 / 52.0 / 45.1     533 (96.9%) / 0 / 550 5 (1.0%) / 0 / 0 / 0 / 0 / 0 15 / 1.6 / 1.2 2 (0.4%) 9I4Z |
| a Abbreviations: a.u., crystallographic asymmetric unit; FMT, formate (HCOO⊖); PG4, tetraethylene glycol (OH–[CH2–CH2–O]4–H); EDO, ethylene glycol (OH–CH2–CH2–OH); RSRZ, real space Rvalue normalised against residue type and resolution (Fourier-map outliers). b Values in parentheses refer to the outermost resolution shell. c For definitions, see 1. d Average intensity is < I/σ(I) > of unique reflections after merging according to Xscale 2. Validation according to the wwPDB Deposition Service (https://deposit-1.wwpdb.org/deposition). | |
