# Supplementary material for: Biochemical and structural characterization of the human gut microbiome metallopeptidase IgAse provides insight into its unique specificity for the F ab ’ region of IgA1 and IgA2
Source: PLoS Pathog. 2025 Jul 8;21(7):e1013292. doi: 10.1371/journal.ppat.1013292 (PMC12237041; doi:10.1371/journal.ppat.1013292)
Supplement: S3 Table — (PPTX) [file ppat.1013292.s013.pptx]

## Slide 1
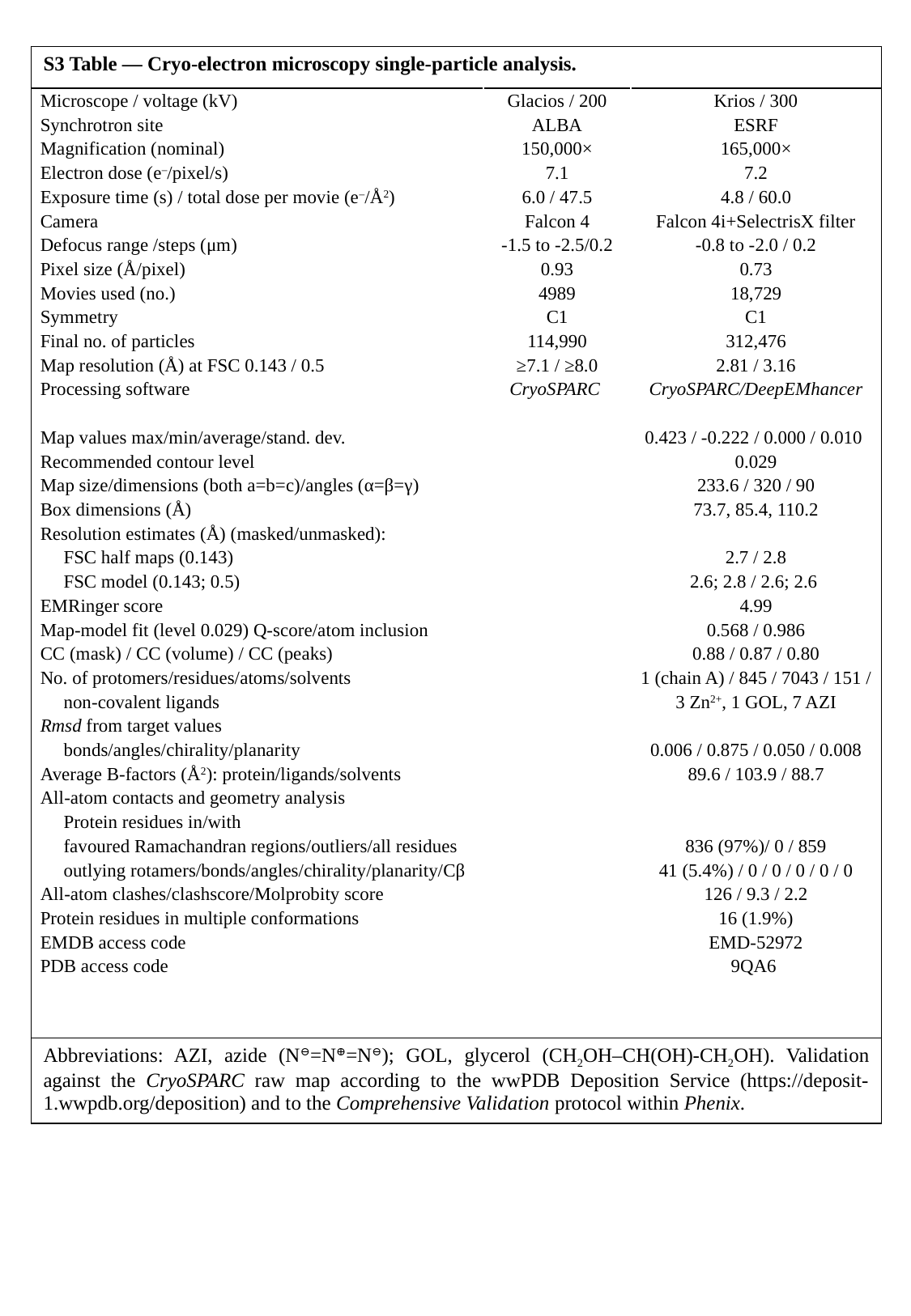

| S3 Table — Cryo-electron microscopy single-particle analysis. | | |
| --- | --- | --- |
| Microscope / voltage (kV) Synchrotron site Magnification (nominal) Electron dose (e–/pixel/s) Exposure time (s) / total dose per movie (e–/Å2) Camera Defocus range /steps (μm) Pixel size (Å/pixel) Movies used (no.) Symmetry Final no. of particles Map resolution (Å) at FSC 0.143 / 0.5 Processing software Map values max/min/average/stand. dev. Recommended contour level Map size/dimensions (both a=b=c)/angles (α=β=γ) Box dimensions (Å) Resolution estimates (Å) (masked/unmasked): FSC half maps (0.143) FSC model (0.143; 0.5) EMRinger score Map-model fit (level 0.029) Q-score/atom inclusion CC (mask) / CC (volume) / CC (peaks) No. of protomers/residues/atoms/solvents non-covalent ligands Rmsd from target values bonds/angles/chirality/planarity Average B-factors (Å2): protein/ligands/solvents All-atom contacts and geometry analysis Protein residues in/with favoured Ramachandran regions/outliers/all residues outlying rotamers/bonds/angles/chirality/planarity/Cβ All-atom clashes/clashscore/Molprobity score Protein residues in multiple conformations EMDB access code PDB access code | Glacios / 200 ALBA 150,000× 7.1 6.0 / 47.5 Falcon 4 -1.5 to -2.5/0.2 0.93 4989 C1 114,990 ≥7.1 / ≥8.0 CryoSPARC | Krios / 300 ESRF 165,000× 7.2 4.8 / 60.0 Falcon 4i+SelectrisX filter -0.8 to -2.0 / 0.2 0.73 18,729 C1 312,476 2.81 / 3.16 CryoSPARC/DeepEMhancer 0.423 / -0.222 / 0.000 / 0.010 0.029 233.6 / 320 / 90 73.7, 85.4, 110.2   2.7 / 2.8 2.6; 2.8 / 2.6; 2.6 4.99 0.568 / 0.986 0.88 / 0.87 / 0.80 1 (chain A) / 845 / 7043 / 151 / 3 Zn2+, 1 GOL, 7 AZI   0.006 / 0.875 / 0.050 / 0.008 89.6 / 103.9 / 88.7     836 (97%)/ 0 / 859 41 (5.4%) / 0 / 0 / 0 / 0 / 0 126 / 9.3 / 2.2 16 (1.9%) EMD-52972 9QA6 |
| Abbreviations: AZI, azide (N⊖=N⊕=N⊖); GOL, glycerol (CH2OH–CH(OH)-CH2OH). Validation against the CryoSPARC raw map according to the wwPDB Deposition Service (https://deposit-1.wwpdb.org/deposition) and to the Comprehensive Validation protocol within Phenix. | | |
