# Supplementary material for: Biochemical and structural characterization of the human gut microbiome metallopeptidase IgAse provides insight into its unique specificity for the F ab ’ region of IgA1 and IgA2
Source: PLoS Pathog. 2025 Jul 8;21(7):e1013292. doi: 10.1371/journal.ppat.1013292 (PMC12237041; doi:10.1371/journal.ppat.1013292)
Supplement: S4 Table — (PPTX) [file ppat.1013292.s014.pptx]

## Slide 1
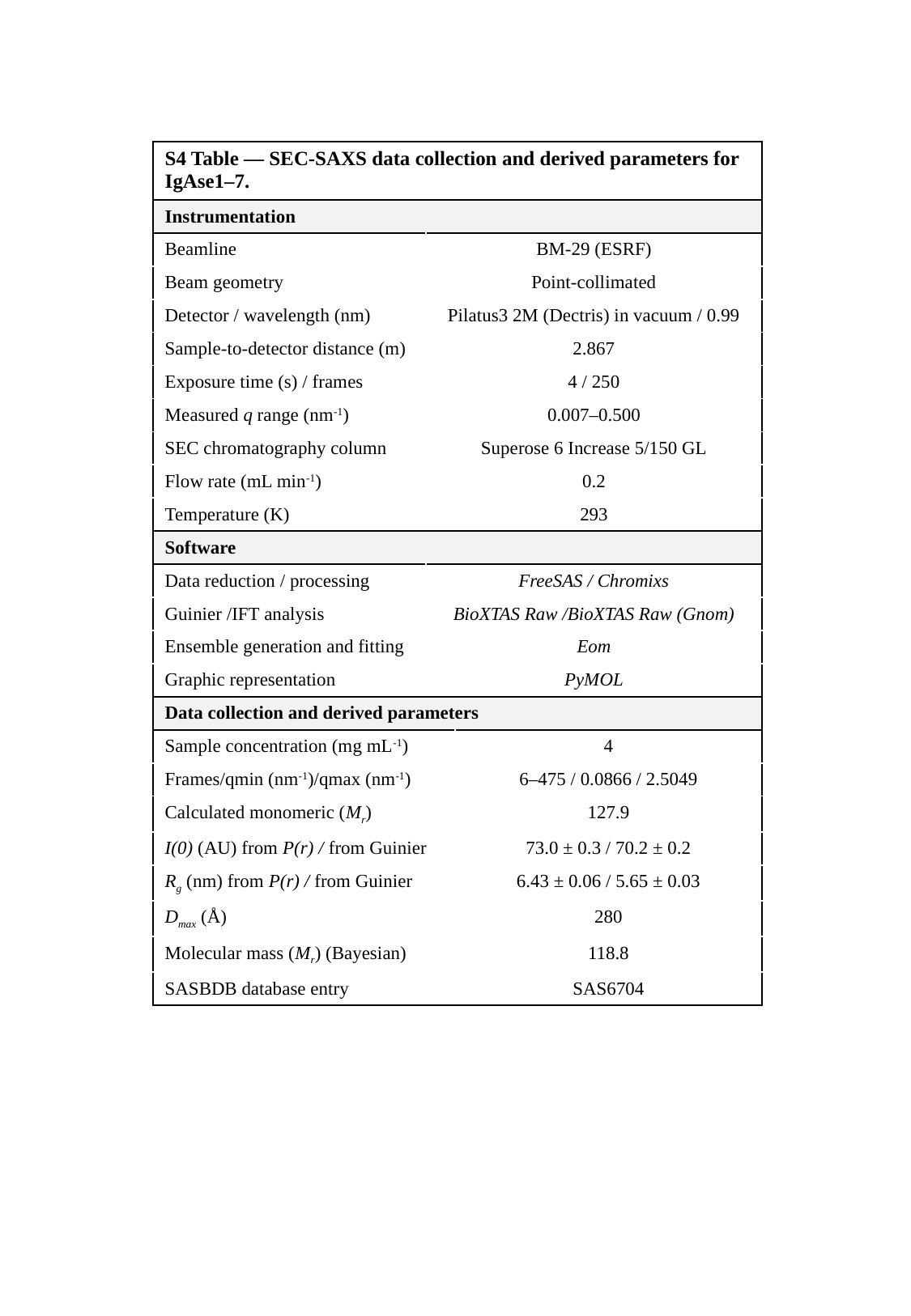

| S4 Table — SEC-SAXS data collection and derived parameters for IgAse1–7. | | |
| --- | --- | --- |
| Instrumentation | | |
| Beamline | BM-29 (ESRF) | |
| Beam geometry | Point-collimated | |
| Detector / wavelength (nm) | Pilatus3 2M (Dectris) in vacuum / 0.99 | |
| Sample-to-detector distance (m) | 2.867 | |
| Exposure time (s) / frames | 4 / 250 | |
| Measured q range (nm-1) | 0.007–0.500 | |
| SEC chromatography column | Superose 6 Increase 5/150 GL | |
| Flow rate (mL min-1) | 0.2 | |
| Temperature (K) | 293 | |
| Software | | |
| Data reduction / processing | FreeSAS / Chromixs | |
| Guinier /IFT analysis | BioXTAS Raw /BioXTAS Raw (Gnom) | |
| Ensemble generation and fitting | Eom | |
| Graphic representation | PyMOL | |
| Data collection and derived parameters | | |
| Sample concentration (mg mL-1) | 4 | 4 |
| Frames/qmin (nm-1)/qmax (nm-1) | 6–475 / 0.0866 / 2.5049 | 6–475 / 0.0866 / 2.5049 |
| Calculated monomeric (Mr) | 127.9 | 127.9 |
| I(0) (AU) from P(r) / from Guinier | | 73.0 ± 0.3 / 70.2 ± 0.2 |
| Rg (nm) from P(r) / from Guinier | | 6.43 ± 0.06 / 5.65 ± 0.03 |
| Dmax (Å) | | 280 |
| Molecular mass (Mr) (Bayesian) | | 118.8 |
| SASBDB database entry | | SAS6704 |
