# Supplementary material for: Biochemical and structural characterization of the human gut microbiome metallopeptidase IgAse provides insight into its unique specificity for the F ab ’ region of IgA1 and IgA2
Source: PLoS Pathog. 2025 Jul 8;21(7):e1013292. doi: 10.1371/journal.ppat.1013292 (PMC12237041; doi:10.1371/journal.ppat.1013292)
Supplement: S5 Table — (PPTX) [file ppat.1013292.s015.pptx]

## Slide 1
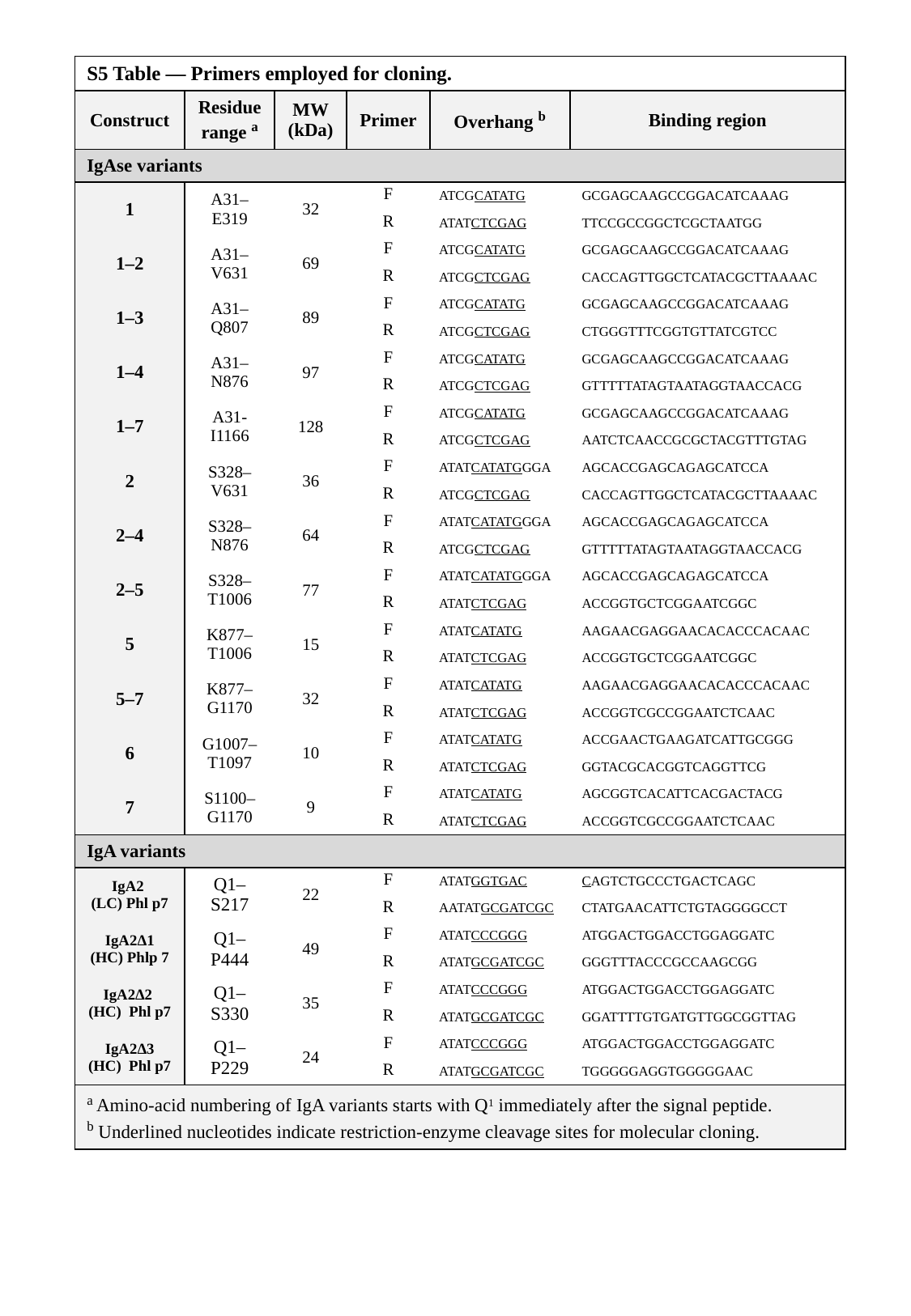

| S5 Table — Primers employed for cloning. | | | | | |
| --- | --- | --- | --- | --- | --- |
| Construct | Residue range a | MW (kDa) | Primer | Overhang b | Binding region |
| IgAse variants | | | | | |
| 1 | A31–E319 | 32 | F | ATCGCATATG | GCGAGCAAGCCGGACATCAAAG |
| | | | R | ATATCTCGAG | TTCCGCCGGCTCGCTAATGG |
| 1–2 | A31–V631 | 69 | F | ATCGCATATG | GCGAGCAAGCCGGACATCAAAG |
| | | | R | ATCGCTCGAG | CACCAGTTGGCTCATACGCTTAAAAC |
| 1–3 | A31–Q807 | 89 | F | ATCGCATATG | GCGAGCAAGCCGGACATCAAAG |
| | | | R | ATCGCTCGAG | CTGGGTTTCGGTGTTATCGTCC |
| 1–4 | A31–N876 | 97 | F | ATCGCATATG | GCGAGCAAGCCGGACATCAAAG |
| | | | R | ATCGCTCGAG | GTTTTTATAGTAATAGGTAACCACG |
| 1–7 | A31-I1166 | 128 | F | ATCGCATATG | GCGAGCAAGCCGGACATCAAAG |
| | | | R | ATCGCTCGAG | AATCTCAACCGCGCTACGTTTGTAG |
| 2 | S328–V631 | 36 | F | ATATCATATGGGA | AGCACCGAGCAGAGCATCCA |
| | | | R | ATCGCTCGAG | CACCAGTTGGCTCATACGCTTAAAAC |
| 2–4 | S328–N876 | 64 | F | ATATCATATGGGA | AGCACCGAGCAGAGCATCCA |
| | | | R | ATCGCTCGAG | GTTTTTATAGTAATAGGTAACCACG |
| 2–5 | S328–T1006 | 77 | F | ATATCATATGGGA | AGCACCGAGCAGAGCATCCA |
| | | | R | ATATCTCGAG | ACCGGTGCTCGGAATCGGC |
| 5 | K877–T1006 | 15 | F | ATATCATATG | AAGAACGAGGAACACACCCACAAC |
| | | | R | ATATCTCGAG | ACCGGTGCTCGGAATCGGC |
| 5–7 | K877–G1170 | 32 | F | ATATCATATG | AAGAACGAGGAACACACCCACAAC |
| | | | R | ATATCTCGAG | ACCGGTCGCCGGAATCTCAAC |
| 6 | G1007–T1097 | 10 | F | ATATCATATG | ACCGAACTGAAGATCATTGCGGG |
| | | | R | ATATCTCGAG | GGTACGCACGGTCAGGTTCG |
| 7 | S1100–G1170 | 9 | F | ATATCATATG | AGCGGTCACATTCACGACTACG |
| | | | R | ATATCTCGAG | ACCGGTCGCCGGAATCTCAAC |
| IgA variants | | | | | |
| IgA2 (LC) Phl p7 | Q1–S217 | 22 | F | ATATGGTGAC | CAGTCTGCCCTGACTCAGC |
| | | | R | AATATGCGATCGC | CTATGAACATTCTGTAGGGGCCT |
| IgA2Δ1 (HC) Phlp 7 | Q1–P444 | 49 | F | ATATCCCGGG | ATGGACTGGACCTGGAGGATC |
| | | | R | ATATGCGATCGC | GGGTTTACCCGCCAAGCGG |
| IgA2Δ2 (HC) Phl p7 | Q1–S330 | 35 | F | ATATCCCGGG | ATGGACTGGACCTGGAGGATC |
| | | | R | ATATGCGATCGC | GGATTTTGTGATGTTGGCGGTTAG |
| IgA2Δ3 (ΗC) Phl p7 | Q1–P229 | 24 | F | ATATCCCGGG | ATGGACTGGACCTGGAGGATC |
| | | | R | ATATGCGATCGC | TGGGGGAGGTGGGGGAAC |
| a Amino-acid numbering of IgA variants starts with Q1 immediately after the signal peptide. b Underlined nucleotides indicate restriction-enzyme cleavage sites for molecular cloning. | | | | | |
