# Supplementary material for: Correction: Biochemical and structural characterization of the human gut microbiome metallopeptidase IgAse provides insight into its unique specificity for the Fab’ region of IgA1 and IgA2
Source: PLoS Pathog. 2025 Dec 4;21(12):e1013742. doi: 10.1371/journal.ppat.1013742 (PMC12677558; doi:10.1371/journal.ppat.1013742)
Supplement: S2 File — (PDF) [file ppat.1013742.s002.pdf]

Full wwPDB X-ray Structure Validation Report (i)

Jun 26, 2025 – 04:47 pm BST

PDB ID	:	9I4Z / pdb_00009i4z
Title	:	Crystal structure of Thomasclavelia ramosa IgA peptidase (IgAse) active site mutant (E330-N876)
Authors	:	Ramirez-Larrota, J.S.; Eckhard, U.; Gomis-Ruth, F.X.
Deposited on	:	2025-01-27
Resolution	:	1.75 Å(reported)

This is a Full wwPDB X-ray Structure Validation Report for a publicly released PDB entry.

We welcome your comments at *validation@mail.wwpdb.org*
A user guide is available at
https://www.wwpdb.org/validation/2017/XrayValidationReportHelp
with specific help available everywhere you see the (i) symbol.

The types of validation reports are described at
http://www.wwpdb.org/validation/2017/FAQs#types.

The following versions of software and data (see references (i)) were used in the production of this report:

MolProbity	:	4-5-2 with Phenix2.0rc1
Mogul	:	1.8.4, CSD as541be (2020)
Xtriage (Phenix)	:	2.0rc1
EDS	:	3.0
Percentile statistics	:	20231227.v01 (using entries in the PDB archive December 27th 2023)
CCP4	:	9.0.003 (Gargrove)
Density-Fitness	:	1.0.11
Ideal geometry (proteins)	:	Engh & Huber (2001)
Ideal geometry (DNA, RNA)	:	Parkinson et al. (1996)
Validation Pipeline (wwPDB-VP)	:	2.44

1 Overall quality at a glance (i)

The following experimental techniques were used to determine the structure:
X-RAY DIFFRACTION

The reported resolution of this entry is 1.75 Å.

Percentile scores (ranging between 0-100) for global validation metrics of the entry are shown in the following graphic. The table shows the number of entries on which the scores are based.

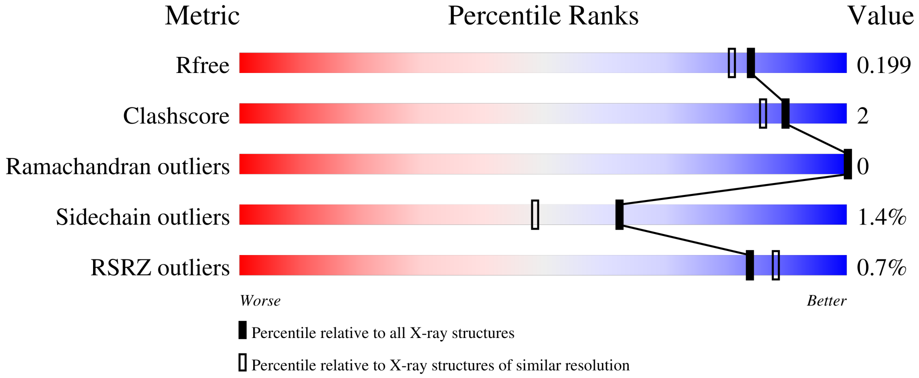

Metric	Whole archive (#Entries)	Similar resolution (#Entries, resolution range(Å))
R_{free}	164625	2888 (1.76-1.76)
Clashscore	180529	3097 (1.76-1.76)
Ramachandran outliers	177936	3072 (1.76-1.76)
Sidechain outliers	177891	3072 (1.76-1.76)
RSRZ outliers	164620	2887 (1.76-1.76)

The table below summarises the geometric issues observed across the polymeric chains and their fit to the electron density. The red, orange, yellow and green segments of the lower bar indicate the fraction of residues that contain outliers for >=3, 2, 1 and 0 types of geometric quality criteria respectively. A grey segment represents the fraction of residues that are not modelled. The numeric value for each fraction is indicated below the corresponding segment, with a dot representing fractions <=5% The upper red bar (where present) indicates the fraction of residues that have poor fit to the electron density. The numeric value is given above the bar.

Mol	Chain	Length	Quality of chain
1	A	559	% 92% 6% •

2 Entry composition (i)

There are 6 unique types of molecules in this entry. The entry contains 4924 atoms, of which 0 are hydrogens and 0 are deuteriums.

In the tables below, the ZeroOcc column contains the number of atoms modelled with zero occupancy, the AltConf column contains the number of residues with at least one atom in alternate conformation and the Trace column contains the number of residues modelled with at most 2 atoms.

- Molecule 1 is a protein called IgA protease.

Mol	Chain	Residues	Atoms					ZeroOcc	AltConf	Trace
1	A	548	Total 4430	C 2811	N 731	O 871	S 17	0	5	0

There are 11 discrepancies between the modelled and reference sequences:

Chain	Residue	Modelled	Actual	Comment	Reference
A	326	MET	-	initiating methionine	UNP Q9AES2
A	327	GLY	-	expression tag	UNP Q9AES2
A	540	ALA	GLU	engineered mutation	UNP Q9AES2
A	901	LEU	-	expression tag	UNP Q9AES2
A	902	GLU	-	expression tag	UNP Q9AES2
A	903	HIS	-	expression tag	UNP Q9AES2
A	904	HIS	-	expression tag	UNP Q9AES2
A	905	HIS	-	expression tag	UNP Q9AES2
A	906	HIS	-	expression tag	UNP Q9AES2
A	907	HIS	-	expression tag	UNP Q9AES2
A	908	HIS	-	expression tag	UNP Q9AES2

- Molecule 2 is ZINC ION (CCD ID: ZN) (formula: Zn) (labeled as "Ligand of Interest" by depositor).

Mol	Chain	Residues	Atoms		ZeroOcc	AltConf
2	A	2	Total 2	Zn 2	0	0

- Molecule 3 is TETRAETHYLENE GLYCOL (CCD ID: PG4) (formula: $C_8H_{18}O_5$).

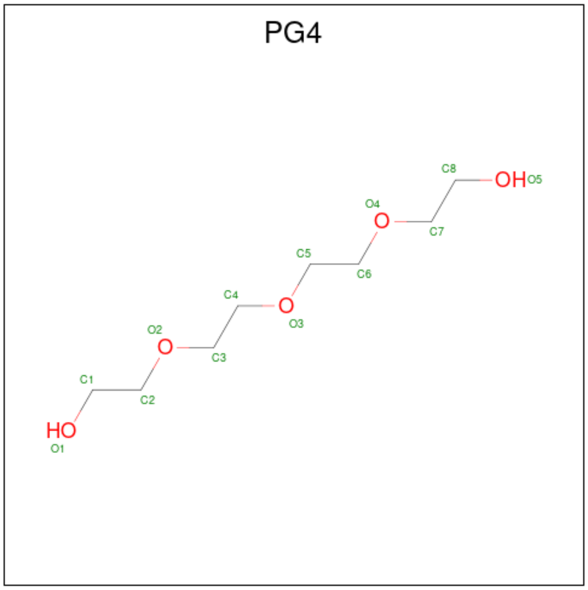

Mol	Chain	Residues	Atoms			ZeroOcc	AltConf
3	A	1	Total 13	C 8	O 5	0	0

- Molecule 4 is 1,2-ETHANEDIOL (CCD ID: EDO) (formula: $C_2H_6O_2$).

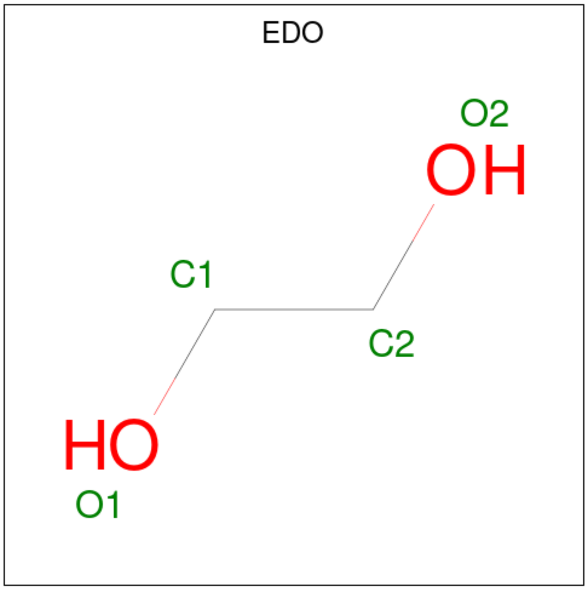

Mol	Chain	Residues	Atoms			ZeroOcc	AltConf
4	A	1	Total 4	C 2	O 2	0	0
4	A	1	Total 4	C 2	O 2	0	0
4	A	1	Total 4	C 2	O 2	0	0

Continued on next page...

Continued from previous page...

Mol	Chain	Residues	Atoms	ZeroOcc	AltConf
4	A	1	Total C O<br>4 2 2	0	0
4	A	1	Total C O<br>4 2 2	0	0
4	A	1	Total C O<br>4 2 2	0	0
4	A	1	Total C O<br>4 2 2	0	0
4	A	1	Total C O<br>4 2 2	0	0

- Molecule 5 is FORMIC ACID (CCD ID: FMT) (formula: CH_2O_2).

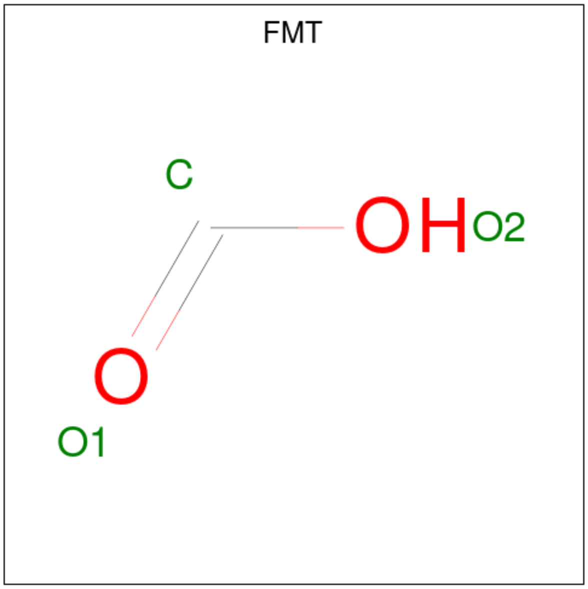

Mol	Chain	Residues	Atoms	ZeroOcc	AltConf
5	A	1	Total C O<br>3 1 2	0	0

- Molecule 6 is water.

Mol	Chain	Residues	Atoms	ZeroOcc	AltConf
6	A	430	Total O<br>444 444	0	14

3 Residue-property plots (i)

These plots are drawn for all protein, RNA, DNA and oligosaccharide chains in the entry. The first graphic for a chain summarises the proportions of the various outlier classes displayed in the second graphic. The second graphic shows the sequence view annotated by issues in geometry and electron density. Residues are color-coded according to the number of geometric quality criteria for which they contain at least one outlier: green = 0, yellow = 1, orange = 2 and red = 3 or more. A red dot above a residue indicates a poor fit to the electron density (RSRZ > 2). Stretches of 2 or more consecutive residues without any outlier are shown as a green connector. Residues present in the sample, but not in the model, are shown in grey.

- Molecule 1: IgA protease

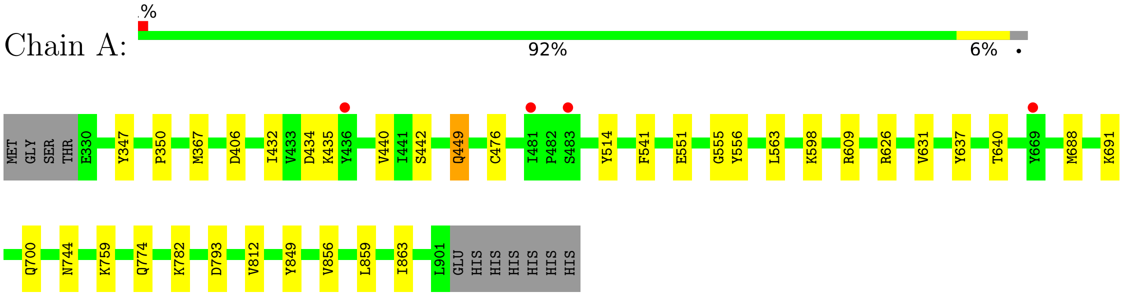

4 Data and refinement statistics

Property	Value	Source
Space group	P 1 21 1	Depositor
Cell constants<br>a, b, c, α, β, γ	46.15Å 87.83Å 67.47Å<br>90.00° 96.11° 90.00°	Depositor
Resolution (Å)	45.89 – 1.75<br>45.89 – 1.75	Depositor<br>EDS
% Data completeness<br>(in resolution range)	99.2 (45.89-1.75)<br>99.1 (45.89-1.75)	Depositor<br>EDS
R_{merge}	0.08	Depositor
R_{sym}	(Not available)	Depositor
$< I/\sigma(I) >$ [1]	1.09 (at 1.75Å)	Xtriage
Refinement program	BUSTER 2.10.4	Depositor
R, R_{free}	0.178 , 0.213<br>0.171 , 0.199	Depositor<br>DCC
R_{free} test set	1161 reflections (1.31%)	wwPDB-VP
Wilson B-factor (Å^2)	30.9	Xtriage
Anisotropy	0.144	Xtriage
Bulk solvent k_{sol}(e/Å^3), B_{sol}(Å^2)	0.33 , 37.6	EDS
L-test for twinning[2]	$< \|L\| >$ = 0.51, $< L^2 >$ = 0.34	Xtriage
Estimated twinning fraction	No twinning to report.	Xtriage
F_o,F_c correlation	0.97	EDS
Total number of atoms	4924	wwPDB-VP
Average B, all atoms (Å^2)	36.0	wwPDB-VP

Xtriage's analysis on translational NCS is as follows: *The largest off-origin peak in the Patterson function is 5.66% of the height of the origin peak. No significant pseudotranslation is detected.*

[1]Intensities estimated from amplitudes.

[2]Theoretical values of $< |L| >$, $< L^2 >$ for acentric reflections are 0.5, 0.333 respectively for untwinned datasets, and 0.375, 0.2 for perfectly twinned datasets.

5 Model quality (i)

5.1 Standard geometry (i)

Bond lengths and bond angles in the following residue types are not validated in this section: FMT, EDO, ZN, PG4

The Z score for a bond length (or angle) is the number of standard deviations the observed value is removed from the expected value. A bond length (or angle) with $|Z| > 5$ is considered an outlier worth inspection. RMSZ is the root-mean-square of all Z scores of the bond lengths (or angles).

Mol	Chain	Bond lengths		Bond angles	
		RMSZ	$\#\|Z\| >5$	RMSZ	$\#\|Z\| >5$
1	A	0.75	1/4555 (0.0%)	0.90	5/6177 (0.1%)

All (1) bond length outliers are listed below:

Mol	Chain	Res	Type	Atoms	Z	Observed(Å)	Ideal(Å)
1	A	849	TYR	CA-C	5.93	1.58	1.52

All (5) bond angle outliers are listed below:

Mol	Chain	Res	Type	Atoms	Z	Observed(^{o})	Ideal(^{o})
1	A	555	GLY	N-CA-C	7.15	120.98	112.33
1	A	434	ASP	CA-CB-CG	5.66	118.26	112.60
1	A	793	ASP	CA-CB-CG	5.58	118.18	112.60
1	A	406	ASP	CA-CB-CG	5.30	117.91	112.60
1	A	541	PHE	CA-CB-CG	5.19	118.99	113.80

There are no chirality outliers.

There are no planarity outliers.

5.2 Too-close contacts (i)

In the following table, the Non-H and H(model) columns list the number of non-hydrogen atoms and hydrogen atoms in the chain respectively. The H(added) column lists the number of hydrogen atoms added and optimized by MolProbity. The Clashes column lists the number of clashes within the asymmetric unit, whereas Symm-Clashes lists symmetry-related clashes.

Mol	Chain	Non-H	H(model)	H(added)	Clashes	Symm-Clashes
1	A	4430	0	4245	15	0
2	A	2	0	0	0	0

Continued on next page...

Continued from previous page...

Mol	Chain	Non-H	H(model)	H(added)	Clashes	Symm-Clashes
3	A	13	0	18	1	0
4	A	32	0	48	0	0
5	A	3	0	1	0	0
6	A	444	0	0	1	0
All	All	4924	0	4312	15	0

The all-atom clashscore is defined as the number of clashes found per 1000 atoms (including hydrogen atoms). The all-atom clashscore for this structure is 2.

All (15) close contacts within the same asymmetric unit are listed below, sorted by their clash magnitude.

Atom-1	Atom-2	Interatomic distance (Å)	Clash overlap (Å)
1:A:449:GLN:H	1:A:449:GLN:HE21	1.24	0.85
1:A:514:TYR:HA	3:A:1003:PG4:H32	1.66	0.76
1:A:432:ILE:HG12	1:A:442:SER:HB2	1.78	0.65
1:A:744:ASN:HB2	6:A:1161:HOH:O	2.03	0.58
1:A:688:MET:HA	1:A:691:LYS:HD2	1.87	0.57
1:A:563:LEU:HD13	1:A:759:LYS:HE2	1.90	0.54
1:A:556:TYR:HB2	1:A:598:LYS:HD3	1.93	0.51
1:A:812:VAL:HG22	1:A:863:ILE:HG21	1.93	0.51
1:A:626:ARG:HD3	1:A:626:ARG:C	2.39	0.47
1:A:637:TYR:HB3	1:A:700:GLN:HB3	2.00	0.43
1:A:367:MET:HG3	1:A:631:VAL:HG23	2.00	0.43
1:A:347:TYR:O	1:A:350:PRO:HD3	2.19	0.43
1:A:551:GLU:HB2	1:A:609:ARG:HB2	2.03	0.41
1:A:856:VAL:HG11	1:A:859:LEU:HD12	2.02	0.41
1:A:812:VAL:CG2	1:A:863:ILE:HG21	2.51	0.41

There are no symmetry-related clashes.

5.3 Torsion angles (i)

5.3.1 Protein backbone (i)

In the following table, the Percentiles column shows the percent Ramachandran outliers of the chain as a percentile score with respect to all X-ray entries followed by that with respect to entries of similar resolution.

The Analysed column shows the number of residues for which the backbone conformation was analysed, and the total number of residues.

Mol	Chain	Analysed	Favoured	Allowed	Outliers	Percentiles	
1	A	550/559 (98%)	534 (97%)	16 (3%)	0	100	100

There are no Ramachandran outliers to report.

5.3.2 Protein sidechains (i)

In the following table, the Percentiles column shows the percent sidechain outliers of the chain as a percentile score with respect to all X-ray entries followed by that with respect to entries of similar resolution.

The Analysed column shows the number of residues for which the sidechain conformation was analysed, and the total number of residues.

Mol	Chain	Analysed	Rotameric	Outliers	Percentiles	
1	A	494/499 (99%)	487 (99%)	7 (1%)	62	49

All (7) residues with a non-rotameric sidechain are listed below:

Mol	Chain	Res	Type
1	A	435	LYS
1	A	440	VAL
1	A	449	GLN
1	A	476	CYS
1	A	640	THR
1	A	774	GLN
1	A	782	LYS

Sometimes sidechains can be flipped to improve hydrogen bonding and reduce clashes. All (3) such sidechains are listed below:

Mol	Chain	Res	Type
1	A	437	ASN
1	A	449	GLN
1	A	780	GLN

5.3.3 RNA (i)

There are no RNA molecules in this entry.

5.4 Non-standard residues in protein, DNA, RNA chains (i)

There are no non-standard protein/DNA/RNA residues in this entry.

5.5 Carbohydrates (i)

There are no oligosaccharides in this entry.

5.6 Ligand geometry (i)

Of 12 ligands modelled in this entry, 2 are monoatomic - leaving 10 for Mogul analysis.

In the following table, the Counts columns list the number of bonds (or angles) for which Mogul statistics could be retrieved, the number of bonds (or angles) that are observed in the model and the number of bonds (or angles) that are defined in the Chemical Component Dictionary. The Link column lists molecule types, if any, to which the group is linked. The Z score for a bond length (or angle) is the number of standard deviations the observed value is removed from the expected value. A bond length (or angle) with $|Z| > 2$ is considered an outlier worth inspection. RMSZ is the root-mean-square of all Z scores of the bond lengths (or angles).

Mol	Type	Chain	Res	Link	Bond lengths			Bond angles		
					Counts	RMSZ	$\#\|Z\| > 2$	Counts	RMSZ	$\#\|Z\| > 2$
4	EDO	A	1008	-	3,3,3	0.34	0	2,2,2	0.28	0
4	EDO	A	1005	-	3,3,3	0.33	0	2,2,2	0.25	0
5	FMT	A	1012	2	2,2,2	1.59	1 (50%)	1,1,1	0.13	0
4	EDO	A	1009	-	3,3,3	0.26	0	2,2,2	0.28	0
4	EDO	A	1011	-	3,3,3	0.43	0	2,2,2	0.14	0
4	EDO	A	1010	-	3,3,3	0.30	0	2,2,2	0.19	0
3	PG4	A	1003	-	12,12,12	0.19	0	11,11,11	0.09	0
4	EDO	A	1006	-	3,3,3	0.20	0	2,2,2	0.41	0
4	EDO	A	1004	-	3,3,3	0.12	0	2,2,2	0.30	0
4	EDO	A	1007	-	3,3,3	0.27	0	2,2,2	0.28	0

In the following table, the Chirals column lists the number of chiral outliers, the number of chiral centers analysed, the number of these observed in the model and the number defined in the Chemical Component Dictionary. Similar counts are reported in the Torsion and Rings columns. '-' means no outliers of that kind were identified.

Mol	Type	Chain	Res	Link	Chirals	Torsions	Rings
4	EDO	A	1008	-	-	0/1/1/1	-
4	EDO	A	1005	-	-	0/1/1/1	-
4	EDO	A	1009	-	-	0/1/1/1	-
4	EDO	A	1011	-	-	0/1/1/1	-
4	EDO	A	1010	-	-	1/1/1/1	-

Continued on next page...

Continued from previous page...

Mol	Type	Chain	Res	Link	Chirals	Torsions	Rings
3	PG4	A	1003	-	-	5/10/10/10	-
4	EDO	A	1006	-	-	0/1/1/1	-
4	EDO	A	1004	-	-	0/1/1/1	-
4	EDO	A	1007	-	-	0/1/1/1	-

All (1) bond length outliers are listed below:

Mol	Chain	Res	Type	Atoms	Z	Observed(Å)	Ideal(Å)
5	A	1012	FMT	O2-C	2.16	1.39	1.28

There are no bond angle outliers.

There are no chirality outliers.

All (6) torsion outliers are listed below:

Mol	Chain	Res	Type	Atoms
3	A	1003	PG4	O3-C5-C6-O4
3	A	1003	PG4	O2-C3-C4-O3
4	A	1010	EDO	O1-C1-C2-O2
3	A	1003	PG4	C4-C3-O2-C2
3	A	1003	PG4	C6-C5-O3-C4
3	A	1003	PG4	C5-C6-O4-C7

There are no ring outliers.

1 monomer is involved in 1 short contact:

Mol	Chain	Res	Type	Clashes	Symm-Clashes
3	A	1003	PG4	1	0

5.7 Other polymers (i)

There are no such residues in this entry.

5.8 Polymer linkage issues (i)

There are no chain breaks in this entry.

6 Fit of model and data (i)

6.1 Protein, DNA and RNA chains (i)

In the following table, the column labelled '#RSRZ> 2' contains the number (and percentage) of RSRZ outliers, followed by percent RSRZ outliers for the chain as percentile scores relative to all X-ray entries and entries of similar resolution. The OWAB column contains the minimum, median, 95th percentile and maximum values of the occupancy-weighted average B-factor per residue. The column labelled 'Q< 0.9' lists the number of (and percentage) of residues with an average occupancy less than 0.9.

Mol	Chain	Analysed	<RSRZ>	#RSRZ>2			OWAB(Å^2)	Q<0.9
1	A	548/559 (98%)	-0.04	4 (0%)	84	88	18, 33, 57, 74	5 (0%)

All (4) RSRZ outliers are listed below:

Mol	Chain	Res	Type	RSRZ
1	A	483	SER	3.6
1	A	669	TYR	2.2
1	A	436	TYR	2.2
1	A	481	ILE	2.1

6.2 Non-standard residues in protein, DNA, RNA chains (i)

There are no non-standard protein/DNA/RNA residues in this entry.

6.3 Carbohydrates (i)

There are no oligosaccharides in this entry.

6.4 Ligands (i)

In the following table, the Atoms column lists the number of modelled atoms in the group and the number defined in the chemical component dictionary. The B-factors column lists the minimum, median, 95th percentile and maximum values of B factors of atoms in the group. The column labelled 'Q< 0.9' lists the number of atoms with occupancy less than 0.9.

Mol	Type	Chain	Res	Atoms	RSCC	RSR	B-factors(Å^2)	Q<0.9
4	EDO	A	1007	4/4	0.72	0.17	59,59,59,59	0
3	PG4	A	1003	13/13	0.74	0.17	57,60,63,63	0
4	EDO	A	1006	4/4	0.83	0.15	49,49,49,50	0

Continued on next page...

Continued from previous page...

Mol	Type	Chain	Res	Atoms	RSCC	RSR	B-factors($Å^2$)	Q<0.9
5	FMT	A	1012	3/3	0.86	0.14	48,48,49,49	0
4	EDO	A	1008	4/4	0.87	0.12	51,51,51,52	0
4	EDO	A	1010	4/4	0.89	0.11	61,61,61,61	0
4	EDO	A	1009	4/4	0.89	0.11	44,44,45,45	0
4	EDO	A	1004	4/4	0.92	0.13	60,60,60,60	0
4	EDO	A	1005	4/4	0.93	0.10	40,41,42,43	0
4	EDO	A	1011	4/4	0.97	0.06	29,30,32,32	0
2	ZN	A	1001	1/1	1.00	0.02	28,28,28,28	0
2	ZN	A	1002	1/1	1.00	0.02	32,32,32,32	0

The following is a graphical depiction of the model fit to experimental electron density of all instances of the Ligand of Interest. In addition, ligands with molecular weight > 250 and outliers as shown on the geometry validation Tables will also be included. Each fit is shown from different orientation to approximate a three-dimensional view.

Electron density around ZN A 1001:

$2mF_o$-DF_c (at 0.7 rmsd) in gray
mF_o-DF_c (at 3 rmsd) in purple (negative)
and green (positive)

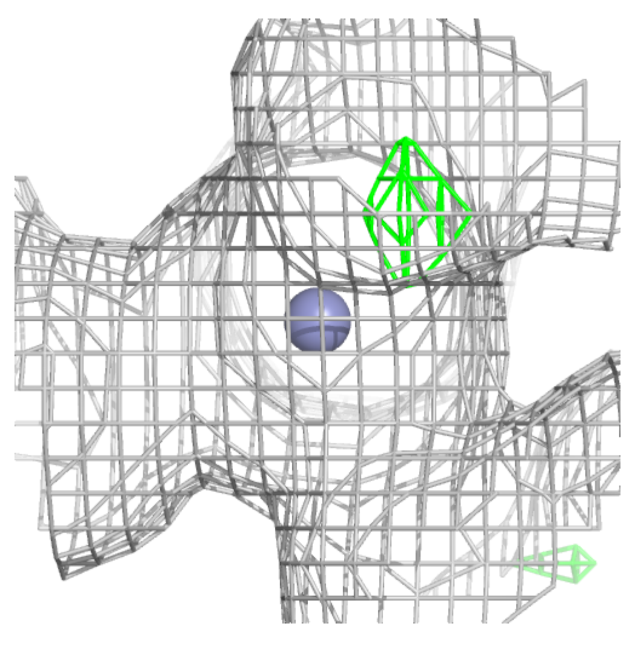

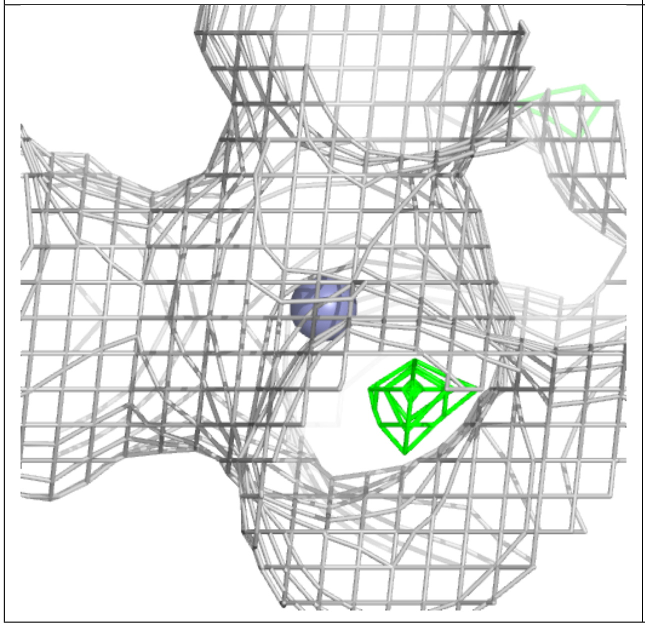

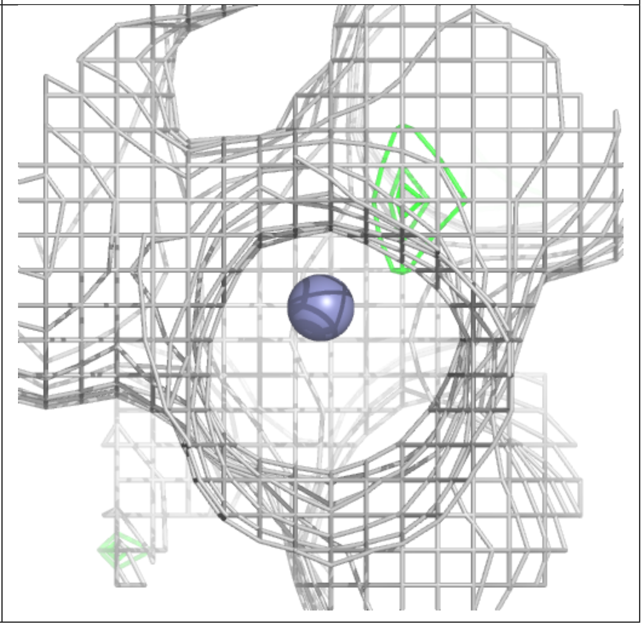

Electron density around ZN A 1002:

2mF$_o$-DF$_c$ (at 0.7 rmsd) in gray
mF$_o$-DF$_c$ (at 3 rmsd) in purple (negative)
and green (positive)

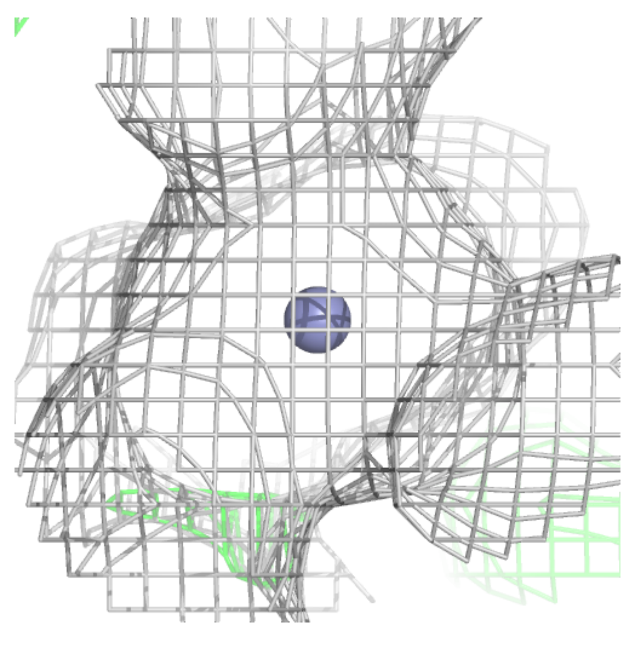

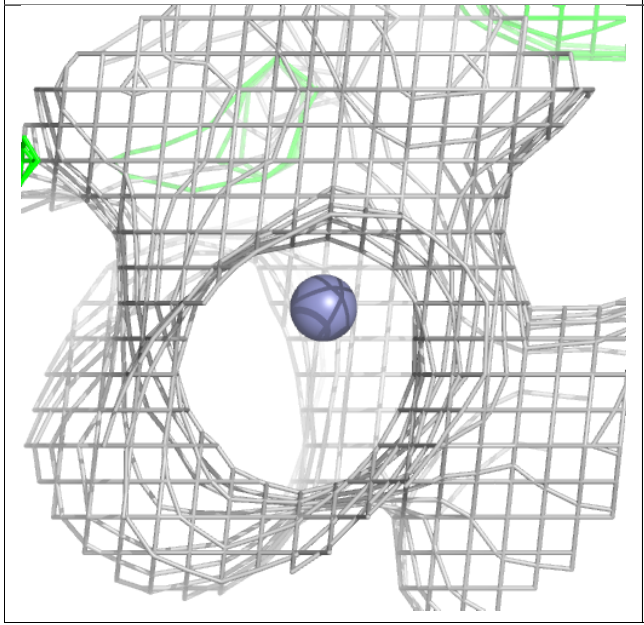

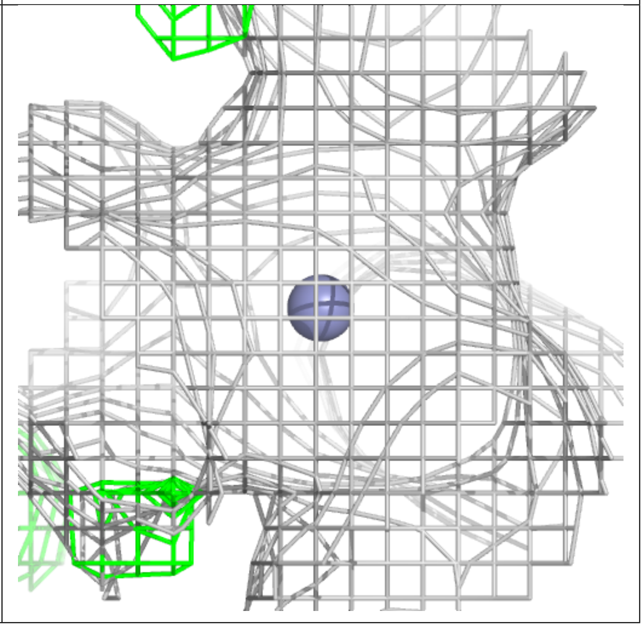

6.5 Other polymers (i)

There are no such residues in this entry.
