# Supplementary material for: Correction: Biochemical and structural characterization of the human gut microbiome metallopeptidase IgAse provides insight into its unique specificity for the Fab’ region of IgA1 and IgA2
Source: PLoS Pathog. 2025 Dec 4;21(12):e1013742. doi: 10.1371/journal.ppat.1013742 (PMC12677558; doi:10.1371/journal.ppat.1013742)
Supplement: S1 Fig — (PDF) [file ppat.1013742.s003.pdf]

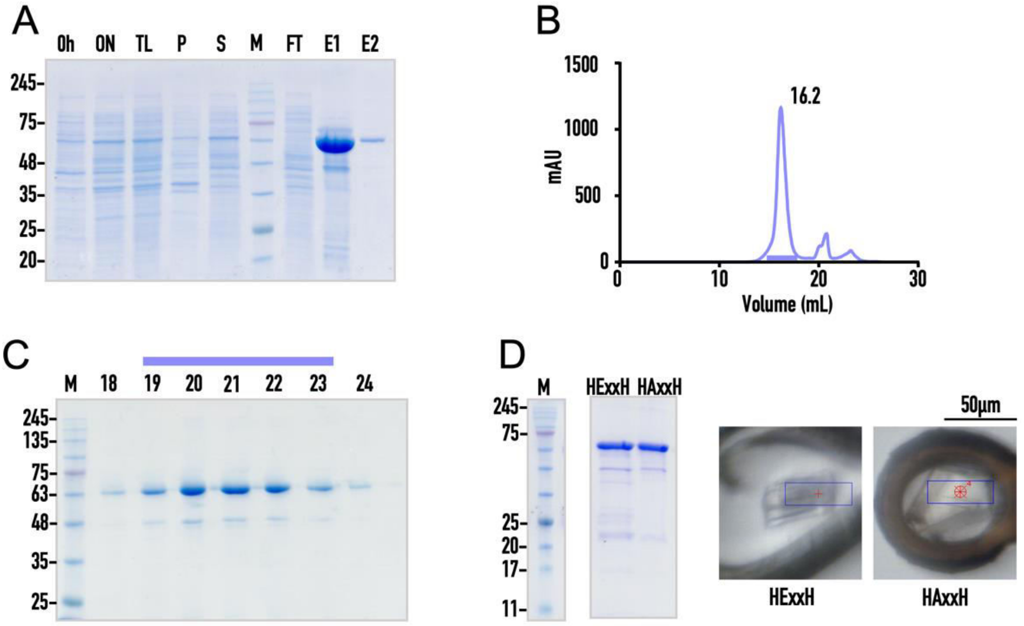

S1 Fig — Recombinant protein expression, purification, and crystallization of IgAse2–4. (A) Representative SDS-PAGE analysis illustrating protein expression, cell lysis, and IMAC purification. *Lanes*: 0h, pre-induction; ON, post-induction after overnight incubation at 20 °C; TL, total lysate; P, pellet; SN, soluble supernatant fraction; M, molecular mass marker; FT, flow-through; E1, elution 1 (250 mM imidazole); E2, elution 2 (250 mM imidazole). Of note, IgAse**2-4** exhibits an apparent molecular mass of ~63 kDa. **(B)** SEC profile of purified IgAse**2–4**+E^{540}A analysed using a Superdex 200 10/300 GL column, showing a monodisperse peak at a retention volume of ~16.2 mL, which corresponds to a monomer. Fractions selected for crystallization are indicated by a slate blue bar at the peak base. **(C)** Reducing SDS-PAGE analysis of the SEC run from (B), with fractions selected for crystallization indicated by a slate blue bar. **(D)** Reducing SDS-PAGE analysis of both active IgAse**2–4** (HExxH) and inactive IgAse**2–4**+E^{540}A (HAxxH) used for crystallization after protein concentration. On the *right*, representative images of diffracted crystals in their cryo-loops.
