# Supplementary material for: Correction: Biochemical and structural characterization of the human gut microbiome metallopeptidase IgAse provides insight into its unique specificity for the Fab’ region of IgA1 and IgA2
Source: PLoS Pathog. 2025 Dec 4;21(12):e1013742. doi: 10.1371/journal.ppat.1013742 (PMC12677558; doi:10.1371/journal.ppat.1013742)
Supplement: S3 Fig — (PDF) [file ppat.1013742.s005.pdf]

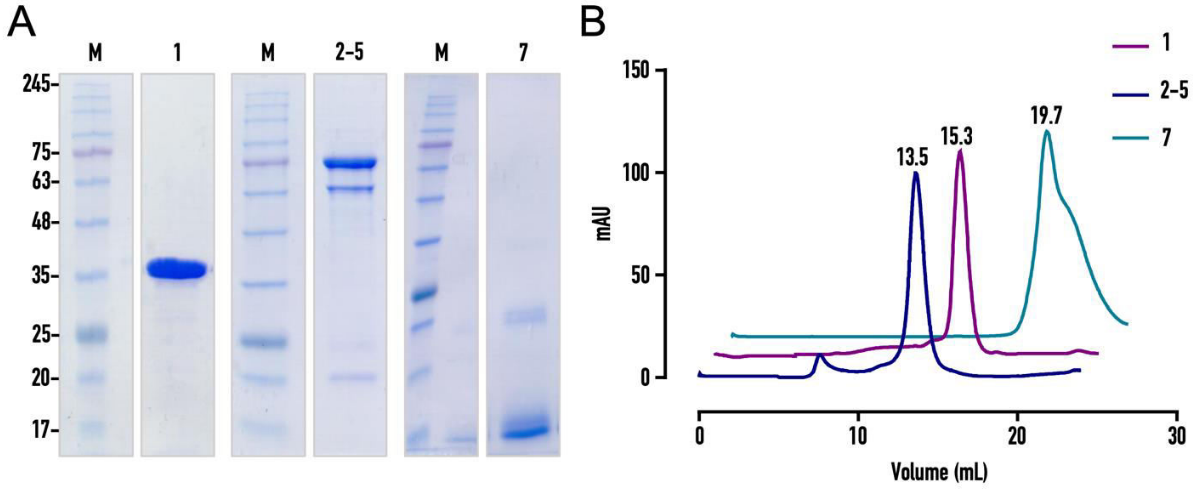

S3 Fig — Production and purification of ancillary IgAse domains. (A) SDS-PAGE analysis of constructs IgAse**1** (NTD alone), IgAse**2–5** (CD+WD+Oβ+ZBD1) and IgAse**7** (ZBD2 alone) produced for protein crystallography (see also Suppl. Table S1). **(B)** SEC analysis using a Superdex 200 10/300 GL column of the protein samples shown in (A).
