# Supplementary material for: Correction: Biochemical and structural characterization of the human gut microbiome metallopeptidase IgAse provides insight into its unique specificity for the Fab’ region of IgA1 and IgA2
Source: PLoS Pathog. 2025 Dec 4;21(12):e1013742. doi: 10.1371/journal.ppat.1013742 (PMC12677558; doi:10.1371/journal.ppat.1013742)
Supplement: S5 Fig — (PDF) [file ppat.1013742.s007.pdf]

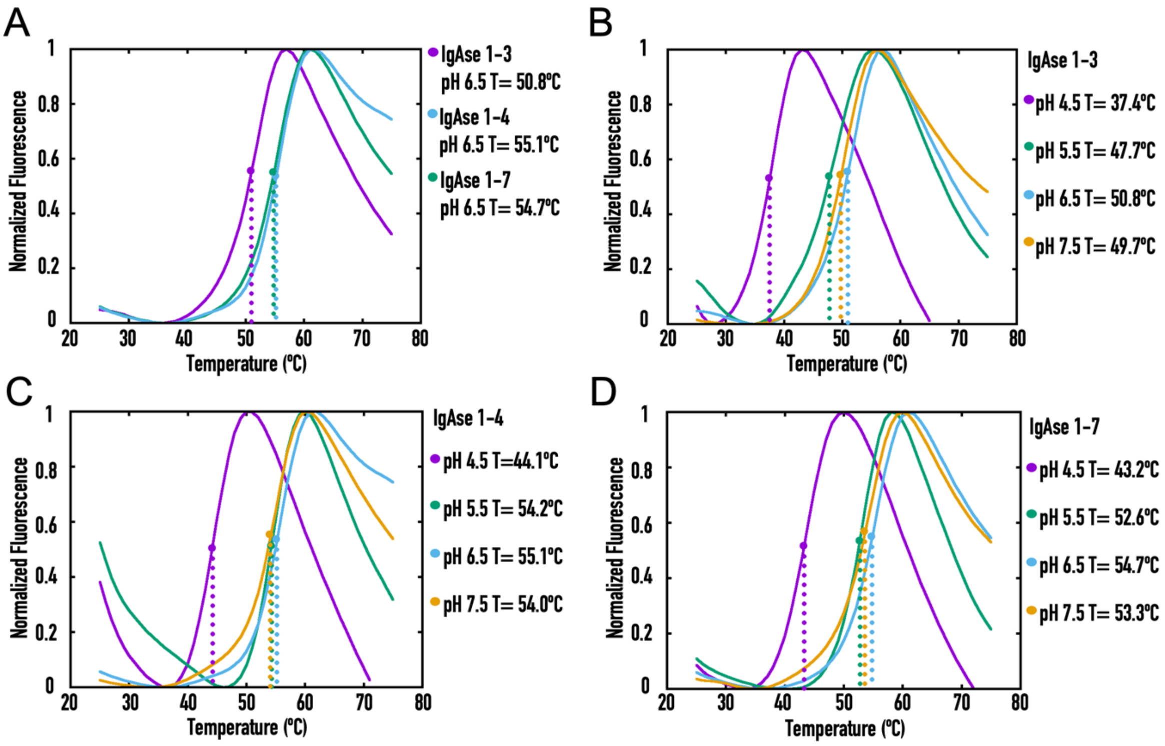

S5 Fig — Differential scanning fluorimetry analysis. **(A)** Thermal stability comparison at pH 6.5 for IgAse**1–7** (green), IgAse**1–4** (blue), and IgAse**1–3** (purple), as shown in Fig. 2D. Additional thermal denaturation curves at pH 4.5 (purple), 5.5 (green), 6.5 (blue), and 7.5 (yellow) are shown for IgAse**1–3 (B)**, IgAse**1–4 (C)**, and IgAse**1–7 (D)**, revealing a clear pH dependency.
