# Supplementary material for: Correction: Biochemical and structural characterization of the human gut microbiome metallopeptidase IgAse provides insight into its unique specificity for the Fab’ region of IgA1 and IgA2
Source: PLoS Pathog. 2025 Dec 4;21(12):e1013742. doi: 10.1371/journal.ppat.1013742 (PMC12677558; doi:10.1371/journal.ppat.1013742)
Supplement: S6 Fig — (PDF) [file ppat.1013742.s008.pdf]

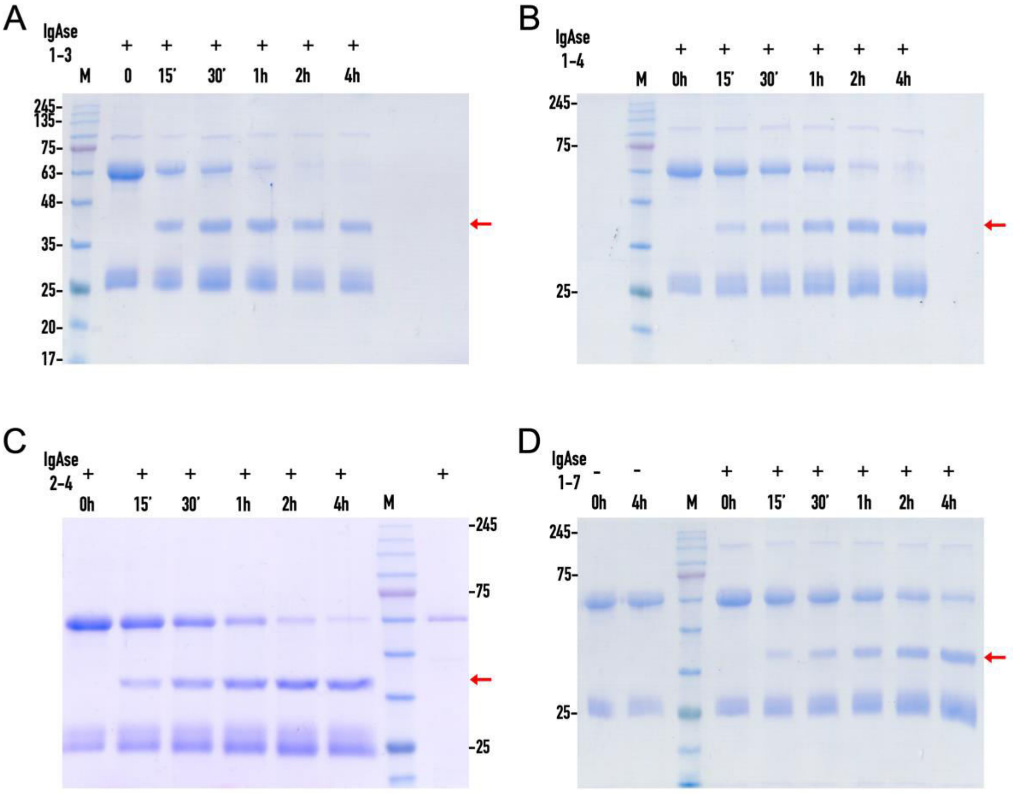

S6 Fig — Time-dependent IgA cleavage by IgAse. Reducing SDS-PAGE analysis of human plasma IgA (2 μM) incubated with (+) IgAse**1–3 (A)**, IgAse**1–4 (B)**, IgAse**2–4 (C)**, and IgAse**1–7 (D)** (all at 0.1 μM) at multiple time points. Cleavage at the HC hinge region results in a fragment indicated by a red arrow. Note that the right lane in **(C)** depicts IgAse**2–4** at 0.2 μM to demonstrate comigration with the intact IgA heavy chain. This suggests that the remaining band at 4 hours most likely represents IgAse**2–4** and not non-cleaved IgA. Finally, non-treated IgA incubated without IgAse (-) at 0 and 4 hours is shown in *lanes* 1 and 2 of **(D)**.
