# Supplementary material for: Correction: Biochemical and structural characterization of the human gut microbiome metallopeptidase IgAse provides insight into its unique specificity for the Fab’ region of IgA1 and IgA2
Source: PLoS Pathog. 2025 Dec 4;21(12):e1013742. doi: 10.1371/journal.ppat.1013742 (PMC12677558; doi:10.1371/journal.ppat.1013742)
Supplement: S9 Fig — (PDF) [file ppat.1013742.s011.pdf]

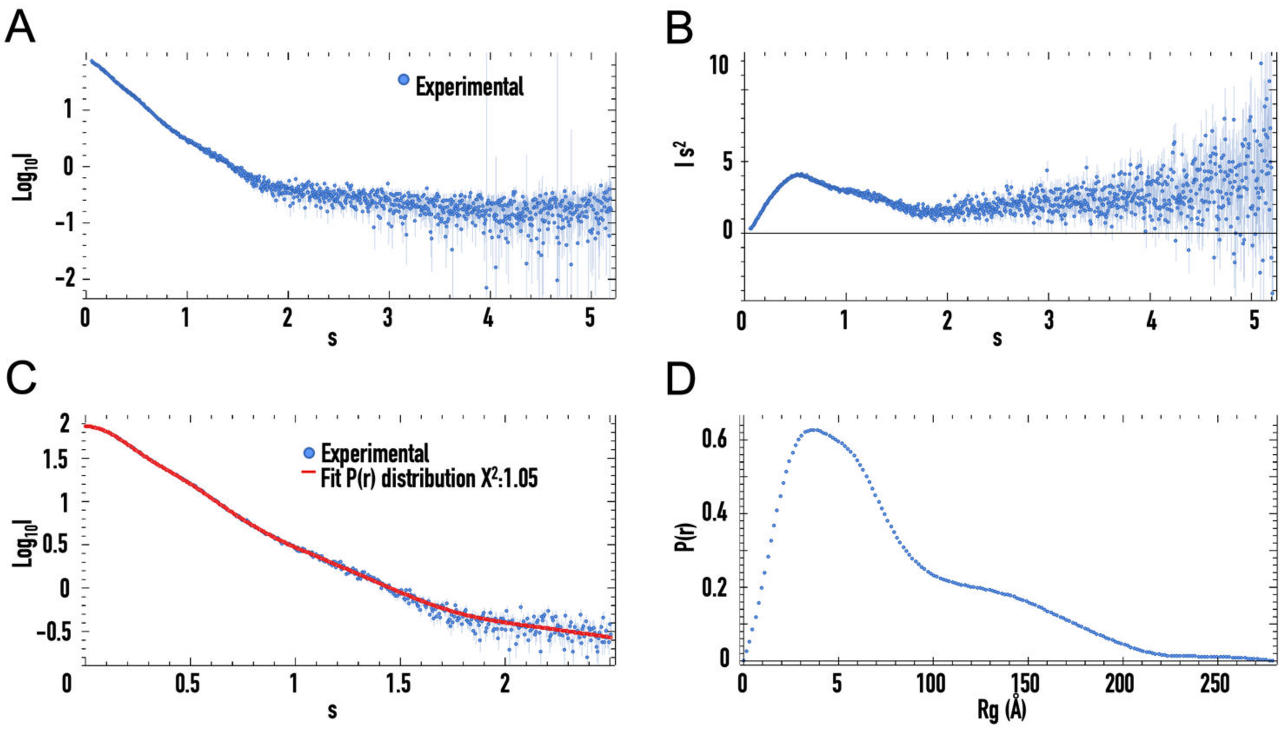

S9 Fig — SEC-SAXS analysis of IgAse1–7 using the full data range. (A) Experimental SEC-SAXS profile of IgAse**1–7** (blue). **(B)** Normalized Kratky plot derived from the SAXS profile shown in (A). **(C)** P(r) distribution fit (red curve) to the experimental SAXS data (blue curve) (χ^2 =1.05). **(D)** P(r) distribution showing a maximum linear dimension (d_{max}) of 278 Å.
