# Supplementary material for: Correction: Biochemical and structural characterization of the human gut microbiome metallopeptidase IgAse provides insight into its unique specificity for the Fab’ region of IgA1 and IgA2
Source: PLoS Pathog. 2025 Dec 4;21(12):e1013742. doi: 10.1371/journal.ppat.1013742 (PMC12677558; doi:10.1371/journal.ppat.1013742)
Supplement: S10 Fig — Reducing SDS-PAGE analysis showing quantitative cleavage in the hinge region (lane 4, red asterisk) of C-terminally truncated wild-type (WT) IgA2Δ3 (lane 3) after overnight incubation with IgAse1–4 (lane 1). In contrast, mutants T84R/S94Y (lanes 5 and 6) and D96R/S94Y/T98W (lanes 7 and 8) are not cleaved. Lane 2 depicts the BlueStar Plus Molecular Weight ladder. (PDF) [file ppat.1013742.s012.pdf]

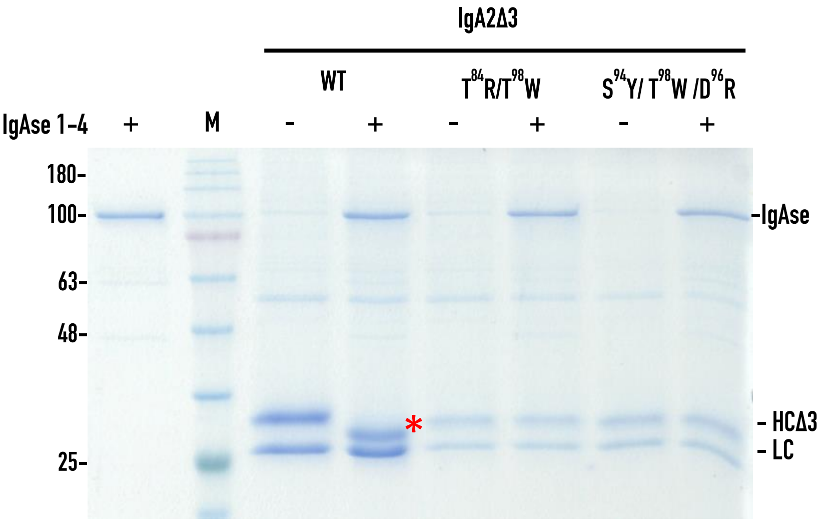

S10 Fig — IgAse activity analysis against wild-type and mutant IgA2Δ3. Reducing SDS-PAGE analysis showing quantitative cleavage in the hinge region (*lane* 4, red asterisk) of C-terminally truncated wild-type (WT) IgA2Δ3 (*lane* 3) after overnight incubation with IgAse**1–4** (*lane* 1). In contrast, mutants $T^{84}R/T^{98}W$ (*lanes* 5 and 6) and $S^{94}Y/T^{98}W/D^{96}R$ (*lanes* 7 and 8) are not cleaved. *Lane* 2 depicts the BlueStar Plus Molecular Weight ladder.
