# Supplementary material for: Correction: Biochemical and structural characterization of the human gut microbiome metallopeptidase IgAse provides insight into its unique specificity for the Fab’ region of IgA1 and IgA2
Source: PLoS Pathog. 2025 Dec 4;21(12):e1013742. doi: 10.1371/journal.ppat.1013742 (PMC12677558; doi:10.1371/journal.ppat.1013742)
Supplement: S1 Table — (PDF) [file ppat.1013742.s013.pdf]

S1 Table — IgAse constructs assayed.

Construct	Cloned	Expressed	Purified		Crystallography		Cryo-EM	
			IMAC	SEC	Crystals	Structure	Grids	Structure
**1**	✓	✓	✓	✓	✓	x	-	-
**1-2**	✓	✓	✓	x	-	-	-	-
**1-3**	✓	✓	✓	✓	✓	x	-	-
**1-4**	✓	✓	✓	✓	x	-	✓	x
**1-7**	✓	✓	✓	✓	x	-	✓	✓ [a]
**2**	✓	✓	✓	x	-	-	-	-
**2-4**	✓	✓	✓	✓	✓	✓	-	-
**2-5**	✓	✓	✓	✓	x	-	-	-
**5**	✓	x	-	-	-	-	-	-
**5-7**	✓	x	-	-	-	-	-	-
**6**	✓	x	-	-	-	-	-	-
**7**	✓	✓	✓	✓	x	-	-	-

[a] Only domains 1–4 were resolved in the SPA map.
