# Supplementary material for: Correction: Biochemical and structural characterization of the human gut microbiome metallopeptidase IgAse provides insight into its unique specificity for the Fab’ region of IgA1 and IgA2
Source: PLoS Pathog. 2025 Dec 4;21(12):e1013742. doi: 10.1371/journal.ppat.1013742 (PMC12677558; doi:10.1371/journal.ppat.1013742)
Supplement: S2 Table — (PDF) [file ppat.1013742.s014.pdf]

**S2 Table — Crystallographic data.**	
Sample	***T. ramosa IgAse2–4 (328 – 876+E^{540}A)***
Beam line (synchrotron)	BL13 XALOC (ALBA)
Detector	Pilatus3 X 6M
Space group/protomers per a.u. [a]	$P2_1$ / 1 (chain A)
Cell constants (a, b, c, in Å; β in °)	46.15, 87.83, 67.47, 96.1
Wavelength (Å)	0.9792
No. of measurements / unique reflections	342,449 / 53,460
Resolution range (Å) (outermost shell) [b]	45.9 – 1.75 (1.86 – 1.75)
Completeness (%)	99.1 (96.0)
R_{merge} [c]	0.077 (1.563)
R_{meas} [c]	0.083 (1.750)
CC($^1/_2$) [c]	0.999 (0.458)
Average intensity [d]	13.3 (1.2)
B-Factor (Wilson) (Å^2)	39.5
Aver. Multiplicity	6.4 (4.9)
Processing software	*Xds* / *Xscale*
No. of reflections used in refinement [in test set]	52,759 [701]
Crystallographic R_{factor}/free R_{factor}	0.178 / 0.213
F_{obs} , F_{calc} correlation [test set]	0.968 [0.955]
Residues with RSRZ [a] > 2	4 (0.7%)
No. of protein residues/atoms/solvent molecules/ non-covalent ligands	548 / 4409 / 430 / 2 Zn^{2+}, 1 FMT, 1 PG4, 8 EDO
Rmsd from target values	
bonds/angles/chirality/planarity	0.012 / 1.299 / 0.078 / 0.010
Average B-factors (Å^2): protein/ligands/solvents	35.4 / 52.0 / 45.1
All-atom contacts and geometry analysis	
Protein residues	
in favoured Ramachandran regions/outliers/all residues	533 (96.9%) / 0 / 550
with outlying rotamers/bonds/angles/chirality/planarity/Cβ	5 (1.0%) / 0 / 0 / 0 / 0 / 0
All-atom clashes/clashscore/Molprobity score	15 / 1.6 / 1.2
Protein residues in multiple conformations	2 (0.4%)
PDB access code	9I4Z

[a] Abbreviations: a.u., crystallographic asymmetric unit; FMT, formate ($HCOO^{\ominus}$); PG4, tetraethylene glycol ($OH–[CH_2–CH_2–O]_4–H$); EDO, ethylene glycol ($OH–CH_2–CH_2–OH$); RSRZ, real space R_{value} normalised against residue type and resolution (Fourier-map outliers). [b] Values in parentheses refer to the outermost resolution shell. [c] For definitions, see [1]. [d] Average intensity is $< I/\sigma(I) >$ of unique reflections after merging according to *Xscale* [2]. Validation according to the wwPDB Deposition Service (https://deposit-1.wwpdb.org/deposition).
