# Supplementary material for: Correction: Biochemical and structural characterization of the human gut microbiome metallopeptidase IgAse provides insight into its unique specificity for the Fab’ region of IgA1 and IgA2
Source: PLoS Pathog. 2025 Dec 4;21(12):e1013742. doi: 10.1371/journal.ppat.1013742 (PMC12677558; doi:10.1371/journal.ppat.1013742)
Supplement: S3 Table — (PDF) [file ppat.1013742.s015.pdf]

S3 Table — Cryo-electron microscopy single-particle analysis.

Microscope / voltage (kV)	Glacios / 200	Krios / 300
Synchrotron site	ALBA	ESRF
Magnification (nominal)	150,000×	165,000×
Electron dose (e^-/pixel/s)	7.1	7.2
Exposure time (s) / total dose per movie (e^-/Å^2)	6.0 / 47.5	4.8 / 60.0
Camera	Falcon 4	Falcon 4i+SelectrisX filter
Defocus range /steps (μm)	-1.5 to -2.5/0.2	-0.8 to -2.0 / 0.2
Pixel size (Å/pixel)	0.93	0.73
Movies used (no.)	4989	18,729
Symmetry	C1	C1
Final no. of particles	114,990	312,476
Map resolution (Å) at FSC 0.143 / 0.5	≥7.1 / ≥8.0	2.81 / 3.16
Processing software	*CryoSPARC*	*CryoSPARC/DeepEMhancer*
Map values max/min/average/stand. dev.		0.423 / -0.222 / 0.000 / 0.010
Recommended contour level		0.029
Map size/dimensions (both a=b=c)/angles (α=β=γ)		233.6 / 320 / 90
Box dimensions (Å)		73.7, 85.4, 110.2
Resolution estimates (Å) (masked/unmasked):		
FSC half maps (0.143)		2.7 / 2.8
FSC model (0.143; 0.5)		2.6; 2.8 / 2.6; 2.6
EMRinger score		4.99
Map-model fit (level 0.029) Q-score/atom inclusion		0.568 / 0.986
CC (mask) / CC (volume) / CC (peaks)		0.88 / 0.87 / 0.80
No. of protomers/residues/atoms/solvents		1 (chain A) / 845 / 7043 / 151 /
non-covalent ligands		3 Zn^{2+}, 1 GOL, 7 AZI
Rmsd from target values		
bonds/angles/chirality/planarity		0.006 / 0.875 / 0.050 / 0.008
Average B-factors (Å^2): protein/ligands/solvents		89.6 / 103.9 / 88.7
All-atom contacts and geometry analysis		
Protein residues in/with		
favoured Ramachandran regions/outliers/all residues		836 (97%)/ 0 / 859
outlying rotamers/bonds/angles/chirality/planarity/Cβ		41 (5.4%) / 0 / 0 / 0 / 0 / 0
All-atom clashes/clashscore/Molprobity score		126 / 9.3 / 2.2
Protein residues in multiple conformations		16 (1.9%)
EMDB access code		EMD-52972
PDB access code		9QA6

Abbreviations: AZI, azide ($N^{\ominus}$=$N^{\oplus}$=$N^{\ominus}$); GOL, glycerol (CH_2OH–CH(OH)-CH_2OH). Validation against the *CryoSPARC* raw map according to the wwPDB Deposition Service (https://deposit-1.wwpdb.org/deposition) and to the *Comprehensive Validation* protocol within *Phenix*.
