# Supplementary material for: Correction: Biochemical and structural characterization of the human gut microbiome metallopeptidase IgAse provides insight into its unique specificity for the Fab’ region of IgA1 and IgA2
Source: PLoS Pathog. 2025 Dec 4;21(12):e1013742. doi: 10.1371/journal.ppat.1013742 (PMC12677558; doi:10.1371/journal.ppat.1013742)
Supplement: S4 Table — (PDF) [file ppat.1013742.s016.pdf]

**S4 Table — SEC-SAXS data collection and derived parameters for IgAse1–7.**	
**Instrumentation**	
Beamline	BM-29 (ESRF)
Beam geometry	Point-collimated
Detector / wavelength (nm)	Pilatus3 2M (Dectris) in vacuum / 0.99
Sample-to-detector distance (m)	2.867
Exposure time (s) / frames	4 / 250
Measured q range (nm^{-1})	0.007–0.500
SEC chromatography column	Superose 6 Increase 5/150 GL
Flow rate (mL min^{-1})	0.2
Temperature (K)	293
**Software**	
Data reduction / processing	*FreeSAS / Chromixs*
Guinier /IFT analysis	*BioXTAS Raw /BioXTAS Raw (Gnom)*
Ensemble generation and fitting	*Eom*
Graphic representation	*PyMOL*
**Data collection and derived parameters**	
Sample concentration (mg mL^{-1})	4
Frames/qmin (nm^{-1})/qmax (nm^{-1})	6–475 / 0.0866 / 2.5049
Calculated monomeric (M_r)	127.9
I(0) (AU) from *P(r) /* from Guinier	73.0 ± 0.3 / 70.2 ± 0.2
R_g (nm) from *P(r) /* from Guinier	6.43 ± 0.06 / 5.65 ± 0.03
D_{max} (Å)	280
Molecular mass (M_r) (Bayesian)	118.8
SASBDB database entry	SAS6704
