# Supplementary material for: Correction: Biochemical and structural characterization of the human gut microbiome metallopeptidase IgAse provides insight into its unique specificity for the Fab’ region of IgA1 and IgA2
Source: PLoS Pathog. 2025 Dec 4;21(12):e1013742. doi: 10.1371/journal.ppat.1013742 (PMC12677558; doi:10.1371/journal.ppat.1013742)
Supplement: S5 Table — (PDF) [file ppat.1013742.s017.pdf]

S5 Table — Primers employed for cloning.

Construct	Residue range [a]	MW (kDa)	Primer	Overhang [b]	Binding region
**IgAse variants**					
**1**	A31–E319	32	F	ATCGCATATG	GCGAGCAAGCCGGACATCAAAG
			R	ATATCTCGAG	TTCCGCCGGCTCGCTAATGG
**1–2**	A31–V631	69	F	ATCGCATATG	GCGAGCAAGCCGGACATCAAAG
			R	ATCGCTCGAG	CACCAGTTGGCTCATACGCTTAAAAC
**1–3**	A31–Q807	89	F	ATCGCATATG	GCGAGCAAGCCGGACATCAAAG
			R	ATCGCTCGAG	CTGGGTTTCGGTGTTATCGTCC
**1–4**	A31–N876	97	F	ATCGCATATG	GCGAGCAAGCCGGACATCAAAG
			R	ATCGCTCGAG	GTTTTTATAGTAATAGGTAACCACG
**1–7**	A31-I1166	128	F	ATCGCATATG	GCGAGCAAGCCGGACATCAAAG
			R	ATCGCTCGAG	AATCTCAACCGCGCTACGTTTGTAG
**2**	S328–V631	36	F	ATATCATATGGGA	AGCACCGAGCAGAGCATCCA
			R	ATCGCTCGAG	CACCAGTTGGCTCATACGCTTAAAAC
**2–4**	S328–N876	64	F	ATATCATATGGGA	AGCACCGAGCAGAGCATCCA
			R	ATCGCTCGAG	GTTTTTATAGTAATAGGTAACCACG
**2–5**	S328–T1006	77	F	ATATCATATGGGA	AGCACCGAGCAGAGCATCCA
			R	ATATCTCGAG	ACCGGTGCTCGGAATCGGC
**5**	K877–T1006	15	F	ATATCATATG	AAGAACGAGGAACACACCCACAAC
			R	ATATCTCGAG	ACCGGTGCTCGGAATCGGC
**5–7**	K877–G1170	32	F	ATATCATATG	AAGAACGAGGAACACACCCACAAC
			R	ATATCTCGAG	ACCGGTCGCCGGAATCTCAAC
**6**	G1007–T1097	10	F	ATATCATATG	ACCGAACTGAAGATCATTGCGGG
			R	ATATCTCGAG	GGTACGCACGGTCAGGTTCG
**7**	S1100–G1170	9	F	ATATCATATG	AGCGGTCACATTCACGACTACG
			R	ATATCTCGAG	ACCGGTCGCCGGAATCTCAAC
**IgA variants**					
**IgA2 (LC) Phl p7**	Q1–S217	22	F	ATATGGTGAC	CAGTCTGCCCTGACTCAGC
			R	AATATGCGATCGC	CTATGAACATTCTGTAGGGGCCT
**IgA2Δ1 (HC) Phlp 7**	Q1–P444	49	F	ATATCCCGGG	ATGGACTGGACCTGGAGGATC
			R	ATATGCGATCGC	GGGTTTACCCGCCAAGCGG
**IgA2Δ2 (HC) Phl p7**	Q1–S330	35	F	ATATCCCGGG	ATGGACTGGACCTGGAGGATC
			R	ATATGCGATCGC	GGATTTTGTGATGTTGGCGGTTAG
**IgA2Δ3 (HC) Phl p7**	Q1–P229	24	F	ATATCCCGGG	ATGGACTGGACCTGGAGGATC
			R	ATATGCGATCGC	TGGGGGAGGTGGGGGAAC

[a] Amino-acid numbering of IgA variants starts with Q^1 immediately after the signal peptide.
[b] Underlined nucleotides indicate restriction-enzyme cleavage sites for molecular cloning.
