# Supplementary material for: Correction: Biochemical and structural characterization of the human gut microbiome metallopeptidase IgAse provides insight into its unique specificity for the Fab’ region of IgA1 and IgA2
Source: PLoS Pathog. 2025 Dec 4;21(12):e1013742. doi: 10.1371/journal.ppat.1013742 (PMC12677558; doi:10.1371/journal.ppat.1013742)
Supplement: S8 Fig — (PDF) [file ppat.1013742.s010.pdf]

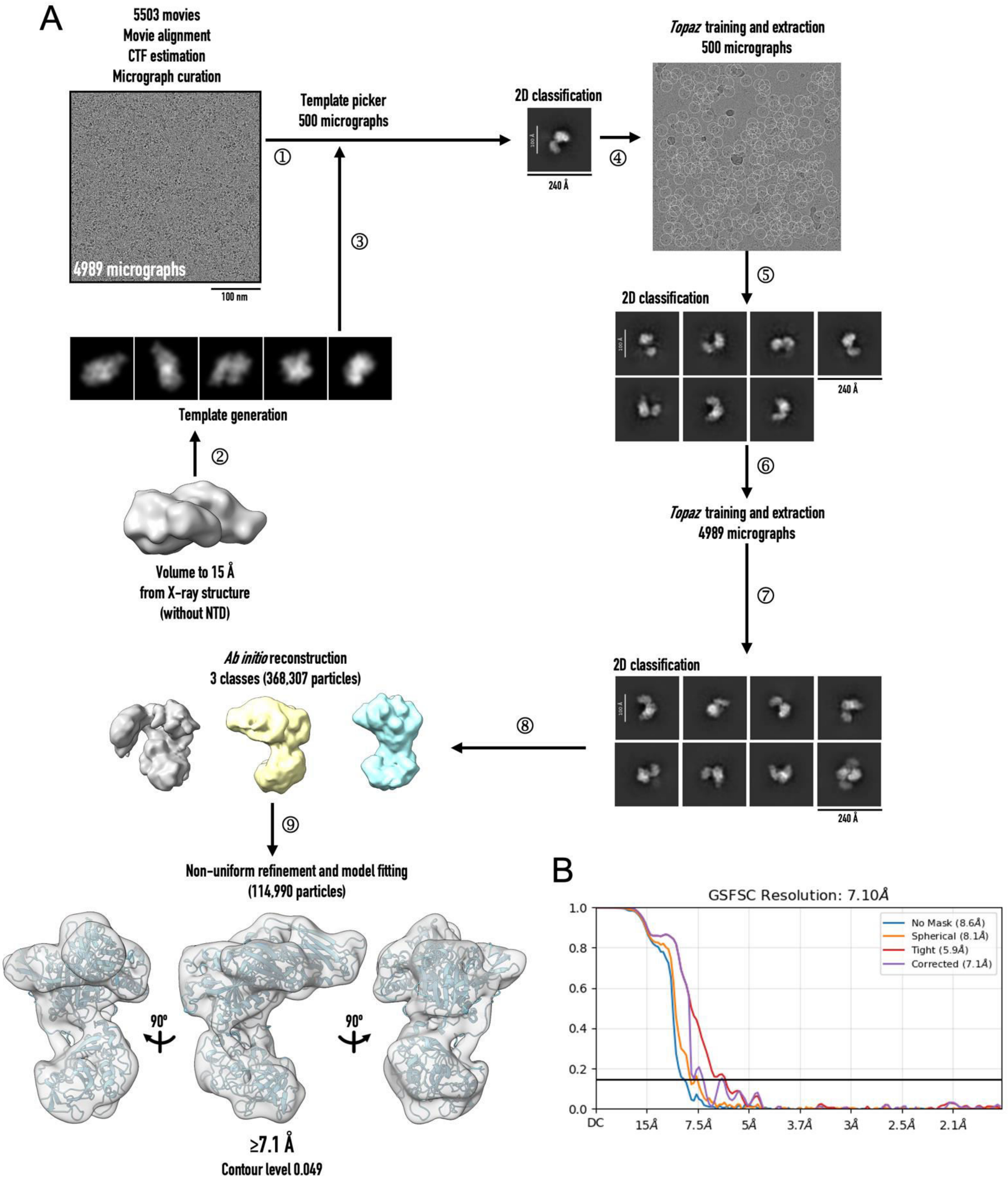

S8 Fig — Cryo-EM data processing. (A) Flowchart depicting cryo-electron microscopy single-particle analysis and image processing of the medium/low-resolution movies from a 200-kV Glacios microscope. **(B)** Gold-standard (0.143) Fourier Shell Correlation (GSFSC) curves. Model fitting was performed using our X-ray structure (CD+WD+Oβ) and the NTD plus connecting linker as modelled by *AlphaFold3*.
